# Metabolic adaptation of two in silico mutants of *Mycobacterium tuberculosis* during infection

**DOI:** 10.1186/s12918-017-0496-z

**Published:** 2017-11-21

**Authors:** Víctor A. López-Agudelo, Andres Baena, Howard Ramirez-Malule, Silvia Ochoa, Luis F. Barrera, Rigoberto Ríos-Estepa

**Affiliations:** 1 Grupo de Bioprocesos, Departamento de Ingeniería Química, Universidad de Antioquia UdeA, Calle 70 No. 52-21, Medellín, Colombia; 2 Grupo de Inmunología Celular e Inmunogenética (GICIG), Facultad de Medicina, Universidad de Antioquia UdeA, Calle 70 No. 52-21, Medellín, Colombia; 3 Departamento de Microbiología y Parasitología, Facultad de Medicina, Universidad de Antioquia UdeA, Calle 70 No. 52-21, Medellín, Colombia; 4 Escuela de Ingeniería Química, Universidad del Valle, A.A. 25360, Cali, Colombia; 5 Grupo de investigación en Simulación, Diseño, Control y Optimización de Procesos (SIDCOP), Departamento de Ingeniería Química, Universidad de Antioquia UdeA, Calle 70 No. 52-21, Medellín, Colombia; 6 Instituto de Investigaciones Médicas, Facultad de Medicina, Universidad de Antioquia UdeA, Calle 70 No. 52-21, Medellín, Colombia

**Keywords:** *Mycobacterium tuberculosis*, Phenotypic phase plane analysis, Genome-scale metabolic modeling, Metabolic reprogramming

## Abstract

**Background:**

Up to date, *Mycobacterium tuberculosis* (Mtb) remains as the worst intracellular killer pathogen. To establish infection, inside the granuloma, Mtb reprograms its metabolism to support both growth and survival, keeping a balance between catabolism, anabolism and energy supply. Mtb knockouts with the faculty of being essential on a wide range of nutritional conditions are deemed as target candidates for tuberculosis (TB) treatment. Constraint-based genome-scale modeling is considered as a promising tool for evaluating genetic and nutritional perturbations on Mtb metabolic reprogramming. Nonetheless, few *in silico* assessments of the effect of nutritional conditions on Mtb’s vulnerability and metabolic adaptation have been carried out.

**Results:**

A genome-scale model (GEM) of Mtb, modified from the H37Rv iOSDD890, was used to explore the metabolic reprogramming of two Mtb knockout mutants (*pfkA*- and *icl*-mutants), lacking key enzymes of central carbon metabolism, while exposed to changing nutritional conditions (oxygen, and carbon and nitrogen sources). A combination of shadow pricing, sensitivity analysis, and flux distributions patterns allowed us to identify metabolic behaviors that are in agreement with phenotypes reported in the literature. During hypoxia, at high glucose consumption, the Mtb *pfkA*-mutant showed a detrimental growth effect derived from the accumulation of toxic sugar phosphate intermediates (glucose-6-phosphate and fructose-6-phosphate) along with an increment of carbon fluxes towards the reductive direction of the tricarboxylic acid cycle (TCA). Furthermore, metabolic reprogramming of the *icl*-mutant (*icl1*&*icl2*) showed the importance of the methylmalonyl pathway for the detoxification of propionyl-CoA, during growth at high fatty acid consumption rates and aerobic conditions. At elevated levels of fatty acid uptake and hypoxia, we found a drop in TCA cycle intermediate accumulation that might create redox imbalance. Finally, findings regarding Mtb-mutant metabolic adaptation associated with asparagine consumption and acetate, succinate and alanine production, were in agreement with literature reports.

**Conclusions:**

This study demonstrates the potential application of genome-scale modeling, flux balance analysis (FBA), phenotypic phase plane (PhPP) analysis and shadow pricing to generate valuable insights about Mtb metabolic reprogramming in the context of human granulomas.

**Electronic supplementary material:**

The online version of this article (10.1186/s12918-017-0496-z) contains supplementary material, which is available to authorized users.

## Background

Tuberculosis (TB) is the world’s leading infectious cause of death, with 10.4 million new active cases in 2015 and mortality of 1.8 million people [1]. The etiological agent of TB, *Mycobacterium tuberculosis* (Mtb), is capable of replicating and surviving for decades within host granulomas, including caseating, fibrotic and cavitating lesions, each providing a diverse array of nutritional microenvironments [2].

During the infection, it has been proposed that Mtb makes a shift between replicative to non-replicative states [3]. During replicative states, Mtb is predicted to access glucose and triacylglycerides as main carbon sources in aerobic conditions inside macrophages [4]. As the infection continues, there is an increased immune activation of host cells, which attracts mononuclear cells and T lymphocytes to the infection site to encapsulate the bacteria; this structure is called granuloma, the hallmark of TB [5]. At this point, normal aerobic respiration is limited (hypoxia), the bacterium induces dysregulation of host’s lipid metabolism, which triggers the formation of foamy macrophages [6]. Through this non-replicative state, Mtb metabolism relies mainly on fatty acids and lipids. Consequently, Mtb reprograms its metabolism to support both growth and survival. In this scenario, the cell struggles to maintain a metabolic balance among catabolism, anabolism and energy supply [7]. A recent study that integrates metabolomics and transcriptomics data, showed that Mtb uses different nutrients during macrophage infection, with a notorious uptake and utilization of lipids [8].

Over the past few years, a limited number of experimental studies have aimed at describing the essential roles of enzymes of central carbon metabolism (CCM) in the physiology of Mtb [9–16]. However, our understanding of Mtb’s metabolic reprogramming is still scarce, as the essentiality of such enzymes is conditional to the nutritional environment of the organism, and their complete phenotypic/functional characterization is time-consuming and laborious.

A straightforward approach for predicting the effect of genetic and environmental perturbations on metabolism is the genome-scale constraint-based modeling, using flux balance analysis (FBA) [17, 18]. Applications of these models for studying Mtb metabolism has been promoted since 2007 [19–25]. Models have been used to simulate growth, metabolic phenotypes, synergistic drug inhibition, and identification of potential drug targets, among others. Although FBA has been used for obtaining meaningful global predictions of gene-essentiality in a variety of model organisms and environments [20, 26–29], the intracellular metabolic adaptation of those knockouts under changing nutritional environments has never been explored in deep.

In this study, an improved genome-scale model (GEM) of Mtb was used to explore in silico the essentiality/lethality of two Mtb mutants (*pfkA*-mutant and *icl*-mutant) under changing nutritional environments, specifically, normoxia/hypoxia and high and low consumption rates of glucose and propionate (glycolytic and anaplerotic substrates, respectively). Here, we demonstrate the potential application of genome-scale modeling, FBA flux distributions, phenotypic phase plane analysis and shadow prices to generate valuable insights concerning Mtb metabolic reprogramming, and to point out the conditions that favor or disfavor the survival of those wild type and Mtb mutants, in the context of human granulomas.

## Methods

### Metabolic network of *Mycobacterium tuberculosis*

The Mtb H37Rv iOSDD890 metabolic network model, proposed by Vashisht and colleagues, was used as the starting point [30]. The model consists of 1152 reactions, 961 metabolites, and 890 genes. The *Model and Constraint Consistency Checker* (MC3) algorithm was run under MATLAB R2013a to identify dead-end metabolites, single-connected metabolites, and zero-flux reactions [31]. For improving the Mtb metabolic network, some gaps for β-oxidation of fatty acids and transport reactions were filled. Also, reactions from the cholesterol degradation pathway were added into the iOSDD890 model [24]. The improved iOSDD890 metabolic model encompassed 1265 reactions, 1021 metabolites, and 922 genes.

### Flux balance analysis

Using the extended model, we performed metabolic flux balance studies under specific conditions and constraints. FBA uses linear optimization to determine the steady-state reaction flux distributions in a metabolic network, by maximizing (or minimizing) an objective function, such as ATP production, growth rate, or metabolite production. The metabolic system is described using the well-known stoichiometric matrix **S**_**m** × **n**_, relating the flux rates of enzymatic reactions **v**_**n** × 1_ to time derivatives of metabolic concentrations **x**_**m** × 1_ as **dx**/**dt** = **S**. **v** where **v** = [**v**_1_, **v**_2_…**v**_**ni**_ **b**_1_ **b**_2_…**b**_**next**_]^***T***^; **v**_**i**_ are the internal fluxes; **b**_***i***_ represents the exchange fluxes in the system; ***n***_***i***_ is the number of internal metabolites and **next** is the number of external metabolites in the system. At steady state, **dx**/**dt** = **S**. **v** = 0. Therefore, the required flux distribution belongs to the null space of **S**. Since there are more reactions than metabolites, the system becomes underdetermined, thus entailing the formulation of a constrained optimization problem. The critical step is the definition of an objective function that captures the biochemical goal of the system [32, 33]. As the problem consists of a linear objective function and linear constraints, a Linear Programming (LP) problem arises as: max **c** ^**T**^ **v** ; s. t. **S**. **v** = 0**,** wherein ***c*** represents the objective function. COBRA Toolbox v2.0 synchronized with MATLAB and the Gurobi optimizer 5.6.3, was used for all FBA calculations.

### Phenotypic phase plane (PhPPs) analysis

PhPP analysis is a method for sensitivity analysis of GEMs [34, 35]. Here, the effect of the variation of two environmental parameters (e.g. glucose and oxygen uptake rates) on an objective cellular function is analyzed. To construct the PhPP diagram, the respective values of the environmental conditions under investigation are represented in a *x, y* plane and the optimal flux distribution is computed for all points in the plane. Shadow prices are calculated for the two-parameter space to demarcate regions of constant flux distributions (phenotypic phases). Phenotypic phases are drawn based on changes in these shadow prices. In addition, in the PhPP there is a line called *isocline*, which demarks zones of constant values of the objective function. The slope of isoclines within each phase of the PhPP is calculated from the shadow prices as follow:

1 $$ \upalpha =-\frac{\uppi_{\mathrm{x}}}{\uppi_{\mathrm{y}}} $$

Where *π* is the shadow price and *x* and *y* refer to the variables on the *x*- and *y*-axes. Phases with a negative *α* value means there is a dual limitation of the substrates (both contribute positively towards the objective function). Phases with a positive *α* value are termed “futile” phases; in these phases, one of the substrates is inhibitory towards obtaining the objective function, and this substrate will have a positive shadow price. Finally, if a phase has either horizontal or vertical isoclines, there exist single substrate limitations; the *α* value in this phase will be zero or infinite, respectively. Therefore, in that phase, the shadow price of one of the substrates goes to zero and thus has no value to the cell [34, 35].

### Optimization problem statement

For most system biology applications where FBA is used, the analysis lies on the maximization of biomass growth rate; in contrast, in the present study, we used a two-stage optimization approach, as described by Schuetz and colleagues and D’Huys and colleagues [32, 36]. Such approximation retrieves a unique and biologically meaningful solution to the FBA problem of biomass growth rate maximization, thus overcoming the problem of finding alternate optimal solutions. The first stage in the approach deals with the maximization of biomass growth rate (linear programming problem), whereas the second stage deals with the minimization of the sum of squares of all fluxes (non-linear programming problem) of the metabolic network model. The assumption underlying the minimization principle postulates that cells and whole organisms gain functional fitness by fulfilling their functions with minimal effort and thus assuring an efficient metabolic flux distribution [32, 37]. The value of biomass growth rate, obtained at the first stage, is used as a constraint during the second optimization stage. The first stage was run using Gurobi solver under COBRA Toolbox, while the second stage was solved using the MATLAB’s built-in *fmincon* solver. For all runs, the Gurobi feasibility tolerance was set to 10^−6^, and for the non-linear optimization, the algorithm was terminated when first order optimality was satisfied to within 10^−6^; the maximum constraint violation was also 10^−6^. The mathematical formulation of the whole optimization problem is shown in Eqs. (2)–(3). The sensitivity analysis, including the PhPP analysis and the calculation of shadow prices, was carried out by solving the LP problem, used in stage 1. In contrast, stage 2 was utilized for the computation of flux distributions in the phenotypes of interest.

2 $$ \mathrm{Stage}\;\mathsf{1}:\mathsf{\operatorname{Maximize}}\;\mathit{\mathsf{Z}}={\mathit{\mathsf{v}}}_{\mathit{\mathsf{biomass}}} $$

$$ \mathrm{Subject}\  \mathrm{to}:{\sum}_{\mathit{\mathsf{j}}=\mathsf{1}}^{\mathit{\mathsf{n}}}{\mathit{\mathsf{S}}}_{\mathit{\mathsf{ij}}}{\mathit{\mathsf{v}}}_{\mathit{\mathsf{j}}}=\mathsf{0};{\mathit{\mathsf{v}}}_{\mathit{\mathsf{j}}}^{\mathit{\mathsf{LB}}}\le {\mathit{\mathsf{v}}}_{\mathit{\mathsf{j}}}\le {\mathit{\mathsf{v}}}_{\mathit{\mathsf{j}}}^{\mathit{\mathsf{UB}}} $$

3 $$ \mathrm{Stage}\;\mathsf{2}:\mathsf{\operatorname{Minimize}}\kern0.24em {\sum}_{\mathit{\mathsf{j}}=\mathsf{1}}^{\mathit{\mathsf{n}}}{\mathit{\mathsf{v}}}_{\mathit{\mathsf{j}}}^{\mathsf{2}} $$

$$ \mathrm{Subject}\  \mathrm{to}:{\sum}_{\mathit{\mathsf{j}}=\mathsf{1}}^{\mathit{\mathsf{n}}}{\mathit{\mathsf{S}}}_{\mathit{\mathsf{ij}}}{\mathit{\mathsf{v}}}_{\mathit{\mathsf{j}}}=\mathsf{0};{\mathit{\mathsf{v}}}_{\mathit{\mathsf{j}}}^{\mathit{\mathsf{LB}}}\le {\mathit{\mathsf{v}}}_{\mathit{\mathsf{j}}}\le {\mathit{\mathsf{v}}}_{\mathit{\mathsf{j}}}^{\mathit{\mathsf{UB}}};{\mathit{\mathsf{v}}}_{\mathit{\mathsf{biomass}}}=\mathit{\mathsf{Z}} $$

### Simulation strategy for exploring the metabolic adaptation of two mutants of *Mycobacterium tuberculosis*

Simulations using the extended GEM of Mtb were carried out for exploring the metabolic adaptation of the bacteria to different oxygen and substrate availabilities after two-mutation perturbations. The first case study addressed the metabolic adaptation for a phosphofructokinase (*ΔpfkA)* mutant strain, growing in a medium with available glycerol, phosphate, ammonium, and trace elements, and under different oxygen and glucose uptake consumptions. The second case study, explored the metabolic adaptation of an isocitrate lyase (*Δicl1 & Δicl2)* mutant strain, growing on a medium with available phosphate, ammonium and trace elements, and on various propionate and oxygen availabilities.

For simulation purposes, as a first step, phenotype phase planes were built, and phenotypes of interest were identified (e.g. variation of glucose and oxygen uptake rates and variation of fatty acids and oxygen uptake rates, respectively, for each case study; for both instances, biomass growth rate was maximized). Furthermore, shadow prices were calculated for selected important metabolic intermediates that participate in the perturbed pathways. Finally, a pathway utilization analysis was carried out along with the characterization of flux distributions at specific conditions for each phenotype.

## Results and discussion

The identification and analysis of different metabolic phenotypes of the Mtb mutants comprised model formulation, sensitivity analysis, a solution of the optimization problem, analysis of results, and hypothesis generation. The complete strategy is presented in Additional file 1.

The GEM of Mtb iOSDD890 was used as starting point for this analysis [30]. Gene-protein-reaction associations, zero flux reactions, metabolic gaps and reaction reversibility, were checked by using the MC3 algorithm [31]. Accordingly, some network inconsistency issues related to gaps were identified. Based on these results, three noteworthy modifications were carried out in the iOSDD890 model.

I. Under the tested environmental conditions, it was identified that some reactions in the β-oxidation pathway within the iOSDD890 model had zero fluxes, and no grow could be achieved on those fatty acids. This condition was caused by the absence of reactions catalyzed by the ATP- dependent fatty acyl-CoA ligases in the iOSDD890 model; these steps are essential for fatty acid activation (conjugation with Coenzyme A) before β-oxidation. Therefore, we added 67 reactions related to fatty acyl-CoA ligases, transport, and exchange of some fatty acids, thus rendering a model able to represent cell growth on fatty acids.
II. It was detected that seven transport reactions were missing in the iOSDD890 model, so it cannot use neither acetate nor aminobenzoate, formate, oxoglutarate, oxalate, guanine or urea. Hence, the corresponding transport reactions were added, as some of them can be used by Mtb as sole carbon and nitrogen sources [38].
III. The iOSDD890 model missed the cholesterol degradation pathway. For this, 36 related reactions were added from another published model [24].

Additional information for new reactions and gene-protein-reaction (GPR) associations are included in Additional file 2. The final extended model, obtained after the modifications mentioned above, encompassed 1265 reactions, 1021 metabolites, and 922 genes (Additional file 3).

For both case studies, we modified the reaction exchange bounds to allow the Mtb model to run on a minimal culture medium. Then, we identified reactions and pathways that were perturbed, and defined biomass growth rate as the objective function to generate a working constraint-based model to be used in FBA.

### Granulomatous lesions during TB disease

Findings in humans and the non-human primate model of Mtb infection have indicated that granulomatous lesions are highly heterogeneous [39]. The diverse immunopathology of granulomas and cavities generates a plethora of microenvironments to which Mtb must adapt, affecting the replication, metabolism, bacterial subpopulations, and consequently their respective susceptibility to anti-TB drugs.

Granulomatous lesions in humans have different forms: (i) non-necrotizing granulomas characterized by a lymphocyte rim, macrophages and epithelioid histiocytes at the center. In this stage, the granuloma becomes highly vascularized [40, 41]. (ii) Necrotizing granuloma with a central necrosis surrounded by a layer of cellular infiltrates and a fibrotic rim. (iii) Granulomas with solid caseous centers characterized by a complete necrosis of immune cells with a disappeared nucleus and cellular contours, forming the caseum (cheese-like core). A solid caseum contains relatively low bacterial numbers and is delineated by layers of lymphocytes, activated macrophages surrounded by a collagen rim. (iv) Liquefied necrotic granulomas characterized by a fissured and fragmented caseous mass, with semi-liquid consistency and high extracellular bacillary numbers. (v) Cavities appear when remnants of liquefied lesions release their content into an airway; they are delineated by a fibrous capsule, with high extracellular and intracellular bacillary numbers. Additional factors such as hypoxia (defined as <4 μM O_2_ saturation or O_2_ tension lower than 10 mmHg), nutrient limitation, low pH and oxidative stress are thought to be present in caseating and necrotic lesions and the phagolysosome of infected macrophages [39, 42]. In caseous granulomas, lipids like triglycerides and cholesterol are abundant [43], which enables Mtb to adapt to a lipid-rich environment by the induction of cholesterol and triglyceride utilizing enzymes [44–46]. It is believed that this nutrient shift allows Mtb to acquire the poorly understood dormancy-like or persister-like phenotypes [47, 48]. From this, our interest was to explore the metabolic adaptations of the two Mtb mutants growing during shifts of oxygen and carbon sources (glucose and fatty acids), and to analyze it in the context of the Mtb under granulomatous environments.

### Case study I: In silico analysis of the metabolic adaptation of a *pfkA*-mutant of *Mycobacterium tuberculosis* to changes in oxygen and glucose uptake rates

**Fig. 1:**
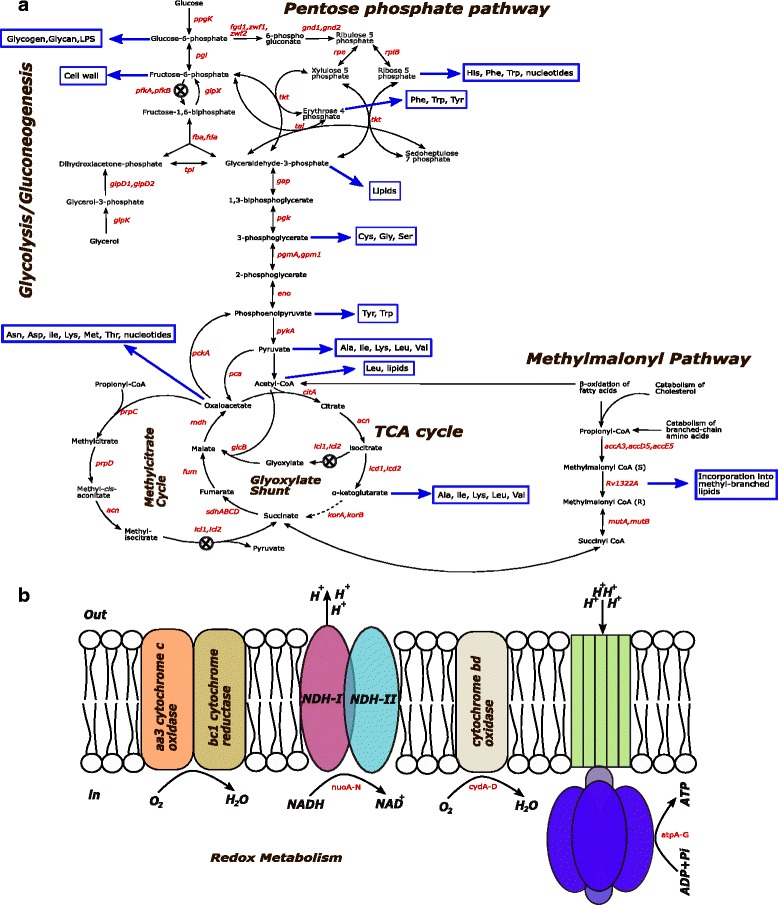
Key Metabolic pathways of Mtb during infection. **a** Central metabolism of Mtb. **b** Redox metabolism of Mtb. The X symbol represents the disrupted pathways for simulating the experimental conditions reported in the literature

PFKA is a key glycolytic enzyme that catalyzes the irreversible formation of fructose-1,6-biphosphate from fructose-6-phosphate (Fig. 1a). Although Mtb has two genes associated with PFK activity (*pfkA* and *pfkB*), functional studies of Phong and Colleagues (2013) suggested that *pfkA* might be responsible for 100% of the PFK activity under aerobic and hypoxic conditions. In contrast, *pfkB* overexpression does not lead to detectable PFK activity under the same conditions. Different researchers have argued that a conserved glycolysis pathway in Mtb is a sign of its capacity to use glycolytic substrates during infection [9, 10, 14–16].

**Fig. 2:**
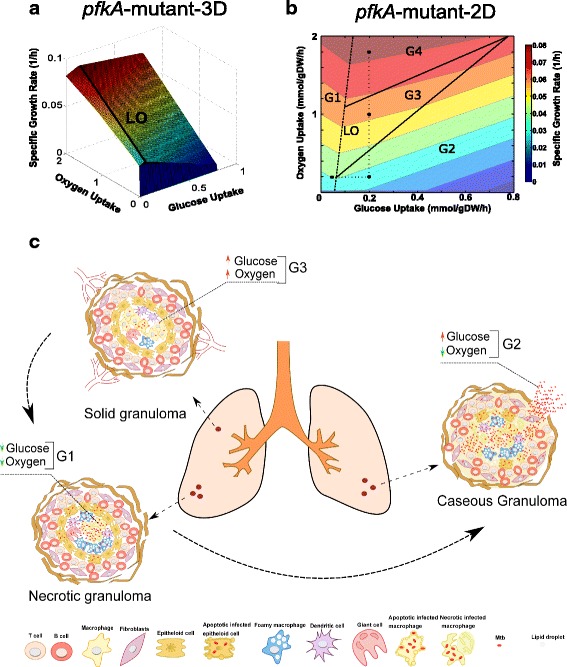
Metabolic adaptation of the *pfkA*-mutant of Mtb during shifts in glucose and oxygen concentration. Phases in the phenotypic phase plane (PhPP) are associated with hypothetical scenarios of nutrient availability inside granulomas. **a** 3D top view of the PhPP diagram while optimizing for growth. The line of optimality (LO) corresponds to the optimal relation between the two metabolic fluxes to obtain maximal Mtb growth. **b** 2D top view of the PhPP highlighting phases and isoclines. Black dots correspond to the substrate uptake rates used to simulate the metabolic flux distribution characterizing a given phase. The dotted lines depict constant substrate uptake rates kept from one phenotype to another. **c** Types of granulomas and nutritional environments experienced during Mtb infection

We simulated the metabolic adaptation of a null *pfkA*-mutant of Mtb using the modified iOSDD890 Mtb model. The in silico *pfkA*-mutant strain was generated by disrupting the glycolytic reaction step involved in the formation of fructose-1,6-biphosphate (ATP + fructose-6-phosphate → fructose 1,6 biphosphate + ADP) (Fig. 1a). To elucidate the effect of oxygen and glucose consumption on the Mtb*-*mutant growth rate, a phenotypic phase plane (PhPP) analysis for the *pfkA*-mutant was performed. We generated a three-dimensional graph showing the effect of substrate and oxygen uptake rate on Mtb growth (Fig. 2a). Four phases can be identified from the graph (changes in the slopes) representing different metabolic pathway utilization patterns or phenotypes. The continuous black line corresponds to the optimal relation between glucose and oxygen consumption to obtain the maximal Mtb growth (LO: line of optimality). In general, we observed that increments in oxygen uptake rate promote Mtb’s growth, and increments in glucose consumption partially inhibit Mtb growth in the *pfkA*-mutant.

By analyzing the three-dimensional graph of *pfkA*-mutant (Fig. 2b), we generated a two-dimensional graph that highlights the presence of isoclines (which define regions of different colors with the same constant growth rate values), when glucose and oxygen uptakes are varied.

### PhPP and shadow price analysis identify inhibitory substrate uptake conditions for an Mtb *pfkA*-mutant in the context of human granulomas

As observed in Fig. 2b, the PhPP of the *pfkA*-mutant is divided into four phases (G1-G4); each phase has a distinct metabolic pathway utilization pattern (same figures for Wild-type Mtb are shown in Additional file 4). G1 is characterized by a negative slope of isoclines, which means that, in this phase, both glucose and oxygen consumption are important for the Mtb’s growth. In contrast, phases G2, G3 and G4 are characterized by positive slopes of isoclines, which means that one of the varied substrates (glucose or oxygen) is inhibitory to Mtb growth. Wild-type Mtb had a different behavior in comparison with the *pfkA*-mutant; negative isoclines were observed in all phases (Additional file 4).

Black points in Fig. 2b show scenarios at specific conditions (glucose and oxygen uptake rates) in each phase. The black point in G1 represents Mtb inside a solid necrotic granuloma (with a hypoxic center) and poor vascularization (Fig. 2c) [42]. In this kind of lesions, Mtb induces the formation of lipid bodies and foamy macrophage phenotypes [6]. Therefore, in this scenario, we assumed that this intracellular Mtb-mutant is undergoing a metabolic transition from glucose to lipid consumption during quiescence. Hence, in G1, Mtb is oxygen deprived and consumes glucose at low rates (Fig. 2b). This G1 phase is characterized by the negative slope of the isoclines in both Wild-Type and pfkA-mutant; as expected, negative shadow prices were obtained for both glucose and oxygen (see Table 1); consequently, within this phase, increasing the availability of oxygen or glucose will increase biomass growth rate. Due to these restrictions, Mtb should be in a non-replicative state, and the access to carbon sources and oxygen might increase its metabolic activity.

**Table 1:** Shadow prices for different metabolites in Wild-type and *pfkA*-mutant strains exposed to various substrate concentrations

Metabolite	G1		G2		G3		G4	
	WT	Δ*pfkA*	WT	Δ*pfkA*	WT	Δ*pfkA*	WT	Δ*pfkA*
Oxygen	−0.0273	−0,0273	−0.0278	−0,0365	−0.0278	−0,0305	−0.0215	−0,0232
Glucose	−0.0601	−0.0601	−0.0500	**0.0274**	−0.0500	**0.0122**	−0.0484	**0.0029**
Glucose-6-P	−0.0601	−0,0601	−0.0500	**0,0274**	−0.0500	**0,0122**	−0.0484	**0,0029**
Fructose-6-P	−0.0601	−0,0601	−0.0500	**0,0274**	−0.0500	**0,0122**	−0.0484	**0,0029**
Ribose-5-P	−0.0501	−0,0501	−0.0435	**0,0061**	−0.0435	−0,0031	−0.0421	−0,0087
Xylulose-5-P	−0.0501	−0,0501	−0.0435	**0,0061**	−0.0435	−0,0031	−0.0421	−0,0087
Mannose-1-P	−0.0601	−0,0601	−0.0500	**0,0274**	−0.0500	**0,0122**	−0.0484	**0,0029**
Mannose-6-P	−0.0601	−0,0601	−0.0500	**0,0274**	−0.0500	**0,0122**	−0.0484	**0,0029**
Trehalose-6-P	−0.1312	−0,1312	−0.1111	**0,0426**	−0.1111	**0,0122**	−0.1075	−0,0058
ATP	−0.2223	−0,2223	−0.2186	−0,1948	−0.2186	−0,1956	−0.2007	−0,1804
Oxaloacetate	−0.0191	−0,0191	−0.0194	−0,0274	−0.0194	−0,0214	−0.0134	−0,0145
Fumarate	−0.0109	−0,0109	−0.0111	−0,0152	−0.0111	−0,0122	−0.0080	−0.0087
Succinate	−0.0027	−0,0027	−0.0027	−0,0030	−0.0027	−0,0030	−0.0027	−0,0029

G1, G2, G3 and G4, represent the phenotypic phases associated with definite nutrient conditions. Boldface shadow prices represent harmful intermediate metabolites for Mtb. Wild-type (WT); *pfkA*-mutant (Δ*pfkA*)

The black point in G2 represents a bacterium in a necrotic granuloma in the lower part of the lungs, which will acquire appropriate environmental conditions to reactivate from quiescence by consuming available glucose during hypoxia. This phase is identified as a futile phase as defined elsewhere [34]; in this phase, glucose has a partial inhibitory effect towards the growth of bacteria in a broad range of values (from 0.09 to 0.8 mmol/gDW/h) (Fig. 2b). The shadow price for glucose is positive for the Mtb-mutant (Table 1). Likewise, sugar-phosphate intermediates such as glucose-6-phosphate, fructose-6-phosphate, ribose-5-P, xylulose-5-P, mannose-1-P, mannose-6-P, and trehalose-6-P have positive shadow prices, confirming the inhibitory effect of these intermediates on biomass growth. In this G2 phase, the model predicts a harmful inhibitory effect of low oxygen and high glucose uptake rates on biomass growth, which is in agreement with the experimental results obtained by Phong [49]. In contrast, metabolites in wild-type Mtb have negative shadow prices (in all phases) as a trace of positive contribution to Mtb growth.

Phong and co-workers experimentally characterized the behavior of an Mtb *pfkA*-mutant growing on a specific medium – the Dubos medium – under aerobic and hypoxic conditions [49]. They found that if the medium is supplemented with glucose, the *pfkA*-mutant multiply efficiently prior to oxygen depletion. After the 6th day, when oxygen was low, the *pfkA-*mutant exhibited a significant growth inhibition; in contrast, when glucose addition was omitted, the *pfkA-*mutant survived. This phenomenon was explained by the accumulation of toxic intermediates like glucose-6-phosphate and fructose-6-phosphate.

These experimental results demonstrate that in the presence of exogenous glucose, the absence of PFKA activity leads to the accumulation of toxic metabolic intermediates (glucose-6-phosphate and fructose-6 phosphate) in hypoxic non-replicating mycobacteria, as we observed in our simulations (Fig. 2b), and the positive values of shadow prices for sugar-phosphate intermediates (Table 1).

The black point in G3 represents the bacterium in a replicative state inside an early vascularized solid granuloma (Fig. 2c). Here, it is believed that Mtb has access to glucose and oxygen, as it was demonstrated by Guirado and colleagues [50]. The authors found increased transcription of glycolysis genes (*pfkA* and *ppgK*) during Mtb growth within early stage in-vitro granulomas from individuals. The black point in G4 denote a hypothetic scenario of high oxygen consumption; oxygen consumption rate in aerobic *Mycobacterium bovis* BCG cultures was calculated to be 0.98 mmol/gDW/h [19, 51]. These phenotypic phases are also characterized mostly as futile zones (Fig. 2b). Shadow prices of glucose, glucose-6-phosphate, fructose-6-phosphate, mannose-1-phosphate and mannose-6-phosphate for the *pfkA*-mutant have positive values and therefore an adverse effect on biomass growth rate; in G3 and G4, for these sugar-phosphates, shadow prices near zero were observed (0.0122 and 0.0029, respectively) showing a weak inhibition of bacterial growth. In contrast, negative shadow prices are attained for ribose-5-phosphate and xylulose-5-phosphate in G4, indicating the utilization of the pentose phosphate pathway for promoting bacterial growth. Shadow prices for ATP, oxaloacetate, fumarate and succinate are negative in all phases, which suggests that oxidative phosphorylation and TCA cycle are active and promote the survival of Mtb. Similarly, it can be seen that oxygen is important in controlling phenotypic variability in all phases (i.e. negative shadow prices are obtained, see Table 1).

### A metabolic exploration of the *pfkA*-mutant suggests a strong Mtb susceptibility to hypoxia at high glucose levels

In the PhPP, a line of optimality (LO) was exhibited (Fig. 2b), which lies on the boundary between G1 and G2, G3 and G4, and defines the optimal ratio of glucose and oxygen uptake rates for maximal biomass production. At cellular level, the line of optimality represents a scenario in which, once glucose consumption increases, NAD^+^, NADP^+^, and FAD^+^ are converted into their reduced forms to maintain redox balance, thus demanding more oxygen supply to retrieve oxidized cofactor forms; under this scenario, the maximal growth rate is reached. Our metabolic exploration of the *pfkA*-mutant suggests a strong Mtb susceptibility to hypoxia at high glucose levels; the cause of this cell-growth inhibition by the accumulation of sugar-phosphate is unknown, even in *E. coli* [52–54]. Research on this topic has been rather scarce for Mtb.

**Fig. 3:**
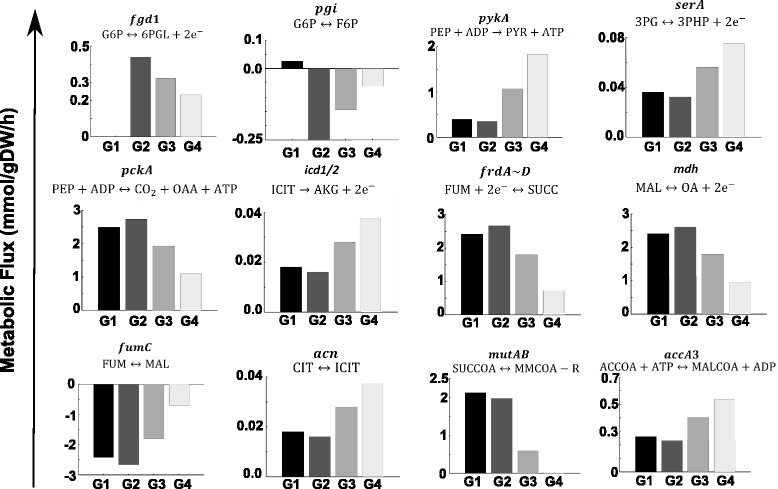
Flux distributions at key enzymes in the *pfkA-*mutant during shifts in glucose and oxygen concentration. Negative and positive fluxes represent backward and forward sense of the reaction. ***pgi***: glucose-6-phosphate isomerase, ***serA***: phosphoglycerate dehydrogenase, ***pckA***: PEP carboxykinase, ***icd1/2***: isocitrate dehydrogenase, ***frdA~D***: fumarate reductase, ***mdh***: malate dehydrogenase, ***fumC***: fumarase, ***acn***: aconitase, ***mutAB***: methylmalonyl-CoA mutase, ***accA3***: acetyl-CoA carboxylase, ***fgd1***: glucose-6-phosphate dehydrogenase, ***pykA***: pyruvate kinase

For explaining the in silico observed toxicity phenotype (Fig. 2b) in the *pfkA-*mutant strain, we used the improved GEM to analyze flux distribution through glycolysis, TCA cycle, pentose phosphate pathway, redox, and energy metabolism in all phases (Additional file 5). During oxygen depletion in solid granulomas (oxygen uptake decreases from G4 to G2; black points), the flux through the glycolytic enzyme glucose-6-phosphate isomerase (*pgi)* increases towards glucose-6-phosphate in the gluconeogenesis sense (Fig. 3), whereas pyruvate kinase (*pykA*) showed a flux decline behavior (Fig. 3). Fluxes of polyphosphate glucokinase (*ppgK*) and phosphoglycerate mutase (*pgmA*) remained unchanged in the glycolytic direction (Additional file 6). Supplementation of glucose exhibited a weak increase in these fluxes (G1 to G2). Regrettably, little has been known about the activity of glycolytic enzymes during hypoxia in Mtb*,* using glycolytic substrates as carbon sources [55].

Glucose-6-phosphate isomerase (*pgi*) catalyzes the reversible isomerization of glucose-6-phosphate to fructose-6-phosphate. Our results predict an increasing gluconeogenesis activity for this enzyme at low oxygen availability, G1, and G2. The phenotypic phases G2 and G4 are characterized by high glucose-6-phosphate dehydrogenase (*fgd1*) activity, the first reaction towards pentose phosphate pathway (Figs. 1 and 3). Conversely, in G1, this enzyme is not active; therefore, merely the supply of both, fructose-6-phosphate and glyceraldehyde-3-phosphate, is allowing carbon flux towards the pentose phosphate pathway.

The *pgmA* enzyme catalyzes the reversible reaction of D-3-phosphoglycerate to 2-phosphoglycerate. It is known that D-3-phosphoglycerate is a precursor of amino acids such as L-serine, glycine, and cysteine. The D-3-phosphoglycerate dehydrogenase (*serA*) catalyzes (in many bacteria, including Mtb*)* the first reaction in the pathway of L-serine biosynthesis by converting D-3-phosphoglycerate to hydroxypyruvic acid phosphate utilizing NAD^+^ as a cofactor (Dey et al., 2005). Our results indicate that while the flux through *pgmA* was unchanged during oxygen depletion (G2), in *serA,* there is a flux decline (Fig. 3). Certainly, under low oxygen availability, the NAD^+^ pool is depleted which is detrimental for *serA* activity.

Flux through phosphoenolpyruvate carboxykinase (*pckA*) was strongly favored in the TCA cycle (Fig. 3). Though *pckA* is primarily associated with gluconeogenesis (the reaction thermodynamics and concentration of related metabolites favors the oxaloacetate (OAA) to phosphoenolpyruvate (PEP) reaction for sugar backbone building), in Mtb during hypoxia, this enzyme has demonstrated an anaplerotic function in favor of OAA production [56]. We observed that the flux at *pckA* increased in the OAA direction with glucose uptake increments and oxygen depletion (G1 to G2 and G4 to G2) (Fig. 3).

In the TCA cycle, the fluxes through aconitase (*acn*) and isocitrate dehydrogenase (*icd1/2*) decrease noticeably at low oxygen conditions; yet, malate dehydrogenase (*mdh*), fumarase (*fumC*), and fumarate reductase (*frdA*~*D*) fluxes (absolute values), increase under oxygen depletion (G4 to G2) (Fig. 3). Glucose uptake increment favors this effect (G1 to G2). The observed flux distribution supports the reductive TCA flux rather than the oxidative one. The large pool of OAA produced by phosphoenolpyruvate carboxykinase (*pckA*) also favors this reductive activity (Fig. 3).

Regarding flux distribution through redox and energy metabolism, a detrimental effect was observed under oxygen depletion; still, flux values are favored as glucose uptake increases. These results are comparable to available literature, e.g., Watanabe and colleagues, where a 2.3-fold increase in intracellular ATP levels and an almost 70-fold increase in the ratio of NADH/NAD^+^ were found [57].

Mtb has various dehydrogenases to fuel the electron transport chain with electron donors NADH, NADPH, and FADH_2_, during oxidation of any carbon source (Fig. 1b). NADH oxidation is carried out by NADH menaquinone oxidoreductases; these enzymes transfer electrons from NADH to menaquinone producing menaquinol, which conserves energy by translocation of protons across the membrane to generate proton motive force (PMF), using F1Fo-ATP synthase. Likewise, menaquinone pool is restored by dioxygen reduction (menaquinol oxidation) in the terminal oxidases, *aa*_3_-type cytochrome *c* oxidase, and cytochrome *bd*-type menaquinol oxidase, which is coupled to the generation of proton motive force [58]. In the absence of oxygen, dioxygen reduction by terminal oxidases is inhibited. This situation entails a decreased activity of NADH dehydrogenase, which leads to poor translocation of protons and a low ATP generation. During this hypoxia phenomenon, Mtb struggles to survive by producing alternative electron acceptors (menaquinone) by fumarate reductase that results in succinate production until replenishing the redox balance, and respiration continues [59, 60]. A similar pathway utilization pattern for *cydD*, *nuoA ~ N* and *atpFH*, at low oxygen uptake rates, was anticipated by our *pfkA*-mutant Mtb metabolic model, and their fluxes decline at low oxygen availability (Additional file 6).

### Metabolic flux distribution provides insights about methyl-branched lipid biosynthesis

The predicted growth rate inhibition under hypoxia and increments of glucose supply may be further described. While fluxes for glycolytic intermediates such as glucose-6-phosphate and fructose-6-phosphate are favored at low oxygen and high glucose uptake rates, fluxes towards the glycogen precursor glucose-1-phosphate and cell wall components derived from fructose-6-phosphate, were rather deprived; in contrast, substantial carbon flux is addressed towards the succinate pool whose accumulation might have contributed to the synthesis of methyl-branched lipids by means of the direct action of methyl malonyl-CoA mutase (*mutAB*) on succinyl-CoA (Fig. 3). Yet, flux towards malonyl-CoA by the enzyme Acetyl-CoA carboxylase (*accA3*) was disfavored which endorses growth inhibition (G2) caused by deprived formation of fatty acids and lipids, as essential components of the Mtb cellular wall (Fig. 3).

Similar results were observed in the synthesis of the amino acid precursors D-3-phosphoglycerate, phosphoenolpyruvate, and pyruvate; accordingly, fluxes involved in the synthesis of the aminoacids L-serine (*serB2*), glycine (*glyA*), tyrosine (*tat*), tryptophan (*trpA*), alanine (*alaT*), isoleucine (*ilvE*), and lysine (*lysA*) were deprived in phase G2 of the PhPP (Additional file 7). Besides, flux towards α-ketoglutarate was very low in G2, thus restraining the availability of critical L-glutamate, arginine, and glutamine for biomass synthesis.

Interestingly, our *in silico* results are in good agreement with experimental reports showing that *pfkA*-mutant adapts to hypoxia inside a granuloma. The metabolic flux analysis showed that, under hypoxic conditions, Mtb slows and redirects its TCA cycle intermediates to increase production of succinate while sustaining the menaquinone pool and ATP synthesis in response to oxygen limitation as it has been reported by Watanabe [57]. Additionally, glucose-6-phosphate and fructose-6-phosphate accumulation enlarges this effect (due to glucose supply increment), producing fewer amounts of biomass precursors, thus resulting in a marked inhibition of growth.

Although there are not enough studies revealing the role of *pfkA*-mutant in vivo*,* our simulations predict that the lethality effect of *pfkA-*mutant might not be prolonged in Mtb subpopulations growing in caseous granulomas. In that environment, Mtb is adapting its metabolism to a non-replicative state; probably, the majority of the bacterial population is consuming lipids as a carbon source, so gluconeogenesis rather than glycolysis is active [50]. In this scenario, a hypothetic drug inhibition of *pfkA* could be useful only during the initial states of the solid necrotic granuloma, or during reactivation, where Mtb can access glycolytic substrates, killing the bacterium just during the transition from replicative to non-replicative state or vice versa.

### Case study II: In silico analysis of the metabolic adaptation of an *icl*-mutant of *Mycobacterium tuberculosis* to changes in oxygen and fatty acid uptake rates.

Isocitrate lyase (ICL1) is a Mtb enzyme that has both isocitrate (ICL) and methylisocitrate lyase (MCL) activities; it is codified by two essential genes for metabolism of even-chain fatty acids [61, 62], *icl1*(Rv0467) and *icl2* (Rv1915/Rv1916), which is divided in the genome of Mtb-H37Rv into two individually expressed modules, Rv1915 and Rv1916 (**aceAa** and **aceAb**). In this study, we mimicked the metabolic adaptation of *icl1* and *icl2* mutants (*icl*-mutant) when fatty acid and oxygen availabilities are changed, using in silico simulations; our extended GEM was also used for this analysis. The model associates a total of 50 pathways including β-oxidation of fatty acids, odd numbered acyl chain fatty acids and of unsaturated fatty acids, that are essential for assimilation of host fatty acids as a carbon source, during Mtb infection [63]. The in silico *icl*-mutant strain lacked the ICL reaction (isocitrate → succinate + glyoxylate) and the MCL reaction (methylisocitrate → succinate) (Fig. 1a). For modeling purposes, we chose propionate as a representative odd-chain fatty acid to be evaluated. In our *icl*-mutant model, propionate is metabolized mainly via beta-oxidation pathway.

**Fig. 4:**
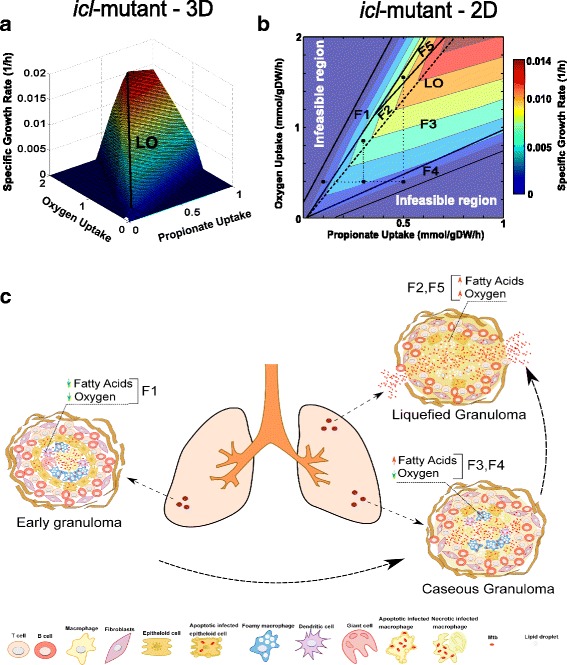
Metabolic adaptation of the *icl*-mutant of Mtb during shifts in propionate and oxygen concentration. Phases in the phenotypic phase plane are associated with hypothetical scenarios of nutrient availability inside granulomas. Black dots correspond to the substrate uptake rates used to simulate the metabolic flux distribution characterizing a given phase. The dotted lines depict constant substrate uptake rates kept between one phenotype to another. **a** 3D top view of the PhPP diagram while optimizing for growth. **b** 2D top view of PhPP highlighting phases and isoclines. **c** Types of granulomas and nutritional environments experienced during Mtb infection

PhPP for the *icl-*mutant is represented in a three-dimensional graph for identifying changes in the slopes of the Phenotypic Planes (Fig. 4a). Phase boundaries, as well as the line of optimality, are shown in Fig. 4b. Each phase represents a distinct metabolic pathway utilization pattern in the Mtb-mutant when the availability of propionate and oxygen are varied. All phases are characterized by positive slopes of the isoclines (Fig. 4b). Therefore, either propionate or oxygen inhibits growth in the *icl-*mutant, in a specific phase. Those phases are considered as futile phases [34]. PhPPs of the wild-type Mtb are shown in Additional file 8. The black point in F1 (similar to G1 in the Case Study I), mimics Mtb inside an early solid granuloma, with poor vascularization, localized at the lower zones of the lung where oxygen could be low (Fig. 4b and c). Accordingly, we are assuming that intracellular Mtb is undergoing a metabolic transition to consumption of lipids where the availability of oxygen and propionate is limited. F3 and F4 depict the intracellular bacterium being phagocytized by foamy macrophages/histiocytes, or extracellular bacteria consuming lipid bodies from death cells inside a caseous granuloma (Fig. 4b and c). Therefore, Mtb is consuming high fatty acid concentrations, while oxygen keeps low levels [43]. Black points in F2 and F5 represent Mtb growing in a broken liquefied granuloma [64] within which the bacterium can consume high levels of fatty acids or lipids in an oxygenated environment.

All phases (F1 to F5) are defined by futile phenotypes, which means that either propionate or oxygen are inhibitory towards supporting biomass growth. Shadow prices for propionate, oxygen and other important metabolites are shown for each phase in Table 2. As observed, oxygen is necessary for biomass growth in F3 and F4, since it has a negative shadow price; yet, in phases, F1, F2, and F5 shadow prices are positive, which means it is inhibitory for growth. In contrast, propionate is important for growth in phases F1, F2, and F5 (negative shadow prices), whereas it is inhibitory for growth in F3 and F4. Indeed, such inhibitory effect (phases F3 and F4), predicted by the in silico analysis, has also been observed experimentally [65]. Lee and co-workers reported a noticeable lethal inhibitory effect of increments of propionate uptake rates on biomass growth rate, thus highlighting the toxic effect of propionyl-CoA accumulation. Although propionyl-CoA toxicity was not directly confirmed (in silico) by positive shadow prices in all explored phenotypes, shadow prices for propionyl-CoA in F3 and F4 were close to zero, indicating that biomass-growth-rate increment was promoted by other metabolites rather than propionyl-CoA.

**Table 2:** Shadow prices for different metabolites in Wild-type and *icl*-mutant strains exposed to different substrate conditions

Metabolite	F1		F2		F3		F4		F5	
	WT	Δ*icl*	WT	Δ*icl*	WT	Δ*icl*	WT	Δ*icl*	WT	Δ*icl*
Propionate	−0.0504	−0.0495	−0.0319	−0.0319	**0.0072**	**0.0072**	**0.0187**	**0.0187**	−0.0458	−0.0458
Oxygen	**0.0101**	**0.0106**	**0.0053**	**0.0053**	−0.0096	−0.0096	−0.0213	−0.0213	**0.0109**	**0.0109**
Propionyl-CoA	−0.0571	−0.0566	−0.0425	−0.0425	−0.0120	−0.0120	−0.0026	−0.0026	−0.0549	−0.0549
Acetyl-CoA	−0.0336	−0.0318	−0.0266	−0.0266	−0.0120	−0.0120	−0.0133	−0.0133	−0.0329	−0.0329
S-Methylmalonyl-CoA	−0.0604	−0.0601	−0.0478	−0.0478	−0.0217	−0.0217	−0.0133	−0.0133	−0.0567	−0.0567
NADH	−0.0033	−0.0071	0	−0.0053	0	0	0	**0.0053**	−0.0018	−0.0055
Succinyl-CoA	−0.0604	−0.0601	−0.0478	−0.0478	−0.0217	−0.0217	−0.0133	−0.0133	−0.0567	−0.0567
2-Methylcitrate	−0.1007	−0.0990	−0.0744	−0.0744	−0.0241	−0.0241	−0.0160	−0.0160	−0.0897	−0.0897
2-Methyl-isocitrate	−0.1007	−0.0990	−0.0744	−0.0744	−0.0241	−0.0241	−0.0160	−0.0160	−0.0897	−0.0897
Succinate	−0.0571	−0.0566	−0.0425	−0.0425	−0.0120	−0.0120	−0.0026	−0.0026	−0.0513	−0.0513
Fumarate	−0.0504	−0.0486	−0.0372	−0.0359	−0.0120	−0.0120	−0.0080	−0.0080	−0.0439	−0.0439
Glyoxylate	−0.0168	−0.0177	−0.0106	−0.0106	−0.0024	0	−0.0133	0	−0.0128	−0.0128
Malate	−0.0504	−0.0495	−0.0372	−0.0372	−0.0121	−0.0121	−0.0080	−0.0080	−0.0439	−0.0439
FADH2	−0.0067	−0.0071	−0.0053	−0.0053	0	0	**0.0053**	**0.0053**	−0.0073	−0.0073

F1, F2, F3, F4 and F5, represent the phenotypic phases associated with specific nutrient conditions. Boldface shadow prices represent harmful intermediate metabolites for Mtb. Wild-type (WT); *pfkA*-mutant (Δ*pfkA*)

**Fig. 5:**
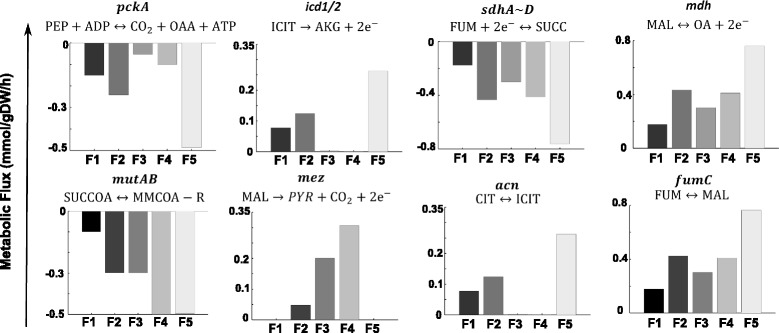
Flux distributions at key enzymes in the *icl-*mutant metabolism during shifts of propionate and oxygen. Negative and positive fluxes represent backward and forward sense of the reaction. ***pckA***: PEP carboxykinase, ***icd1/2***: isocitrate dehydrogenase, ***sdhA~D***: succinate dehydrogenase, ***mdh***: malate dehydrogenase, ***fumC***: fumarase, ***acn***: aconitase, ***mutAB***: methylmalonyl-CoA mutase, ***mez***: malic enzyme

The role of Vitamin B_12_ in Mtb pathogenesis remains poorly understood. Currently, it has been demonstrated an enzymatic-dependent role of Vitamin B_12_ for the activity of methionine synthase (MetH) and methylmalonyl-CoA mutase (mutAB), during methionine biosynthesis and propionate metabolism (methylmalonyl pathway) [65–68]. Our simulations with the in silico *icl*-mutant predict an active methylmalonyl pathway, which corresponds to the in-vivo scenario of available vitamin B_12_. Spontaneous in silico activation of the methylmalonyl pathway occurs because our GEM does not take into account vitamin B_12_ as a cofactor for methylmalonyl-CoA mutase (mutAB). Consequently, simulations of the *icl*-mutant metabolism (where glyoxylate shunt and methylcitrate cycle are disrupted) show propionate catabolism by activation of methylmalonyl-CoA mutase (mutAB) (Fig. 5)

Pathway utilization analysis was carried out for the five representative points of the identified phenotypes in the PhPP for both, *icl*-mutant and wild-type (Additional file 9). The most relevant results for the *icl*-mutant are presented in Fig. 5; complementary flux distribution analysis for the main enzymes are shown in Additional file 10. For all phenotypes, a similar pathway utilization pattern, which is consistent with experimental observations, was found [66]. We also saw that when our Mtb model relies on high concentrations of propionate, propionyl-CoA ligase catalyzes the formation of propionyl-CoA, whose toxicity on the *icl*-mutant is only prevented by the methylmalonyl pathway; methylmalonyl-CoA not only goes to constitute methyl-branched lipids but also enters the TCA cycle, after conversion to succinyl-CoA by methylmalonyl-CoA mutase (mutAB). From the TCA cycle, the glycolytic substrates could be replenished by gluconeogenesis, a reaction mediated by phosphoenolpyruvate carboxykinase (*pckA*). Replenishing of pyruvate takes place by the action of the malic enzyme (*mez*) (Fig. 5).

The *icl-*mutant phenotypes F1, F3, and F4, were analyzed at a fixed oxygen uptake rate (0.4 mmol gDW^−1^ h^−1^) with increasing values of propionate uptake rates (0.1, 0.3 and 0.5 mmol gDW^−1^ h^−1^). In F1, propionate is essential for growth, but in F3 and F4 (larger propionate uptake), it is inhibitory. Differences in metabolic flux distribution towards amino acid biosynthesis were also observed. From F1 to F2 there is an increase in fluxes towards phenylalanine, histidine, valine, alanine, glycine, lysine, threonine and methionine (Additional file 11), which is consistent with the growth rate boost between the two points examined within these two phases. In contrast, a reduction in the flux towards these amino acids occurred in F3 and F4. Particularly, in these two “inhibitory” or “toxic” phenotypes (caseous granuloma), we found that metabolic fluxes through the TCA cycle, i.e., aconitase (*acn*), isocitrate dehydrogenase (*icd1/2*), succinate dehydrogenase (*sdhA~ D*, fumarase (*fumC*) and malate dehydrogenase (*mdh*), were disfavored in comparison with F2 and F5 (broken liquefied granuloma) (Fig. 5).

### High levels of propionate uptake negatively affect both redox metabolism and vital metabolic pathways in Mtb *icl-*mutants

The needy activity of the TCA cycle in the *icl-*mutant was also predicted by small negative values for the metabolic-intermediate shadow prices (isocitrate, succinate, succinyl-CoA, malate, and fumarate) in F3 and F4 (Table 2). In the *icl*-mutant, the depletion of intermediates in this pathway might produce a redox imbalance (NADH/NAD^+^, NADPH/NADP^+^, and FADH_2_/FAD) for Mtb metabolism at low oxygen uptake rates. Shadow prices for NADH/NADPH and FADH2 in our *icl*-mutant Mtb model were either zero or positive at phenotypes F3 and F4, respectively. Although the magnitude of shadow prices for the wild-type Mtb is very similar to the *icl-*mutant, a slight difference is found in the values of the shadow prices of metabolites in phase F1, indicating a small positive or negative contribution of those metabolites to Mtb’s growth. The NADH’s shadow prices were zero or negative for all phases in the wild-type strain, which is an indication of a slight improvement of NADH/NAD^+^ balance (in the wild type) in comparison with the *icl-*mutant. A possible explanation for this mild improvement in redox balance is the activation of carbon flux by the isocitrate lyase (ICL) and methylisocitrate lyase (MCL) enzymes, in addition to the methylmalonyl pathway activation.

Therefore, we argue that high levels of propionate uptake (Phenotypes F3 and F4) negatively affect both redox metabolism (low NAD^+^/NADP^+^/FAD^+^ recovery and ATP synthesis), and vital metabolic pathways as gluconeogenesis, pentose phosphate pathway, synthesis of amino acids, mycolic acids synthesis, and synthesis of methyl-branched lipids (Additional files 10, 11 and Fig. 5). Furthermore, phenotype F4 revealed a high formate hydrogen lyase enzyme *(hycD*) activity (*hycD* catalyzes the production of molecular hydrogen and CO_2_ from formate and atomic hydrogen), caused apparently by an increase of intracellular H^+^ (Additional file 10). Our observations harmonize with the recent findings of Eoh and Rhee [69], who explored the toxic effect of propionate and acetate addition on a Mtb-ICL mutant. Their findings can be summarized as i) depletion of TCA cycle and gluconeogenic pathway intermediates, ii) noncompetitive inhibition of 2-methylcitrate on fructose-1,6-biphosphatase activity, and iii) shifts in NAD/NADH pools, changes in membrane potential and a decrease in the intracellular pH. Also, under aerobic conditions, vitamin B_12_ dependent pathway (methylmalonyl pathway) led selective corrections in TCA cycle activity and membrane potential.

### Inside hypoxic caseous granulomas, a strong lethality of *icl-*mutant seems to be favored in Mtb populations surviving in fatty acids

Although there is not a direct evidence of the effect of hypoxia on the methylmalonyl pathway in vivo, our findings predict that oxygen limitation creates an inability to methylmalonyl pathway to correct TCA cycle activity and membrane potential when the *icl*-mutant consumes fatty acids, unlike to Eoh and Rhee’s work, found under aerobic conditions [69]. In other words, a strong lethality of *icl-*mutant seems to be favored in Mtb populations surviving inside hypoxic caseous granulomas with available fatty acids. This lethality is lost in Mtb growing in broken liquefied granulomas during replicative state, probably because oxygen can restore NAD and NADH pools and therefore membrane potential (F5). Additionally, phenotypes F2 and F5 (analyzed at higher values of oxygen: 0.85 and 1.55 mmol gDW^−1^ h^−1^, respectively) exhibited a noticeable boost in all fluxes towards lipids, amino acids, gluconeogenesis, and pentose phosphate pathway; this is consistent with the increase in Mtb mutant proliferation within broken liquefied granulomas.

### Effect of the consumption of asparagine on the metabolic reprogramming of *pfkA*- and *icl*-mutant strains of Mtb in a caseous granuloma scenario

Nitrogen is an essential molecule for Mtb to produce amino acids, proteins, and nucleotides. Metabolite labeling studies have confirmed that Mtb actively imports amino acids such as alanine, aspartate, asparagine, glutamate and glutamine from the host cytoplasm [70].

**Fig. 6:**
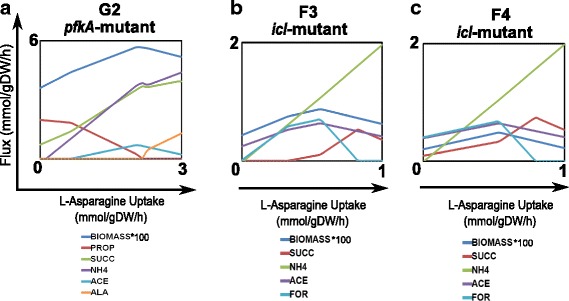
Metabolic adaptation of *icl-* and *pfkA*-mutants under hypoxia and asparagine consumption. **a** Effect of asparagine consumption in the G2 phase of the *pfkA-*mutant. **b** Effect of asparagine consumption in the F3 phase of the *icl-*mutant. **c** Effect of asparagine consumption in the F4 phase of the *icl*-mutant. ACE: acetate, GLU: glutamate, SUCC: succinate, ALA: L-alanine, NH4: ammonium, FOR: formate, PROP: propionate

Metabolic profiling during the infection in guinea pig’s lung granulomas infected with Mtb showed accumulation of acetate, glutamate, and aspartate as the infection progressed [7]. It is believed that aspartate accumulation could be a consequence of the utilization of asparagine from the host. Asparaginases catalyze the hydrolysis of asparagine into aspartate and ammonia to gain resistance to acid stress [71, 72]. Therefore, we decided to explore the metabolic adaptation during consumption of asparagine in the hypoxic phases of *pfkA*- and *icl*-mutants (G2 and F3/F4 respectively), which corresponds to a caseous granuloma scenario. In the metabolic model, this effect was captured by the relaxation of reaction exchange boundaries, thus allowing asparagine consumption at each punctual phase. The consumption range values showed in Fig. 6, were those that allowed a feasible solution of the optimization problem.

Mtb allows asparagine uptake by *AnsP2* (Rv0346c). *AnsA* (Rv1538c) is the asparaginase enzyme involved in asparagine deamination into aspartate, which releases ammonia; subsequent spontaneous protonation produces ammonium. It is believed that it has a major role in resistance to acid stress, both in vitro and in vivo.

Our in silico results indicate that the use of asparagine as nitrogen source presented an equilibrated balance between growth and inhibition at different levels of consumption in both Mtb mutants (Fig. 6). For instance, Mtb growth was slightly favored in G1 and F3/F4 phases when asparagine consumption was below 2 mmol gDW^−1^ h^−1^ and 0.6 mmol gDW^−1^ h^−1^ respectively. In contrast, for consumption rates higher than 2 mmol gDW^−1^ h^−1^ and 0.6 mmol gDW^−1^ h^−1^, a slightly growth inhibition arose. Furthermore, asparagine consumption leads to ammonium secretion in the *pfkA-* and *icl*-mutants (as described by Gouzy and coworkers), high fumarate reductase activity and high secretion of succinate, which might be responsible for the minor growth inhibition by carbon loss (Fig. 6). These results suggest that the reductive TCA cycle is active with increased asparagine uptake rates; yet, under lower asparagine uptake rates, succinate secretion is significantly reduced, and carbon flux is redirected towards acetyl-CoA. This behavior is expected since asparagine deaminase (*ansA*) catalyzes the synthesis of aspartate, which in turns, allows for the synthesis of oxaloacetate using aspartate oxidase (*nadB,* Rv1595) or the reversible aspartate transaminase (*aspBC*, Rv3565, Rv0337c). Flux distributions data derived from the additional asparagine consumption on phases G2 and F3/F4 of Mtb-mutants are included in Additional files 12 and 13, respectively.

In both *pfkA-*mutant and *icl-*mutant, the high levels of oxaloacetate should be reduced by the malate dehydrogenase activity, which augments flux in the reductive TCA cycle direction and might favor succinate production. Although recently, succinate production has been associated with elevated carbon flow through the glyoxylate shunt [59], in the *pfkA-*mutant simulations, the glyoxylate shunt could not carry flux with asparagine consumption. We believe that the use of glycolytic substrates like glucose and glycerol could increase succinate production by fumarate reductase activity during hypoxia, but the use of acetate may favor succinate production through the glyoxylate shunt [59].

Furthermore, asparagine consumption allowed the production of alanine during the hypoxic caseous granuloma of phase G2. In Mtb, one of the genes upregulated in response to hypoxia is *ald* (Rv2780), encoding L-alanine dehydrogenase [73]. This enzyme catalyzes the reversible NAD-dependent interconversion of L-alanine to pyruvate. Under aerobic conditions, L-alanine is used as a nitrogen source, whereas under hypoxic conditions, this reaction allows recuperation of NAD^+^ and alanine to recover redox balance during non-replicative states. The use of glycolytic substrates by the *pfkA*-mutant, when asparagine is used as a nitrogen source, seems to favor L-alanine production.

Acetate was produced and spent when both, *pfkA*-mutant and *icl*-mutant consumed asparagine in the phases G2 and F3/F4. Acetate accumulation has been detected in guinea-pig granulomas infected with Mtb [7]. Though it is believed that most of the detected metabolites in guinea pig granulomas are derived from the host metabolism, Mtb might also produce them. Rücker and coworkers demonstrated that Mtb had evolved a dual role in acetate metabolism. Mtb uses enzymes like phosphate acetyltransferase *(pta)* and acetate kinase (*ackA*), which together can mediate acetate production (when Mtb is consuming pyruvate or fatty acids) to release an excess of carbon units and resumption of acetate as a carbon substrate depending on the available substrates for Mtb [74].

Nonetheless there is no evidence of propionate production by Mtb; the metabolic model used predicts it using two enzymes that do not have an annotated function in Mtb but in *E. coli*: methyl-malonyl-CoA decarboxylase (scpB) and succinate CoA transferase (scpC) [75]. Similar findings were obtained from the model, regarding the prediction of acetate production; it was produced by acetoacetyl-CoA transferase (*atoD*), another *E. coli* enzyme. These findings point out the need for a further manual curation of the existing GEMs of Mtb; it will contribute to enhance the prediction of metabolic adaptations under a plethora of external perturbations. Indeed, new experimental evidence is needed to prove acetate production by high asparagine uptake rates, adding new insights about the role of the acetate metabolism in Mtb.

Finally, formate was produced just in the F3/F4 phases of the *icl*-mutant, by the high activity of the enzyme formate-tetrahydrofolate ligase (*fhs1&2*), which catalyzes the interconversion between tetrahydrofolate and 10-Formyltetrahydrofolate, the latter acts as a donor of formyl groups in de novo purine biosynthesis pathway.

Still our simulations do not show considerable inhibition or improvements of growth in the Mtb-mutants. A comparison between metabolic modeling predictions and literature findings allows us to propose a schematic representation of carbon flux distributions during the metabolic adaptation of the *pfkA*- and *icl*-mutants, when asparagine is used as a nitrogen source, in an environment such as the one of a hypoxic caseous granuloma (Additional files 14 and 15).

Any of the phases analyzed here showed zero growth when asparagine was used as an additional nitrogen source in the explored ranges. It simply points out the success of the lack of a catabolite repression mechanism for the survival of Mtb inside a granuloma with “infinite” availability of carbon and nitrogen sources [76].

## Conclusions

Recent findings in humans and the non-human primate model of Mtb infection have indicated that host-pathogen interactions within lesions are a dynamic process, driven by subtle and local differences in signaling pathways, resulting in diverging trajectories of lesions within a single host [39]. These findings suggest that Mtb metabolism is highly adaptable, which enables the microorganism to survive for long periods, even under demanding host environments. The study of the lethality of Mtb mutants and the optimal use of its metabolic pathways during exigent nutritional environments, in the context of human granulomas, was the main facet analyzed in this work. Our in silico analysis confirmed some particular hypothesis generated in vivo and in-vitro, related to the use of glycolysis, TCA cycle, and propionate metabolism.

A harmful effect arises in a *pfkA-*mutant only during the adaptation to hypoxia in a solid granuloma with available glucose. Otherwise, the bacterium was capable of sustaining growth. In this hypoxia adaptation, we found that Mtb slows and redirects its TCA cycle to increase production of succinate by fumarate reductase while sustaining the menaquinone pool and ATP synthesis. Therefore, inhibition of *pfkA* would be effective only during the initial states of the solid necrotic granuloma, or during reactivation, where Mtb has access to glycolytic substrates; otherwise, in further stages of the disease, a hypothetical drug inhibition would not be effective against Mtb.

In addition, using shadow price calculations, we predict, for the first time, the appearance of toxic sugar phosphate intermediates (such as glucose-6-phosphate and fructose-6-phosphate) as glucose uptake increases in the *pfkA*-mutant (a phenotype found experimentally for the Mtb *pfkA*-mutant under the same conditions). This finding shows the potential of genome-scale modeling and shadow pricing for the identification and study of toxic metabolic intermediates.

The lethality of an *icl*-mutant was high during the transition from solid to necrotic granuloma, which Mtb population can access to large quantities of fatty acids and hypoxia but not in a liquefied granuloma where oxygen is available. We confirmed the importance of the activity of methyl malonyl pathway and acetate consumption for the detoxification of propionyl-CoA during high propionate uptake rates. We also found a decrease in TCA cycle intermediates that produce a redox imbalance during hypoxia conditions and low ATP synthesis, but methyl malonyl pathway could revert the effect and sustain growth under aerobic conditions, in a liquefied granuloma. Thus, a drug inhibition of *icl*-mutant seems to be favored in Mtb populations surviving inside hypoxic caseous granulomas. Although previous studies in mouse model showed that a deletion of both *icl* genes causes a profound attenuation during both the acute and chronic phases of mouse infections, this model does not form clear hypoxic caseous granulomas as in humans [77]; therefore, this conclusion might require further validation. Moreover, there is evidence that ICLs have important roles in adaptation to hypoxia and antibiotic tolerance by mechanisms such an antioxidant defense that are independent of fatty acid metabolism [59, 78].

Furthermore, in silico results showed that asparagine consumption promotes production of succinate, and alanine for restoring redox balance in both Mtb mutants, as reported in the literature. Besides, acetate resumption seems to have an important role in Mtb metabolic reprogramming at changes in carbon and nitrogen sources.

The use of genome-scale modeling and shadow price analysis might help to accelerate our metabolic understanding about the essentiality of Mtb gene knockouts, to generate hypothesis about possible drug targets that could be lethal during all the stages of TB disease, and to prioritize resource-intensive experimental work in the development of new anti-TB drugs.

Although in this study we explored the metabolic reprogramming of only two Mtb knockouts, future efforts will be made to provide a web server for displaying the in silico metabolic phenotypes reached by a large number of mutants (known metabolic-based drug targets) under diverse nutritional conditions. This tool will accelerate the study of metabolic reprogramming of Mtb mutants under a wide range of intracellular constraints.

## Additional files

Additional file 1: Flowchart of the methodology adopted in this in silico study. (PDF 41 kb)

Additional file 2: New reactions included into the iOSDD890 model. (XLSX 24 kb)

Additional file 3: Improved genome-scale model of Mtb from iOSDD890. (XML 2218 kb)

Additional file 4: Phenotypic phase planes of the Wild-Type Mtb during shifts of glucose and oxygen. (**a**) 3D top view of the PhPP. (**b**) 2D top view of the PhPP highlighting isoclines with negative slopes. (PDF 457 kb)

Additional file 5: Flux values and shadow prices for all the explored nutrient conditions of the *pfkA*-mutant and Wildt-type. Shadow prices were obtained by dual solution of the FBA while fluxes were obtained by minimization of enzyme usage. (XLSX 287 kb)

Additional file 6: Carbon flux distributions depicting other enzymes for the *pfkA*-mutant in phases G1, G2, G3, and G4. ***ppgK***: polyphosphate glucokinase, ***pgmA***: phosphoglucomutase, ***pgk***: phosphoglycerate kinase, ***atpFH***: ATP synthase, ***nuoA∼N***: NADH dehydrogenase, ***cydD***: cytochrome c oxido-reductase. (PDF 1119 kb)

Additional file 7: Flux towards amino acid biosynthesis in phases G1, G2, G3 and G4 of the *pfkA*-mutant. ***arcA***: arginine deiminase, ***lysA***: diaminopimelate decarboxylase, ***ilvE***: branched-chain-amino-acid aminotransferase, ***trpA***: tryptophan synthase, ***tat***: tyrosine aminotransferase, ***glyA***: serine hydroxymethyltransferase, ***alaT***: alanine aminotransferase, ***aspB***: aspartate aminotransferase, ***gdh***: glutamate dehydrogenase, ***serB2***: phosphoserine phosphatase, ***proC***: pyrroline-5-carboxylate reductase. (PDF 384 kb)

Additional file 8: Phenotypic phase planes of the Wild-Type Mtb during shifts of propionate and oxygen. (**a**) 3D top view of the PhPP. (**b**) 2D top view of the PhPP highlighting isoclines. (PDF 623 kb)

Additional file 9: Flux values and shadow prices for all the explored conditions of the *icl*-mutant and Wildt-type strains. Shadow prices were obtained by dual solution of the FBA while fluxes were obtained by minimization of enzyme usage. (XLSX 362 kb)

Additional file 10: Carbon flux distributions depicting additional enzymes for the *icl*-mutant in phases F1, F2, F3, F4, and F5. (PDF 200 kb)

Additional file 11: Flux towards amino acid biosynthesis in phases F1, F2, F3, F4 and F5 of the *pfkA*-mutant. (PDF 265 kb)

Additional file 12: Flux values for the phase G2 of the *pfkA*-mutant during asparagine consumption. (XLS 287 kb)

Additional file 13: Flux values for the phases F3 and F4 of the *icl*-mutant during asparagine consumption. (XLS 1716 kb)

Additional file 14: Model scheme of fluxes during the metabolic adaptation the *pfkA*-mutant in a caseous granuloma (G2) during the consumption of asparagine. Blue boxes represent up taken and produced metabolites. (PDF 52 kb)

Additional file 15: Model scheme of fluxes during the metabolic adaptation of the *icl*-mutant in a caseous granuloma (F3/F4), during the consumption of asparagine. Blue boxes represent up taken and produced metabolites. (PDF 58 kb)

## Abbreviations

CCM: Central carbon metabolism
FBA: Flux balance analysis
GEM: Genome-scale model
ICL: Isocitrate lyase
LO: Line of optimality
LP: Linear Programming
LTBI: Latent tuberculosis infection
MC3: The Model and Constraint Consistency Checker
MCL: Methylcitrate lyase
MDR: Multidrug-resistant
Mtb: *Mycobacterium tuberculosis*
OAA: Oxaloacetate
PEP: Phosphoenolpyruvate
PFK: Phosphofructokinase
PhPP: Phenotypic phase plane
PMF: Proton motive force
TB: Tuberculosis
TCA: Tricarboxylic acid
WHO: World Health Organization
WT: Wild type
XDR: Extensively drug resistant

## References

[1] World Health Organization (WHO). Global tuberculosis report. Geneva: WHO, 2016.

[2] Gengenbacher M, Kaufmann SH (2012). *Mycobacterium tuberculosis*: success through dormancy. FEMS Microbiol Rev.

[3] Warner DF (2015). *Mycobacterium tuberculosis* metabolism. Cold Spring Harb Perspect Med.

[4] Daniel J, Maamar H, Deb C, Sirakova TD, Kolattukudy PE (2011). *Mycobacterium tuberculosis* uses host triacylglycerol to accumulate lipid droplets and acquires a dormancy-like phenotype in lipid-loaded macrophages. PLoS Pathog.

[5] Reece ST, Kaufmann SH (2012). Floating between the poles of pathology and protection: can we pin down the granuloma in tuberculosis?. Curr Opin Microbiol.

[6] Russell DG, Cardona P-J, Kim M-J, Allain S, Altare F (2009). Foamy macrophages and the progression of the human tuberculosis granuloma. Nat Immunol.

[7] Somashekar B, Amin AG, Rithner CD, Troudt J, Basaraba R, Izzo A (2011). Metabolic profiling of lung granuloma in *Mycobacterium tuberculosis* infected guinea pigs: ex vivo 1H magic angle spinning NMR studies. J Proteome Res.

[8] Zimmermann M, Kogadeeva M, Gengenbacher M, McEwen G, Mollenkopf H-J, Zamboni N (2017). Integration of metabolomics and transcriptomics reveals a complex diet of *Mycobacterium tuberculosis* during early macrophage infection. mSystems.

[9] Puckett S, Trujillo C, Eoh H, Marrero J, Spencer J, Jackson M (2014). Inactivation of fructose-1, 6-bisphosphate aldolase prevents optimal co-catabolism of glycolytic and gluconeogenic carbon substrates in *Mycobacterium tuberculosis*. PLoS Pathog.

[10] Trujillo C, Blumenthal A, Marrero J, Rhee KY, Schnappinger D, Ehrt S (2014). Triosephosphate isomerase is dispensable in vitro yet essential for *Mycobacterium tuberculosis* to establish infection. MBio.

[11] Marrero J, Trujillo C, Rhee KY, Ehrt S (2013). Glucose phosphorylation is required for *Mycobacterium tuberculosis* persistence in mice. PLoS Pathog.

[12] Puckett S, Trujillo C, Wang Z, Eoh H, Ioerger TR, Krieger I (2017). Glyoxylate detoxification is an essential function of malate synthase required for carbon assimilation in *Mycobacterium tuberculosis*. Proc Natl Acad Sci.

[13] Ruecker N, Jansen R, Trujillo C, Puckett S, Jayachandran P, Piroli GG, et al. Fumarase deficiency causes protein and metabolite succination and intoxicates *Mycobacterium tuberculosis*. Cell Chem Biol. 2017;24:306–15. 10.1016/j.chembiol.2017.01.005 PMC5357164 28219662

[14] Hasan MR, Rahman M, Jaques S, Purwantini E, Daniels L (2010). Glucose 6-phosphate accumulation in mycobacteria implications for a novel F420-dependent anti-oxidant defense system. J Biol Chem.

[15] Ganapathy U, Marrero J, Calhoun S, Eoh H, De Carvalho LPS, Rhee K (2015). Two enzymes with redundant fructose bisphosphatase activity sustain gluconeogenesis and virulence in *Mycobacterium tuberculosis*. Nat Commun.

[16] Noy T, Vergnolle O, Hartman TE, Rhee KY, Jacobs WR, Berney M (2016). Central role of pyruvate kinase in carbon co-catabolism of *Mycobacterium tuberculosis*. J Biol Chem.

[17] Bordbar A, Monk JM, King ZA, Palsson BO (2014). Constraint-based models predict metabolic and associated cellular functions. Nat Rev Genet.

[18] O’Brien EJ, Monk JM, Palsson BO (2015). Using genome-scale models to predict biological capabilities. Cell.

[19] Jamshidi N, Palsson BØ. Investigating the metabolic capabilities of *Mycobacterium tuberculosis* H37Rv using the in silico strain iNJ661 and proposing alternative drug targets. BMC Syst Biol. 2007;1:26. 10.1186/1752-0509-1-26 PMC1925256 17555602

[20] Beste DJ, Hooper T, Stewart G, Bonde B, Avignone-Rossa C, Bushell ME (2007). GSMN-TB: a web-based genome-scale network model of *Mycobacterium tuberculosis* metabolism. Genome Biol.

[21] Chindelevitch L, Stanley S, Hung D, Regev A, Berger B (2012). MetaMerge: scaling up genome-scale metabolic reconstructions with application to *Mycobacterium tuberculosis*. Genome Biol.

[22] Fang X, Wallqvist A, Reifman J (2012). Modeling phenotypic metabolic adaptations of *Mycobacterium tuberculosis* H37Rv under hypoxia. PLoS Comput Biol.

[23] Lofthouse EK, Wheeler PR, Beste DJ, Khatri BL, Wu H, Mendum TA (2013). Systems-based approaches to probing metabolic variation within the *Mycobacterium tuberculosis* complex. PLoS One.

[24] Rienksma RA, Suarez-Diez M, Spina L, Schaap PJ (2014). Martins dos Santos VA. Systems-level modeling of mycobacterial metabolism for the identification of new (multi-)drug targets. Semin Immunol.

[25] Garay CD, Dreyfuss JM, Galagan JE (2015). Metabolic modeling predicts metabolite changes in *Mycobacterium tuberculosis*. BMC Syst Biol.

[26] Y-K O, Palsson BO, Park SM, Schilling CH, Mahadevan R. Genome-scale reconstruction of metabolic network in *Bacillus subtilis* based on high-throughput phenotyping and gene essentiality data. J Biol Chem. 2007;282:28791–9. 10.1074/jbc.M703759200 17573341

[27] Joyce AR, Palsson BØ. Predicting gene essentiality using genome-scale in silico models. Microb Gene Essentiality Protoc Bioinforma. 2008:433–57. 10.1007/978-1-59745-321-9_30 18392986

[28] Fanalysis of the opportunistic pathogen analysis of the opportunistic pathogen *Pseudomonas aeruginosa* PAO1. J Bacteriol. 2008;190:2790–803. 10.1128/JB.01583-07 PMC2293256 18192387

[29] Orth JD, Conrad TM, Na J, Lerman JA, Nam H, Feist AM, et al. A comprehensive genome-scale reconstruction of *Escherichia coli* metabolism—2011. Mol Syst Biol. 2011;7:535. 10.1038/msb.2011.65 PMC3261703 21988831

[30] Vashisht R, Bhat AG, Kushwaha S, Bhardwaj A, Brahmachari SK, Consortium OSDD (2014). Systems level mapping of metabolic complexity in *Mycobacterium tuberculosis* to identify high-value drug targets. J Transl Med.

[31] Yousofshahi M, Ullah E, Stern R, Hassoun S (2013). MC3: a steady-state model and constraint consistency checker for biochemical networks. BMC Syst Biol.

[32] Schuetz R, Kuepfer L, Sauer U. Systematic evaluation of objective functions for predicting intracellular fluxes in *Escherichia coli*. Mol Syst Biol. 2007;3:119. 10.1038/msb4100162 PMC1949037 17625511

[33] Sánchez CEG, Sáez RGT (2014). Comparison and analysis of objective functions in flux balance analysis. Biotechnol Prog.

[34] Edwards JS, Ibarra RU, Palsson BO. In silico predictions of *Escherichia coli* metabolic capabilities are consistent with experimental data. Nat Biotechnol. 2001;19:125–30. 10.1038/84379 11175725

[35] Edwards JS, Ramakrishna R, Palsson BO (2002). Characterizing the metabolic phenotype: a phenotype phase plane analysis. Biotechnol Bioeng.

[36] D’Huys P-J, Lule I, Vercammen D, Anné J, Van Impe JF, Bernaerts K. Genome-scale metabolic flux analysis of *Streptomyces lividans* growing on a complex medium. J Biotechnol. 2012;161:1–13. 10.1016/j.jbiotec.2012.04.010 22641041

[37] Holzhütter H (2004). The principle of flux minimization and its application to estimate stationary fluxes in metabolic networks. Eur J Biochem.

[38] Khatri B, Fielder M, Jones G, Newell W, Abu-Oun M, Wheeler PR. High throughput phenotypic analysis of *Mycobacterium tuberculosis* and *Mycobacterium bovis* strains’ metabolism using biolog phenotype microarrays. PLoS One. 2013;8:e52673. 10.1371/journal.pone.0052673 PMC3542357 23326347

[39] Lenaerts A, Barry CE, Dartois V (2015). Heterogeneity in tuberculosis pathology, microenvironments and therapeutic responses. Immunol Rev.

[40] Alatas F, Alatas O, Metintas M, Ozarslan A, Erginel S, Yildirim H (2004). Vascular endothelial growth factor levels in active pulmonary tuberculosis. CHEST J.

[41] Saita N, Fujiwara N, Yano I, Soejima K, Kobayashi K (2000). Trehalose 6, 6’-dimycolate (cord factor) of *Mycobacterium tuberculosis* induces corneal angiogenesis in rats. Infect Immun.

[42] Aly S, Wagner K, Keller C, Malm S, Malzan A, Brandau S (2006). Oxygen status of lung granulomas in *Mycobacterium tuberculosis*-infected mice. J Pathol.

[43] Kim M, Wainwright HC, Locketz M, Bekker L, Walther GB, Dittrich C (2010). Caseation of human tuberculosis granulomas correlates with elevated host lipid metabolism. EMBO Mol Med.

[44] Deb C, Daniel J, Sirakova TD, Abomoelak B, Dubey VS, Kolattukudy PEA (2006). Novel lipase belonging to the hormone-sensitive lipase family induced under starvation to utilize stored triacylglycerol in *Mycobacterium tuberculosis*. J Biol Chem.

[45] Raju B, Hoshino Y, Belitskaya-Lévy I, Dawson R, Ress S, Gold JA (2008). Gene expression profiles of bronchoalveolar cells in pulmonary TB. Tuberculosis.

[46] Pandey AK, Sassetti CM (2008). Mycobacterial persistence requires the utilization of host cholesterol. Proc Natl Acad Sci.

[47] Garton NJ, Waddell SJ, Sherratt AL, Lee S-M, Smith RJ, Senner C (2008). Cytological and transcript analyses reveal fat and lazy persister-like bacilli in tuberculous sputum. PLoS Med.

[48] Caire-Brändli I, Papadopoulos A, Malaga W, Marais D, Canaan S, Thilo L, et al. Reversible lipid accumulation and associated division arrest of *Mycobacterium avium* in lipoprotein-induced foamy macrophages may resemble key events during latency and reactivation of tuberculosis. Infect Immun. 2014;82:476–90. 10.1128/IAI.01196-13 PMC3911402 24478064

[49] Phong WY, Lin W, Rao SP, Dick T, Alonso S, Pethe K (2013). Characterization of phosphofructokinase activity in *Mycobacterium tuberculosis* reveals that a functional glycolytic carbon flow is necessary to limit the accumulation of toxic metabolic intermediates under hypoxia. PLoS One.

[50] Guirado E, Mbawuike U, Keiser TL, Arcos J, Azad AK, Wang S-H (2015). Characterization of host and microbial determinants in individuals with latent tuberculosis infection using a human granuloma model. MBio.

[51] Van Den Heuvel N, Tiesjema R, Van Hemert P (1981). Optimization measurement of oxygen uptake rate of BCG with the Gilson Oxygraph. Antonie Van Leeuwenhoek.

[52] Kadner RJ, Murphy GP, Stephens CM. Two mechanisms for growth inhibition by elevated transport of sugar phosphates in *Escherichia coli*. J Gen Microbiol. 1992;138:2007–14. 10.1099/00221287-138-10-2007 1479338

[53] Vanderpool CK (2007). Physiological consequences of small RNA-mediated regulation of glucose-phosphate stress. Curr Opin Microbiol.

[54] Richards GR, Patel MV, Lloyd CR, Vanderpool CK. Depletion of glycolytic intermediates plays a key role in glucose-phosphate stress in *Escherichia coli*. J Bacteriol. 2013;195:4816–25. 10.1128/JB.00705-13 PMC3807488 23995640

[55] Rohde KH, Veiga DF, Caldwell S, Balázsi G, Russell DG (2012). Linking the transcriptional profiles and the physiological states of *Mycobacterium tuberculosis* during an extended intracellular infection. PLoS Pathog.

[56] Beste DJ, Bonde B, Hawkins N, Ward JL, Beale MH, Noack S (2011). 13 C metabolic flux analysis identifies an unusual route for pyruvate dissimilation in mycobacteria which requires isocitrate lyase and carbon dioxide fixation. PLoS Pathog.

[57] Watanabe S, Zimmermann M, Goodwin MB, Sauer U, Barry CE, Boshoff HI (2011). Fumarate reductase activity maintains an energized membrane in anaerobic *Mycobacterium tuberculosis*. PLoS Pathog.

[58] Cook GM, Hards K, Vilchèze C, Hartman T, Berney M. Energetics of respiration and oxidative phosphorylation in mycobacteria. In: Hatfull GF Jacobs WR, editors. Molecular Genetics of Mycobacteria, 2nd edition. Washington DC: ASM Press; 2014. p. 389–409. 10.1128/microbiolspec.MGM2-0015-2013 PMC4205543 25346874

[59] Eoh H, Rhee KY (2013). Multifunctional essentiality of succinate metabolism in adaptation to hypoxia in *Mycobacterium tuberculosis*. Proc Natl Acad Sci.

[60] Hartman T, Weinrick B, Vilchèze C, Berney M, Tufariello J, Cook GM (2014). Succinate dehydrogenase is the regulator of respiration in *Mycobacterium tuberculosis*. PLoS Pathog.

[61] Muñoz-Elías EJ, McKinney JD (2005). *Mycobacterium tuberculosis* isocitrate lyases 1 and 2 are jointly required for in vivo growth and virulence. Nat Med.

[62] Griffin JE, Pandey AK, Gilmore SA, Mizrahi V, Mckinney JD, Bertozzi CR (2012). Cholesterol catabolism by *Mycobacterium tuberculosis* requires transcriptional and metabolic adaptations. Chem Biol.

[63] Voskuil MI (2013). *Mycobacterium tuberculosis* cholesterol catabolism requires a new class of acyl coenzyme a dehydrogenase. J Bacteriol.

[64] Kaplan G, Post FA, Moreira AL, Wainwright H, Kreiswirth BN, Tanverdi M (2003). *Mycobacterium tuberculosis* growth at the cavity surface: a microenvironment with failed immunity. Infect Immun.

[65] Lee W, VanderVen BC, Fahey RJ, Russell DG (2013). Intracellular *Mycobacterium tuberculosis* exploits host-derived fatty acids to limit metabolic stress. J Biol Chem.

[66] Savvi S, Warner DF, Kana BD, McKinney JD, Mizrahi V, Dawes SS (2008). Functional characterization of a vitamin B12-dependent methylmalonyl pathway in *Mycobacterium tuberculosis*: implications for propionate metabolism during growth on fatty acids. J Bacteriol.

[67] Gopinath K, Venclovas Č, Ioerger TR, Sacchettini JC, JD MK, Mizrahi V (2013). A vitamin B12 transporter in *Mycobacterium tuberculosis*. Open Biol.

[68] Gopinath K, Moosa A, Mizrahi V, Warner DF (2013). Vitamin B12 metabolism in *Mycobacterium tuberculosis*. Future Microbiol.

[69] Eoh H, Rhee KY (2014). Methylcitrate cycle defines the bactericidal essentiality of isocitrate lyase for survival of *Mycobacterium tuberculosis* on fatty acids. Proc Natl Acad Sci.

[70] Beste DJ, Nöh K, Niedenführ S, Mendum TA, Hawkins ND, Ward JL (2013). 13 C-flux spectral analysis of host-pathogen metabolism reveals a mixed diet for intracellular *Mycobacterium tuberculosis*. Chem Biol.

[71] Gouzy A, Larrouy-Maumus G, Bottai D, Levillain F, Dumas A, Wallach JB (2014). *Mycobacterium tuberculosis* exploits asparagine to assimilate nitrogen and resist acid stress during infection. PLoS Pathog.

[72] Gouzy A, Poquet Y, Neyrolles O (2014). Nitrogen metabolism in *Mycobacterium tuberculosis* physiology and virulence. Nat Rev Microbiol.

[73] Giffin MM, Modesti L, Raab RW, Wayne LG, Sohaskey CD (2012). Ald of *Mycobacterium tuberculosis* encodes both the alanine dehydrogenase and the putative glycine dehydrogenase. J Bacteriol.

[74] Rücker N, Billig S, Bücker R, Jahn D, Wittmann C, Bange F-C (2015). Acetate dissimilation and assimilation in *Mycobacterium tuberculosis* depend on carbon availability. J Bacteriol.

[75] Benning MM, Haller T, Gerlt JA, Holden HM. New reactions in the Crotonase superfamily: structure of Methylmalonyl CoA decarboxylase from *Escherichia coli*†. Biochemistry (Mosc). 2000;39:4630–9. 10.1021/bi9928896 10769118

[76] de Carvalho LPS, Fischer SM, Marrero J, Nathan C, Ehrt S, Rhee KY (2010). Metabolomics of *Mycobacterium tuberculosis* reveals compartmentalized co-catabolism of carbon substrates. Chem Biol.

[77] Ehrt S, Rhee K, Schnappinger D (2015). Mycobacterial genes essential for the pathogen’s survival in the host. Immunol Rev.

[78] Nandakumar M, Nathan C, Rhee K (2013). Isocitrate lyase mediates broad antibiotic tolerance in *Mycobacterium tuberculosis*. Nat Commun.

