# Supplementary figures and images for: Metabolic adaptation of two in silico mutants of Mycobacterium tuberculosis during infection
Source: BMC Syst Biol. 2017 Nov 21;11:107. doi: 10.1186/s12918-017-0496-z (PMC5697012; doi:10.1186/s12918-017-0496-z)

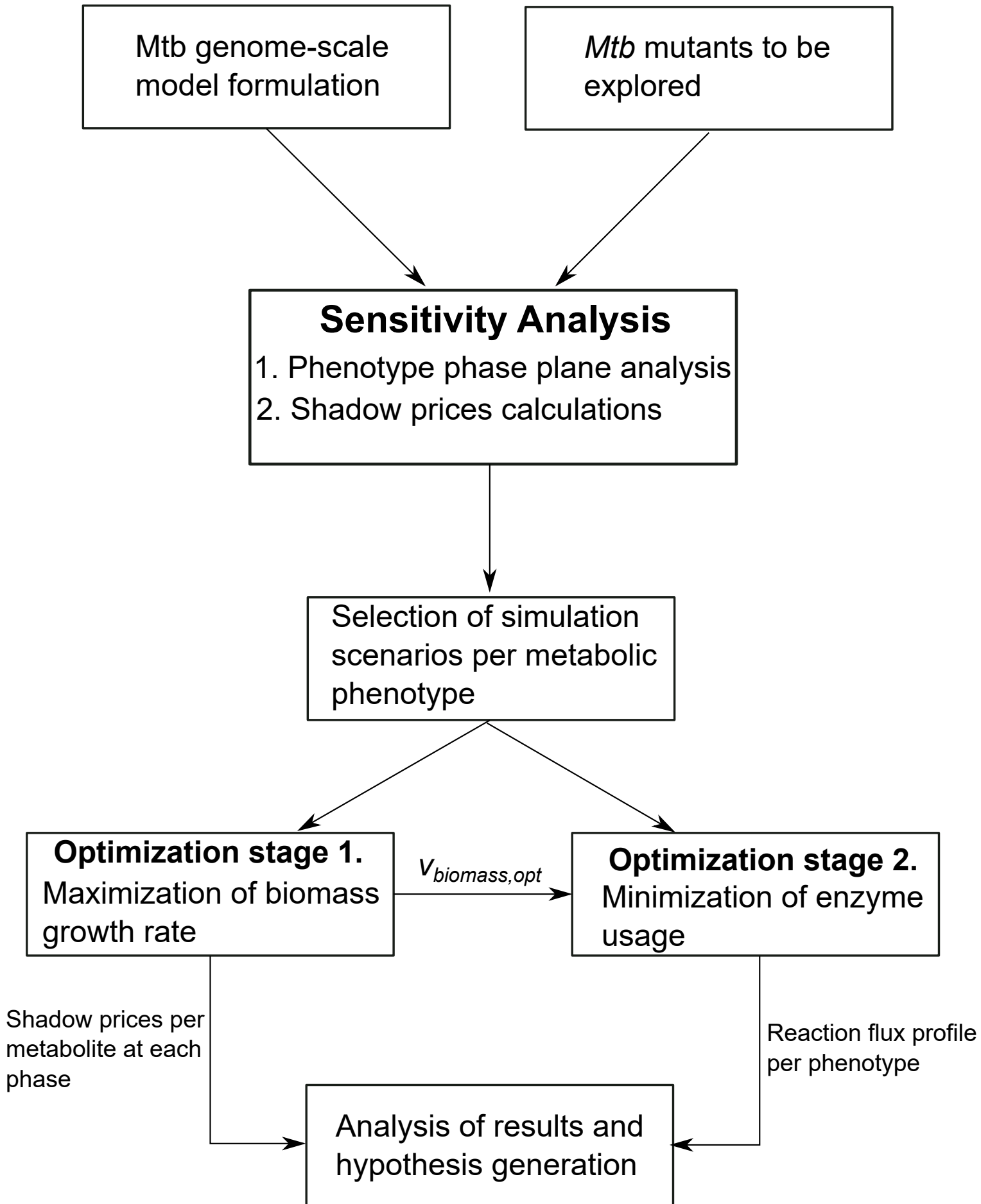

Supplement: Supplementary file 1 — Flowchart of the methodology adopted in this in silico study. (PDF 41 kb) [file 12918_2017_496_MOESM1_ESM.pdf]

a

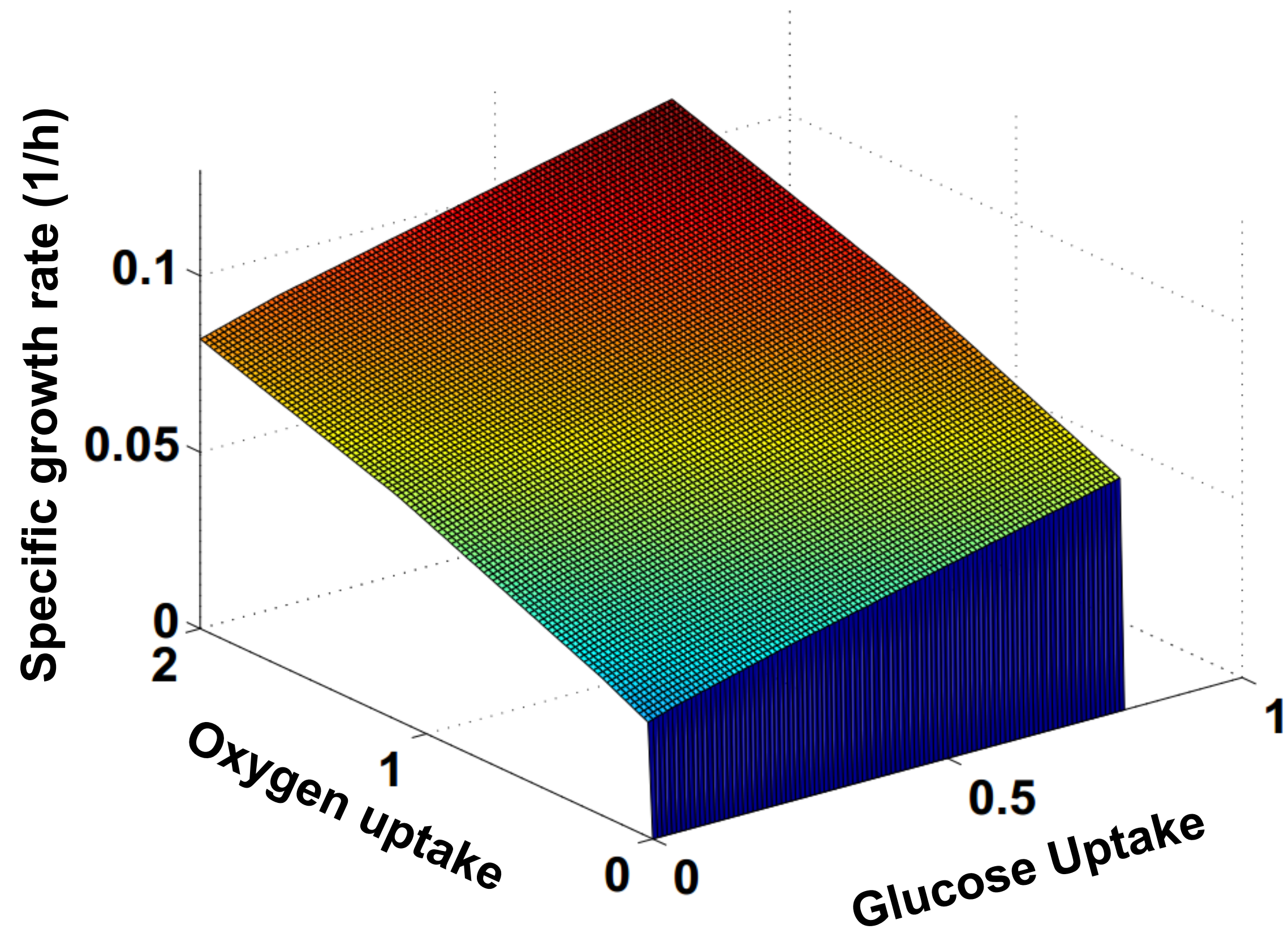

b

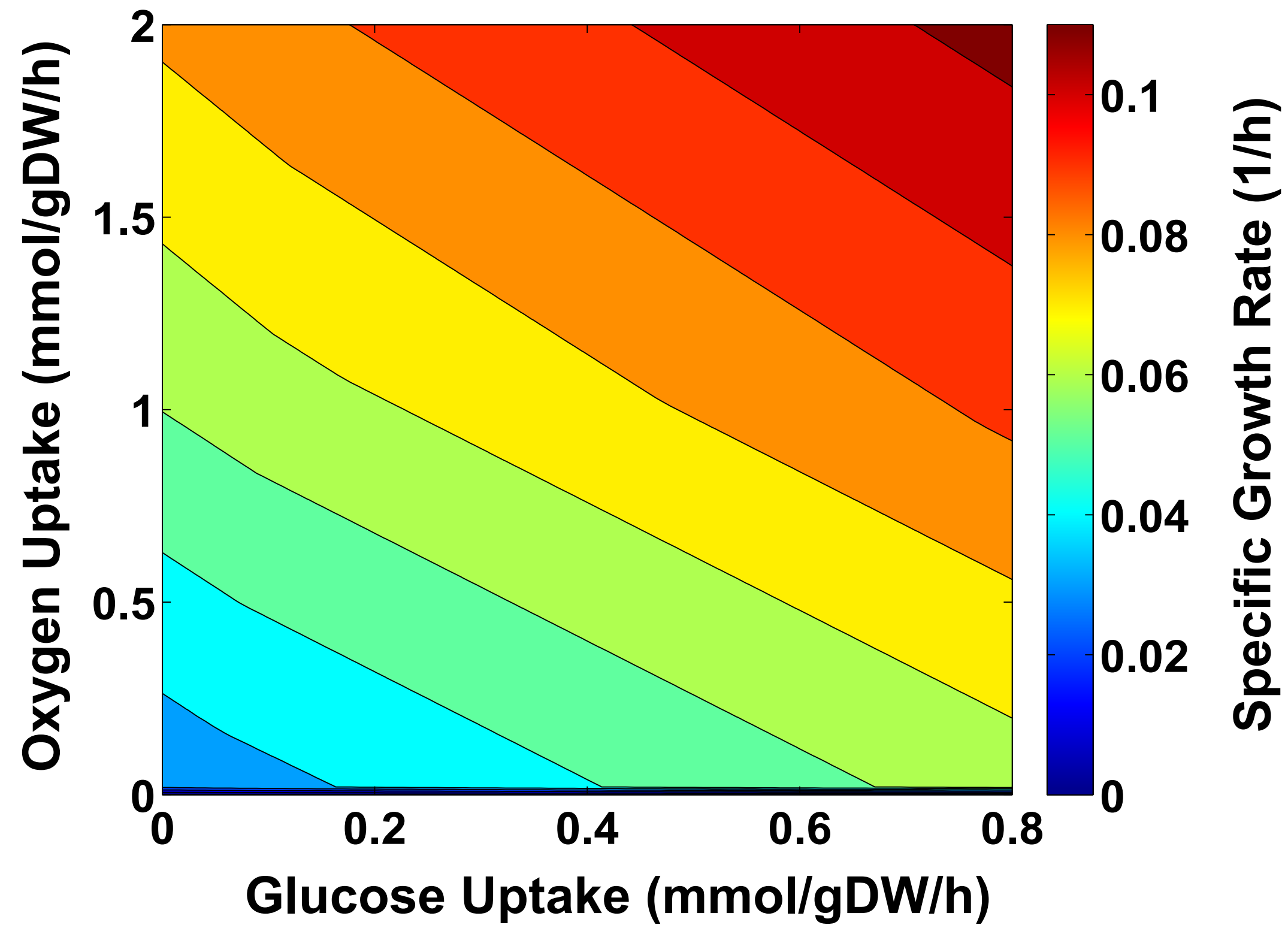

Supplement: Supplementary file 4 — Phenotypic phase planes of the Wild-Type Mtb during shifts of glucose and oxygen. (a) 3D top view of the PhPP. (b) 2D top view of the PhPP highlighting isoclines with negative slopes. (PDF 457 kb) [file 12918_2017_496_MOESM4_ESM.pdf]

Metabolic Flux (mmol/gDW/h)

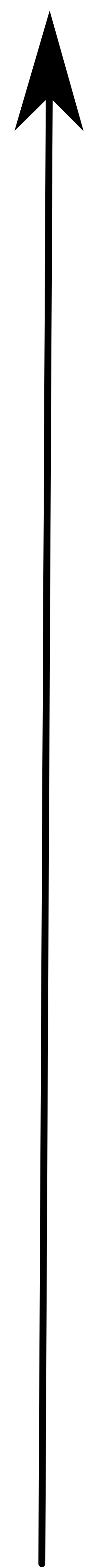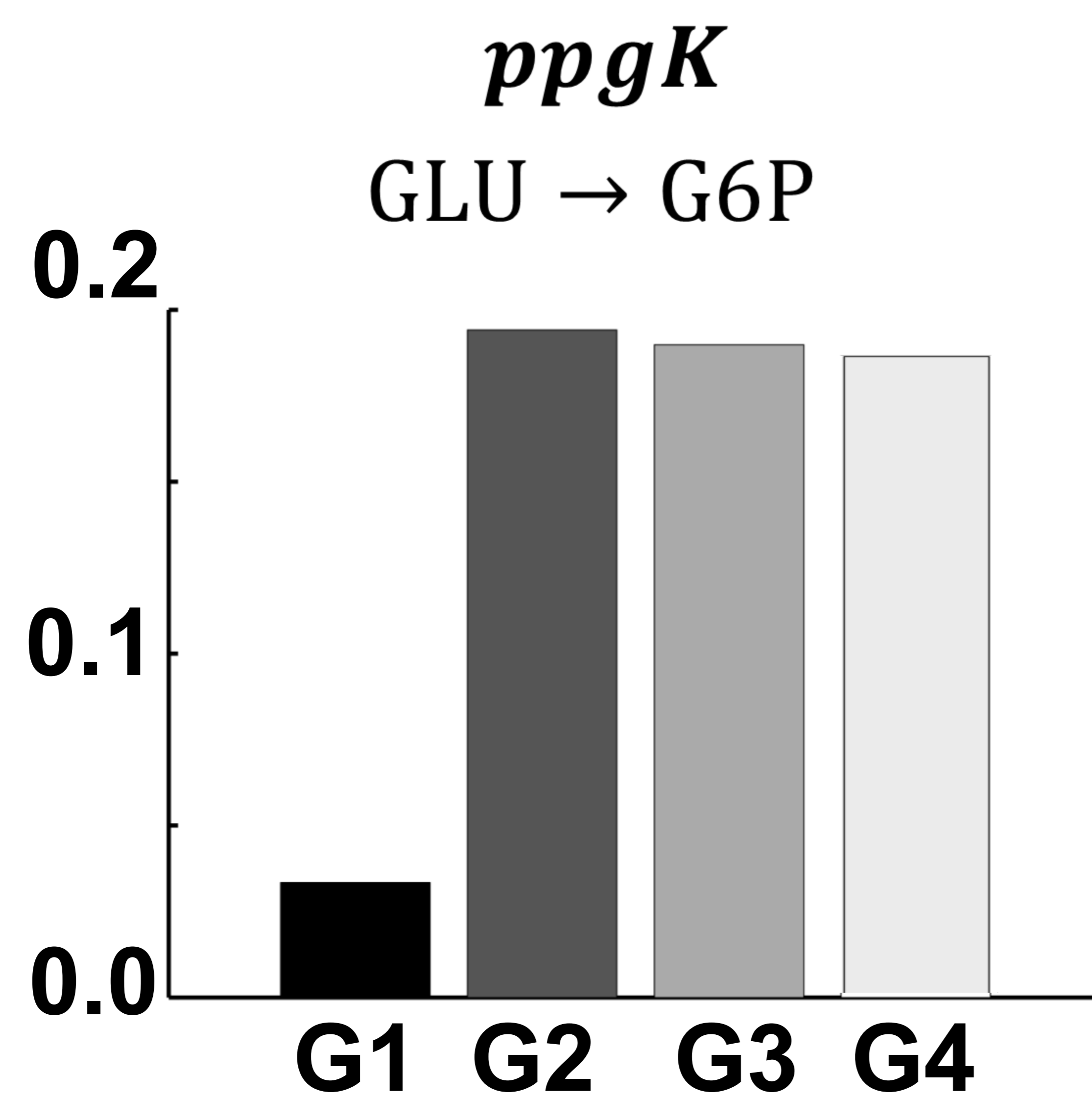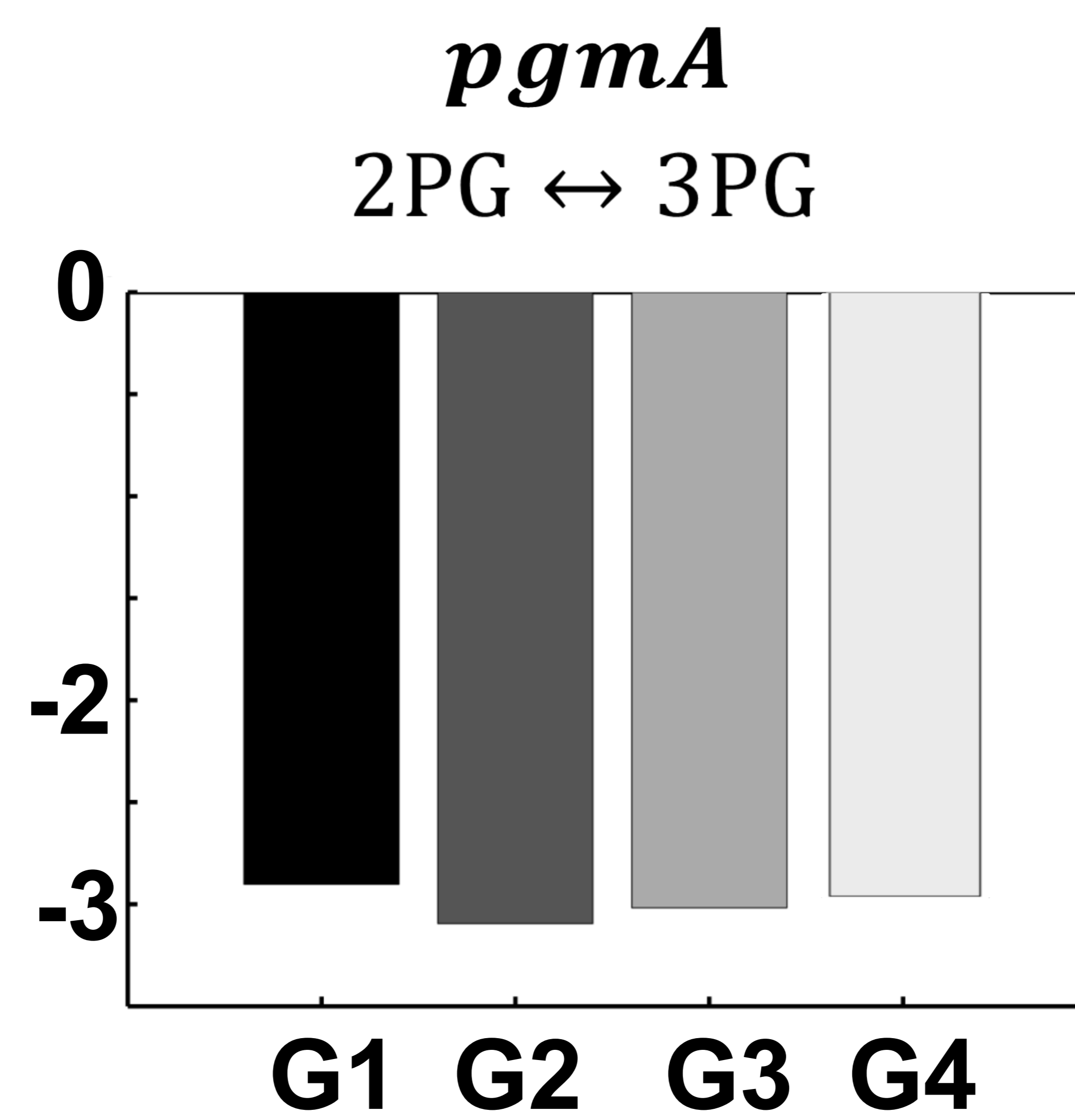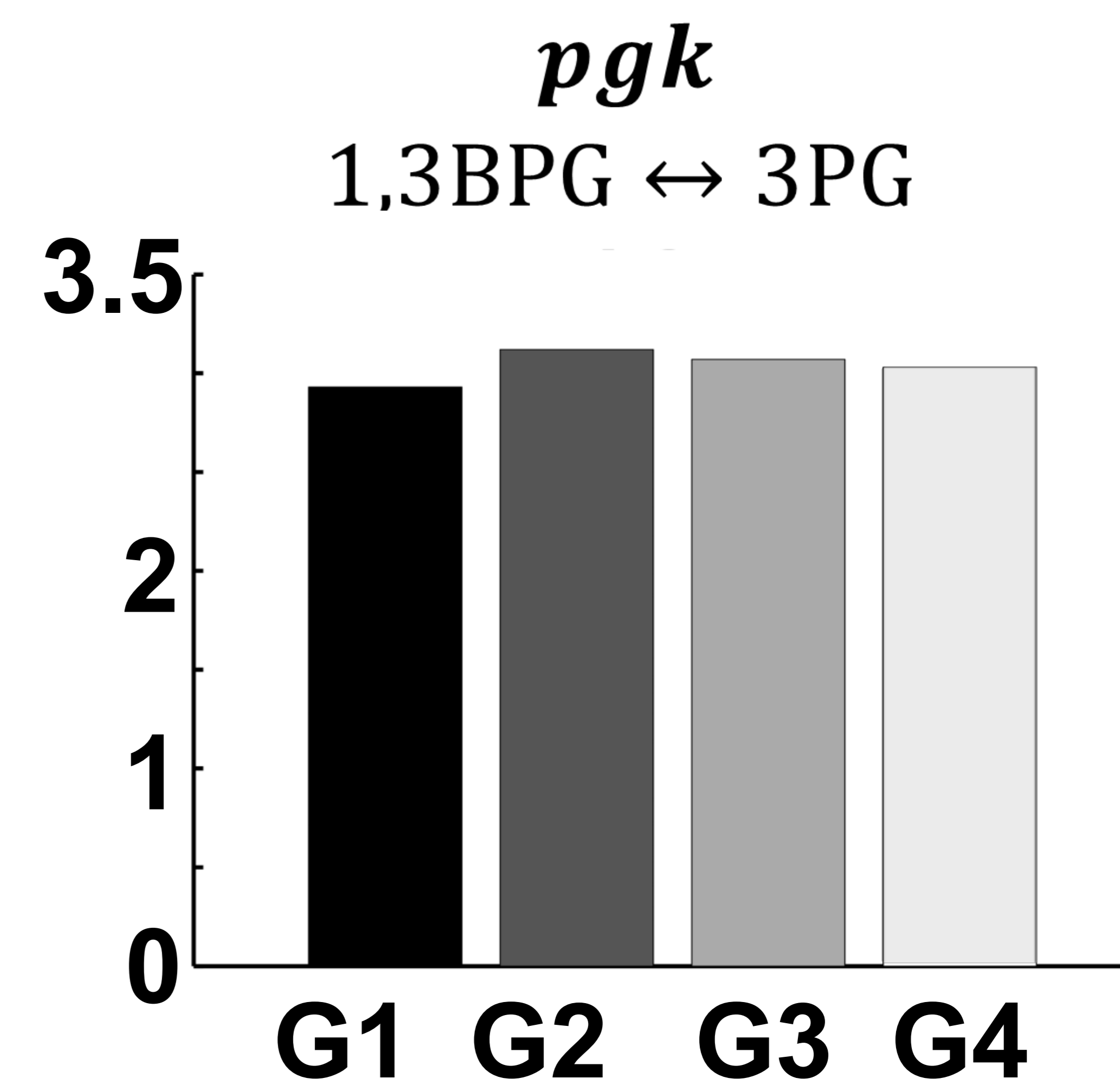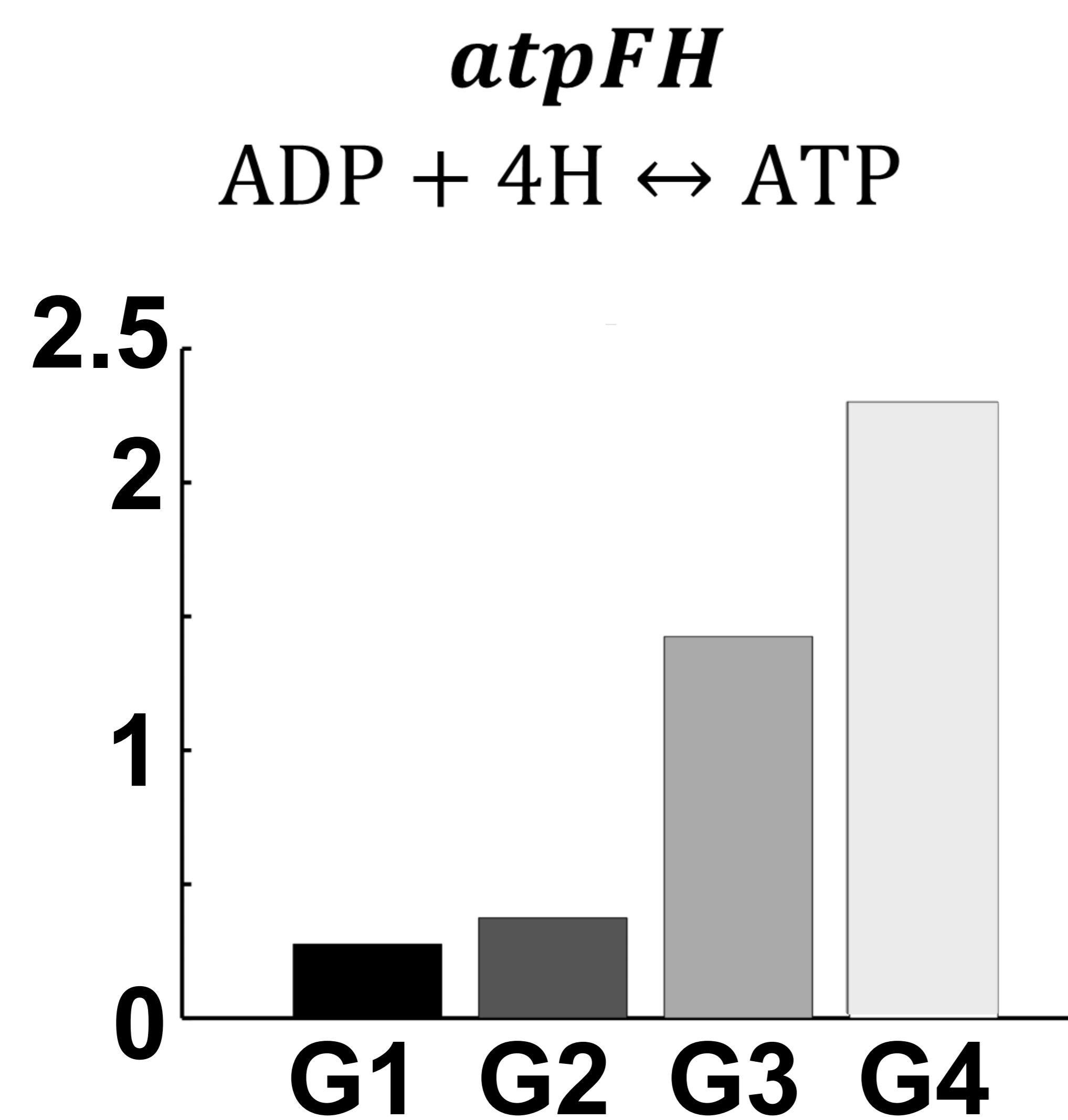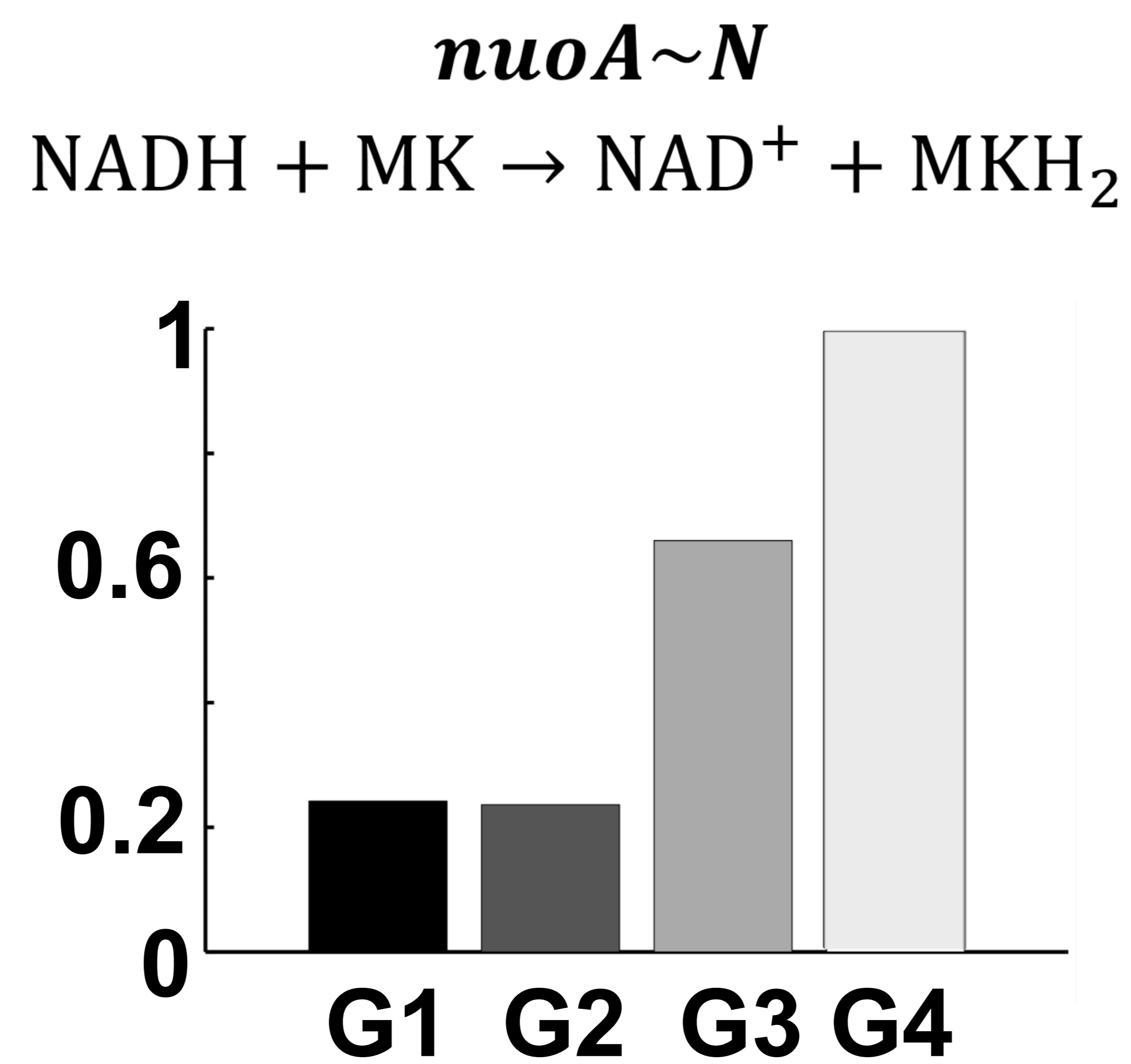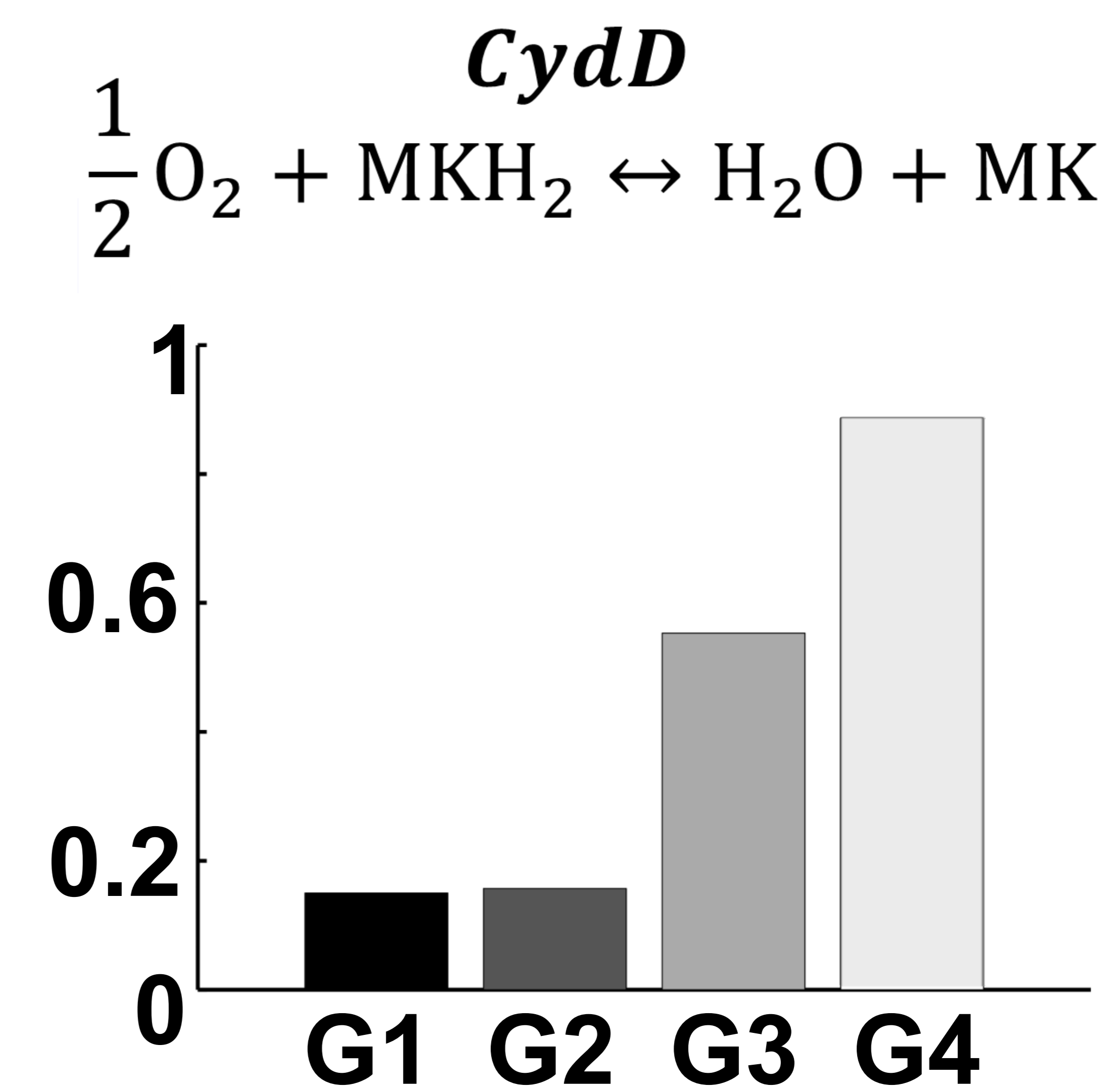

Supplement: Supplementary file 6 — Carbon flux distributions depicting other enzymes for the pfkA-mutant in phases G1, G2, G3, and G4. ppgK: polyphosphate glucokinase, pgmA: phosphoglucomutase, pgk: phosphoglycerate kinase, atpFH: ATP synthase, nuoA∼N: NADH dehydrogenase, cydD: cytochrome c oxido-reductase. (PDF 1119 kb) [file 12918_2017_496_MOESM6_ESM.pdf]

Metabolic Flux (mmol/gDW/h)

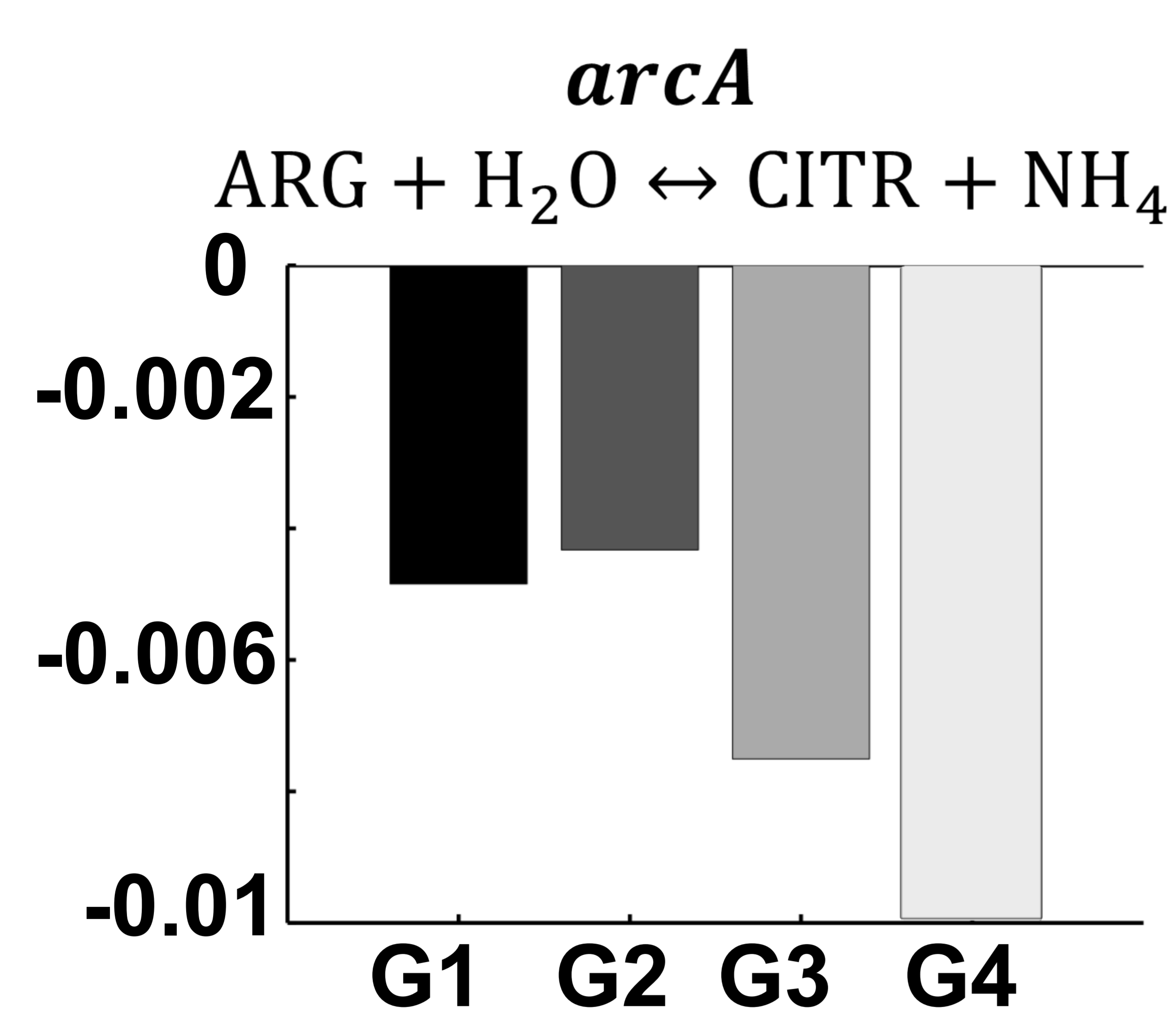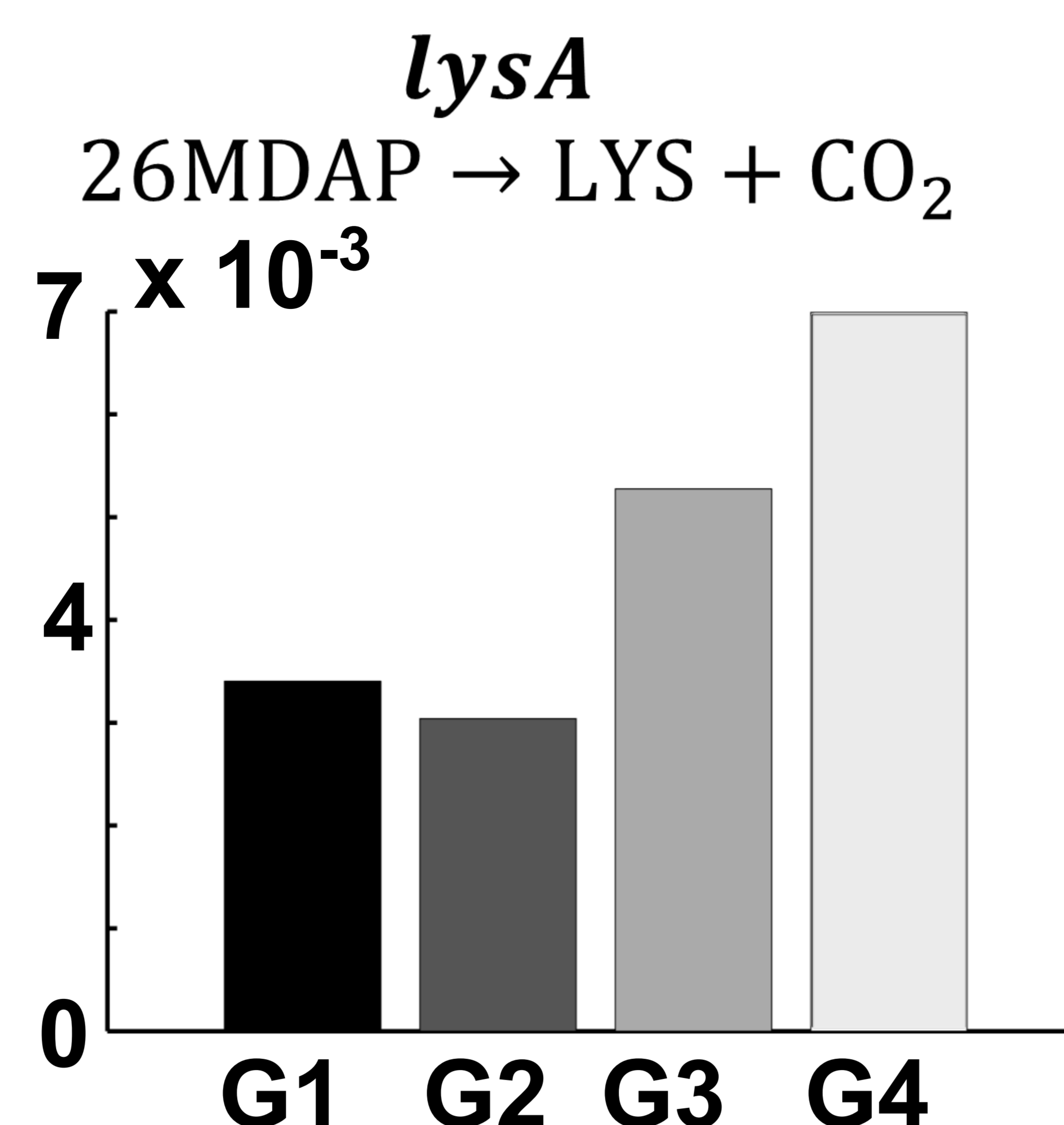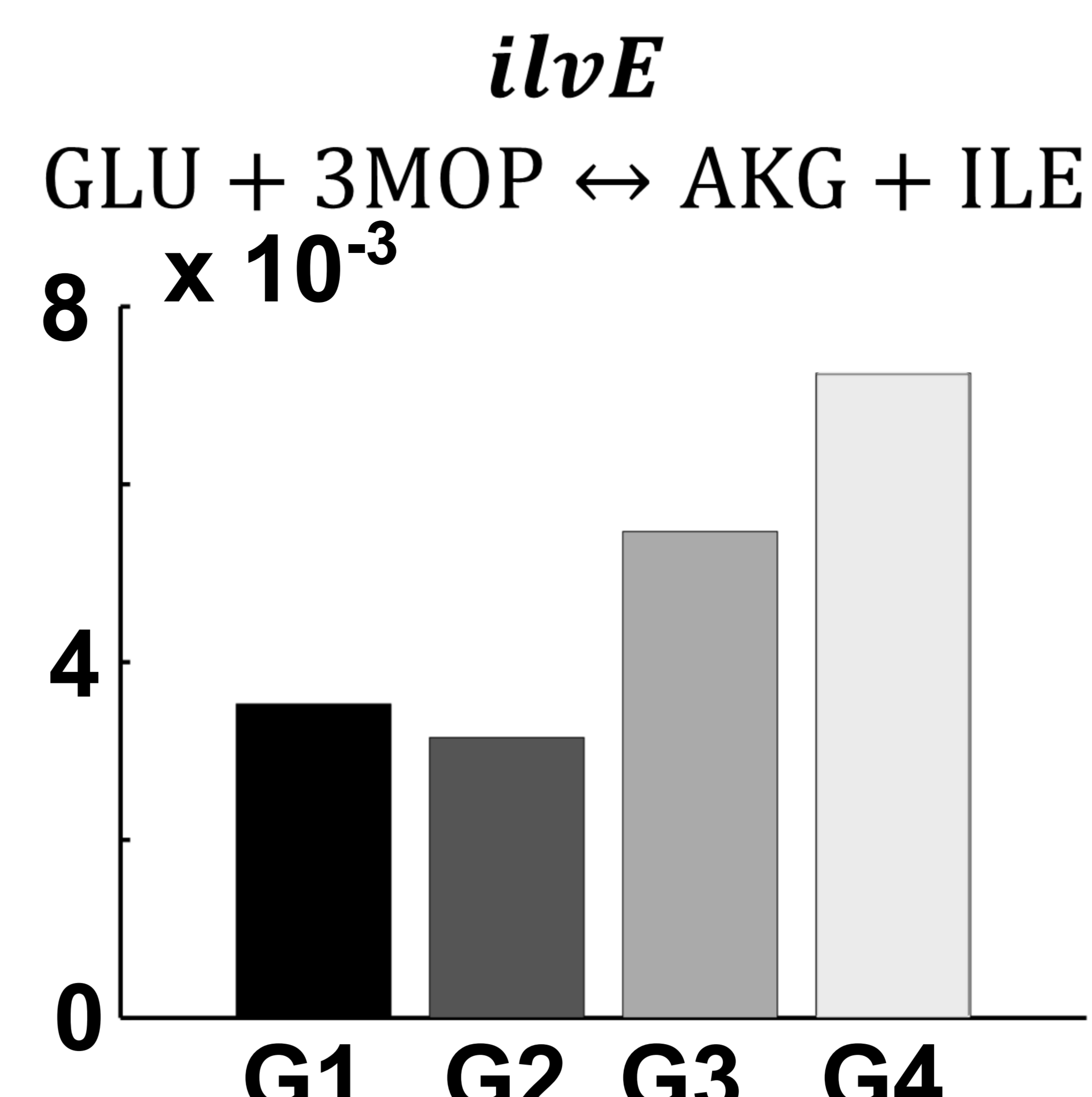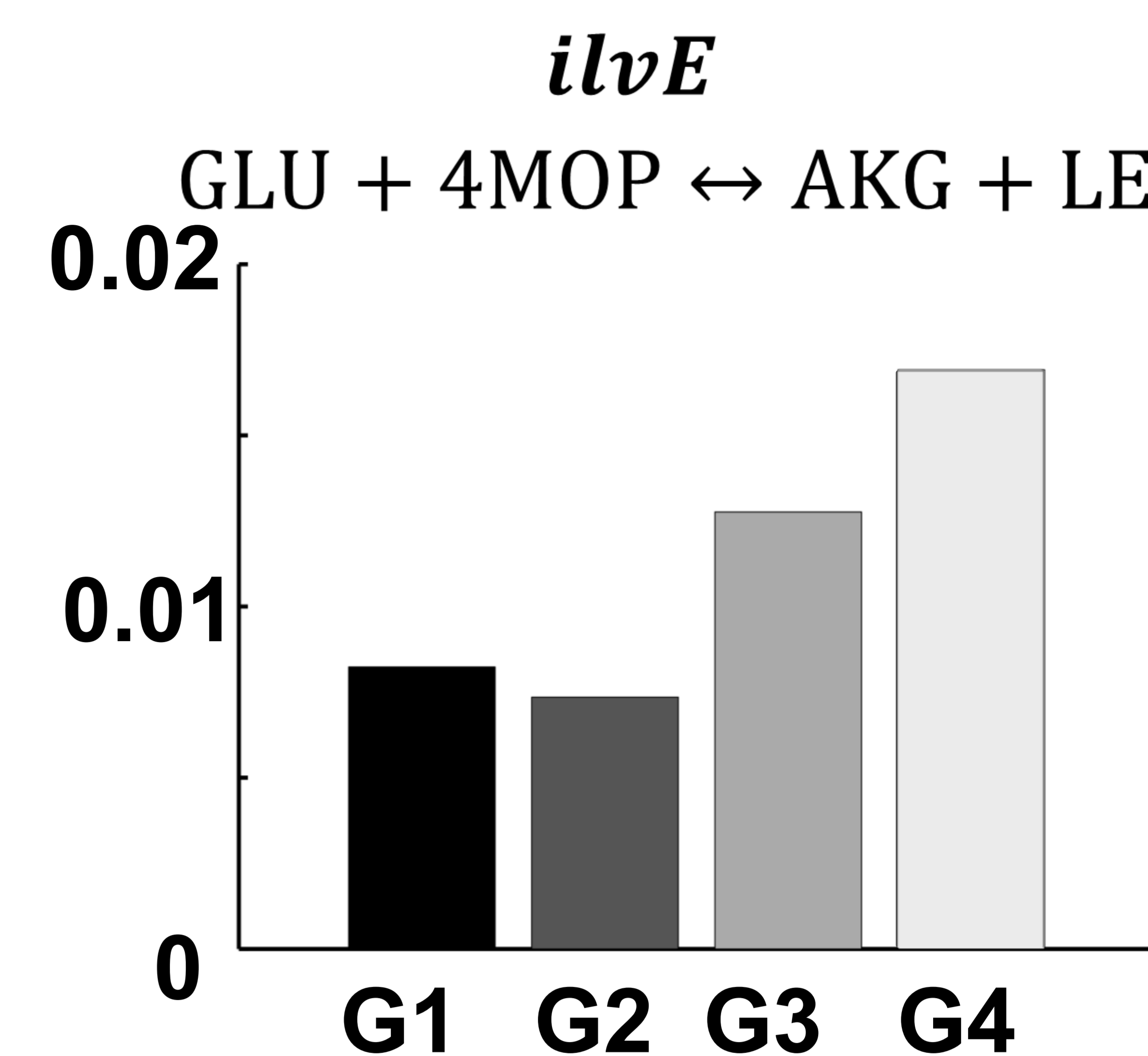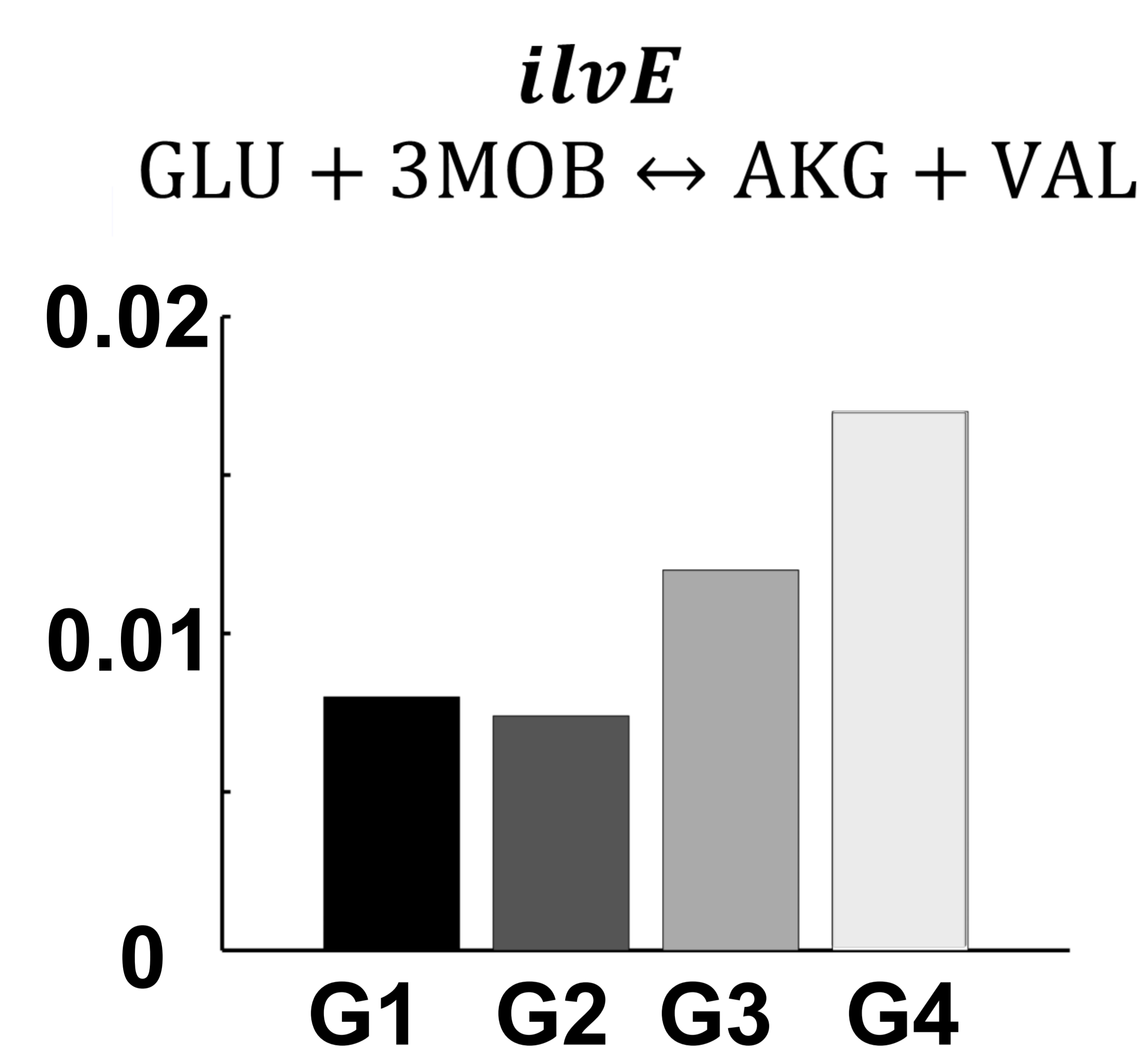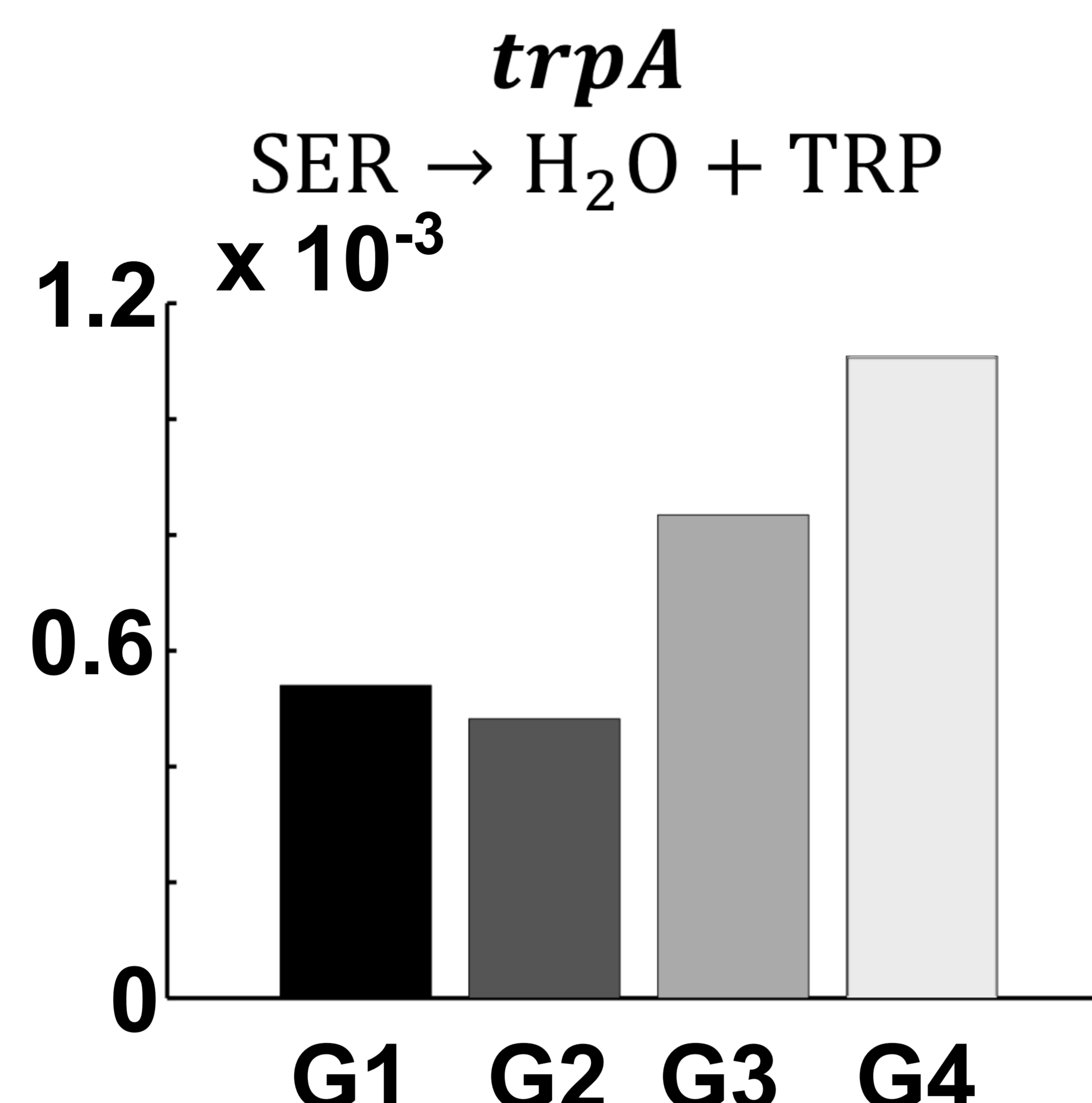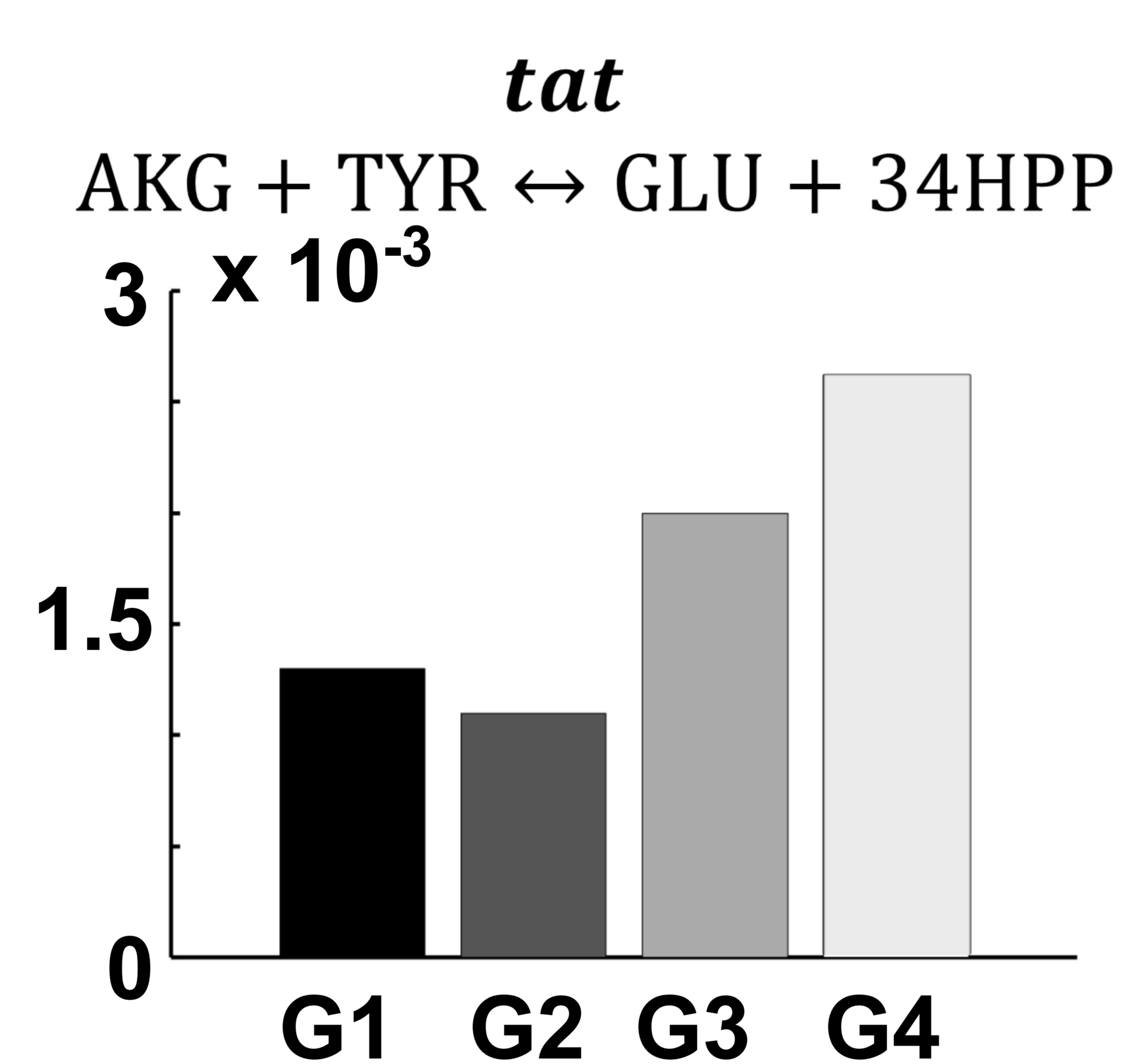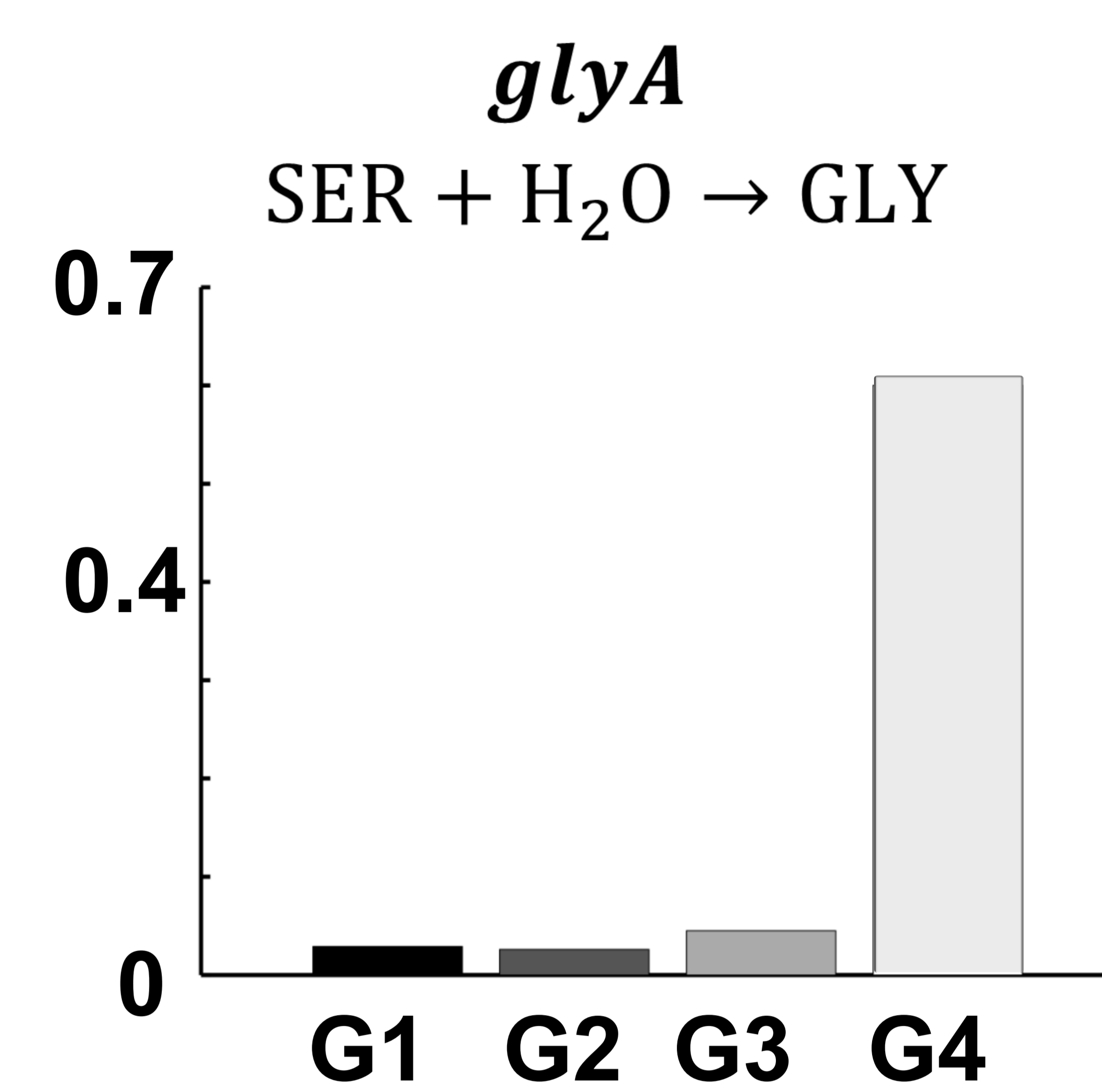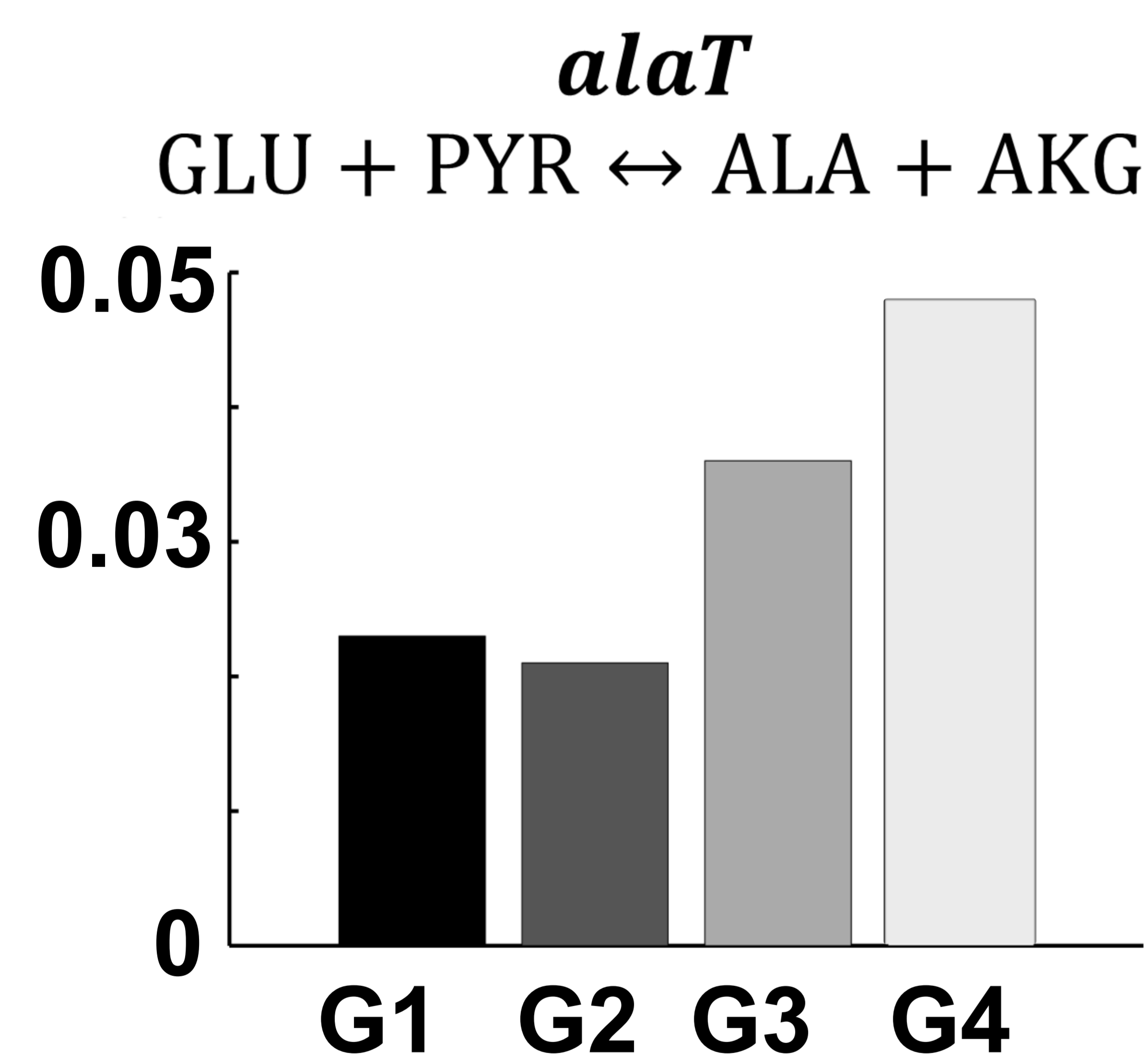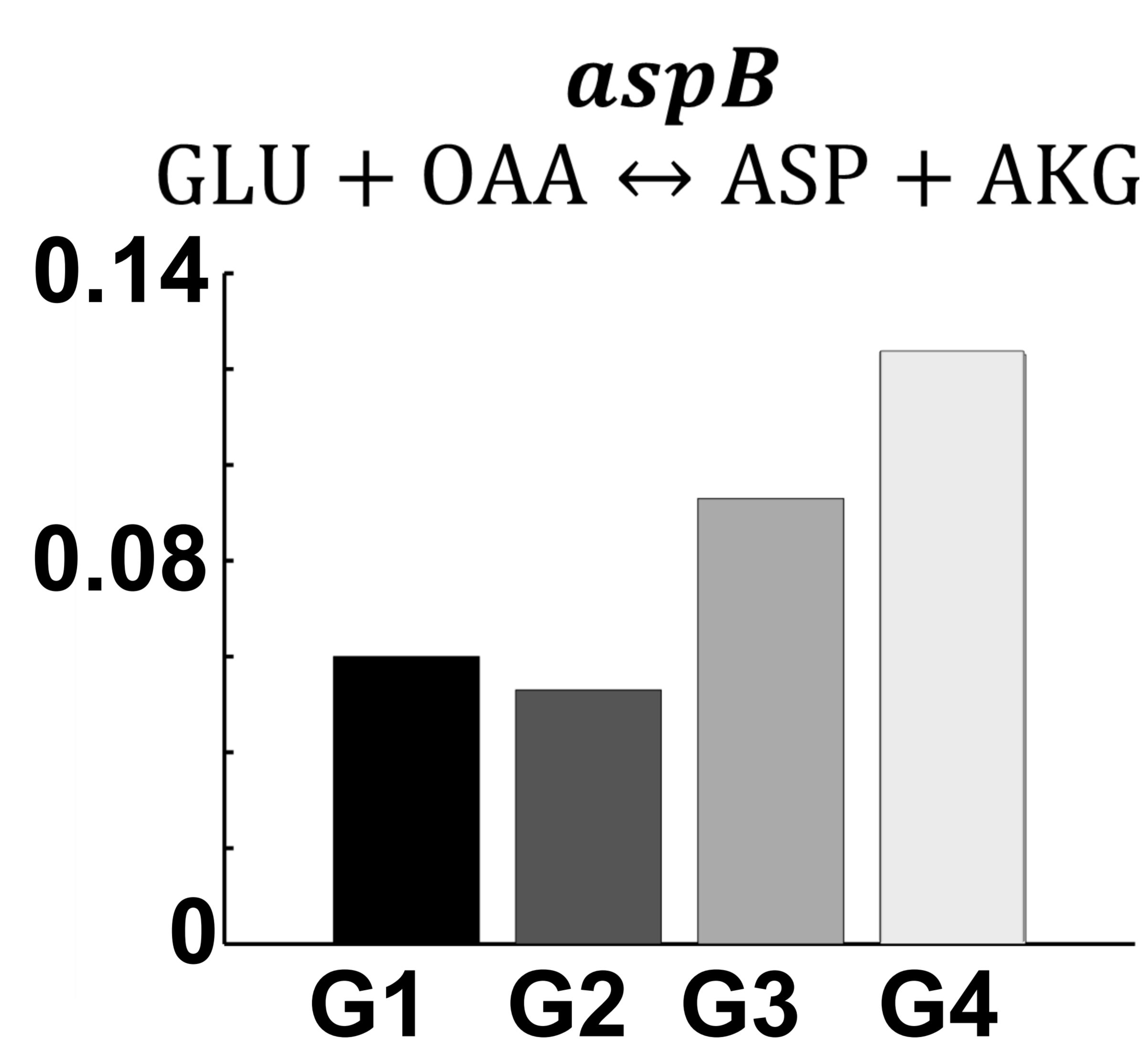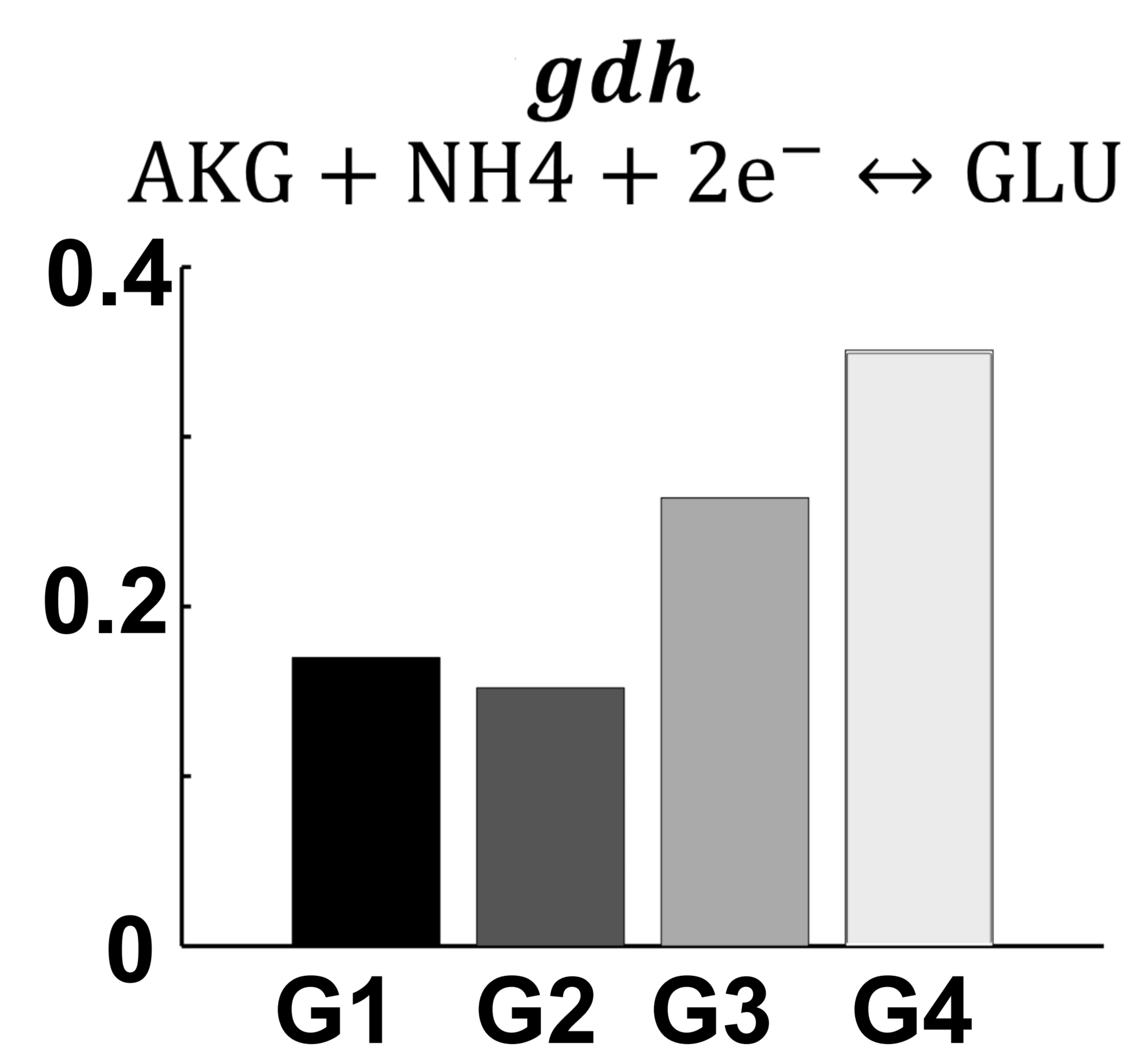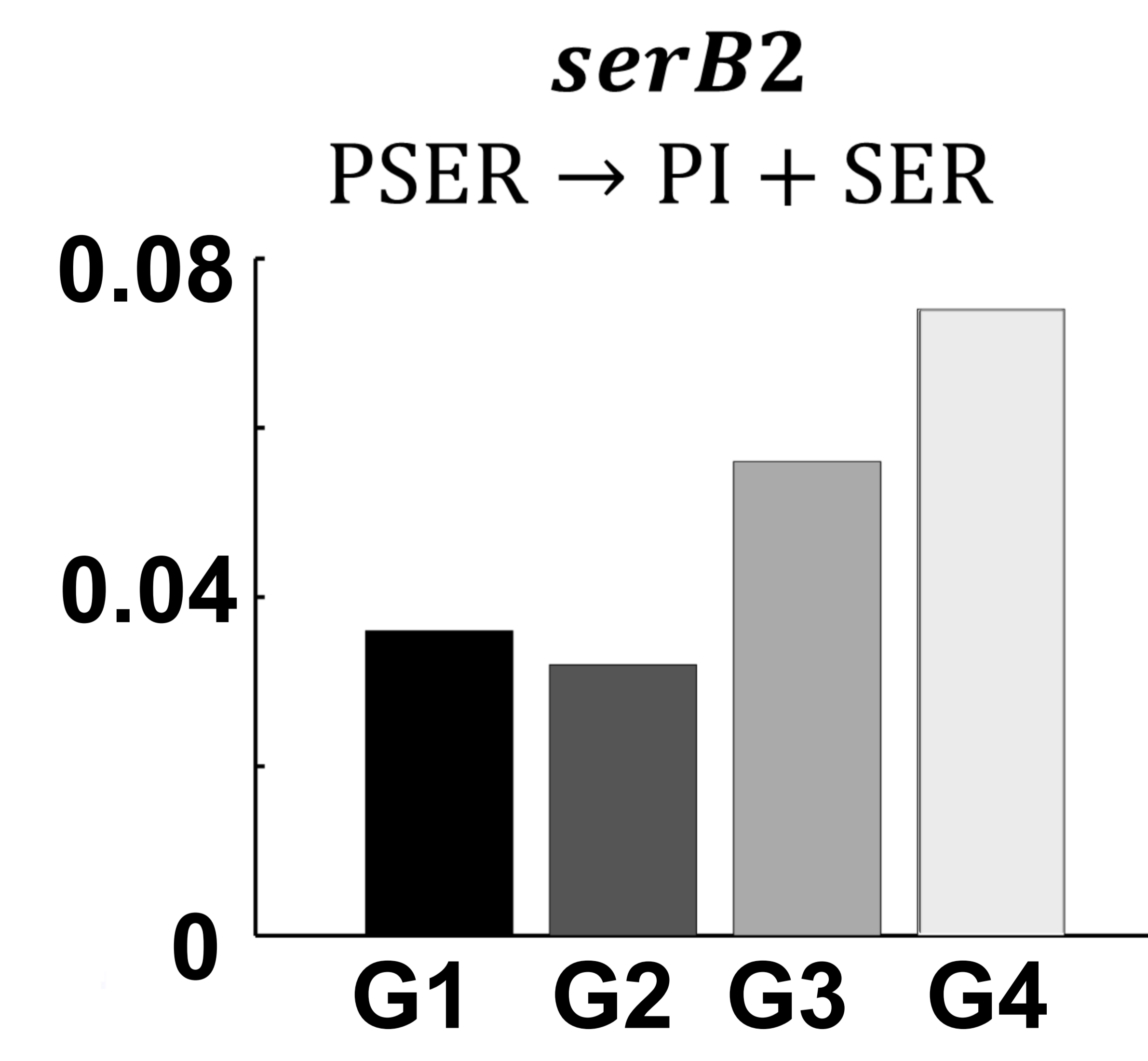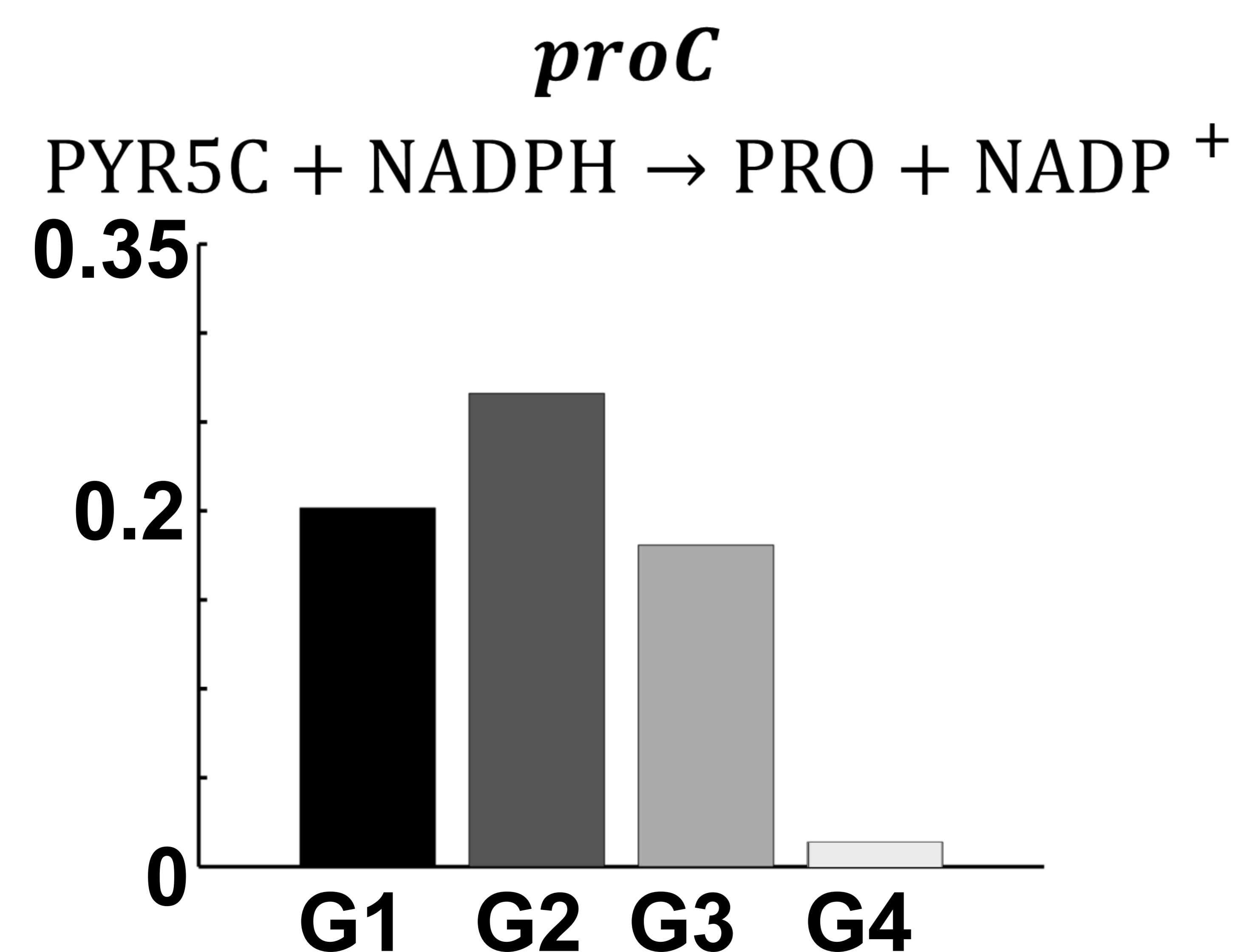

Supplement: Supplementary file 7 — Flux towards amino acid biosynthesis in phases G1, G2, G3 and G4 of the pfkA-mutant. arcA: arginine deiminase, lysA: diaminopimelate decarboxylase, ilvE: branched-chain-amino-acid aminotransferase, trpA: tryptophan synthase, tat: tyrosine aminotransferase, glyA: serine hydroxymethyltransferase, alaT: alanine aminotransferase, aspB: aspartate aminotransferase, gdh: glutamate dehydrogenase, serB2: phosphoserine phosphatase, proC: pyrroline-5-carboxylate reductase. (PDF 384 kb) [file 12918_2017_496_MOESM7_ESM.pdf]

**a**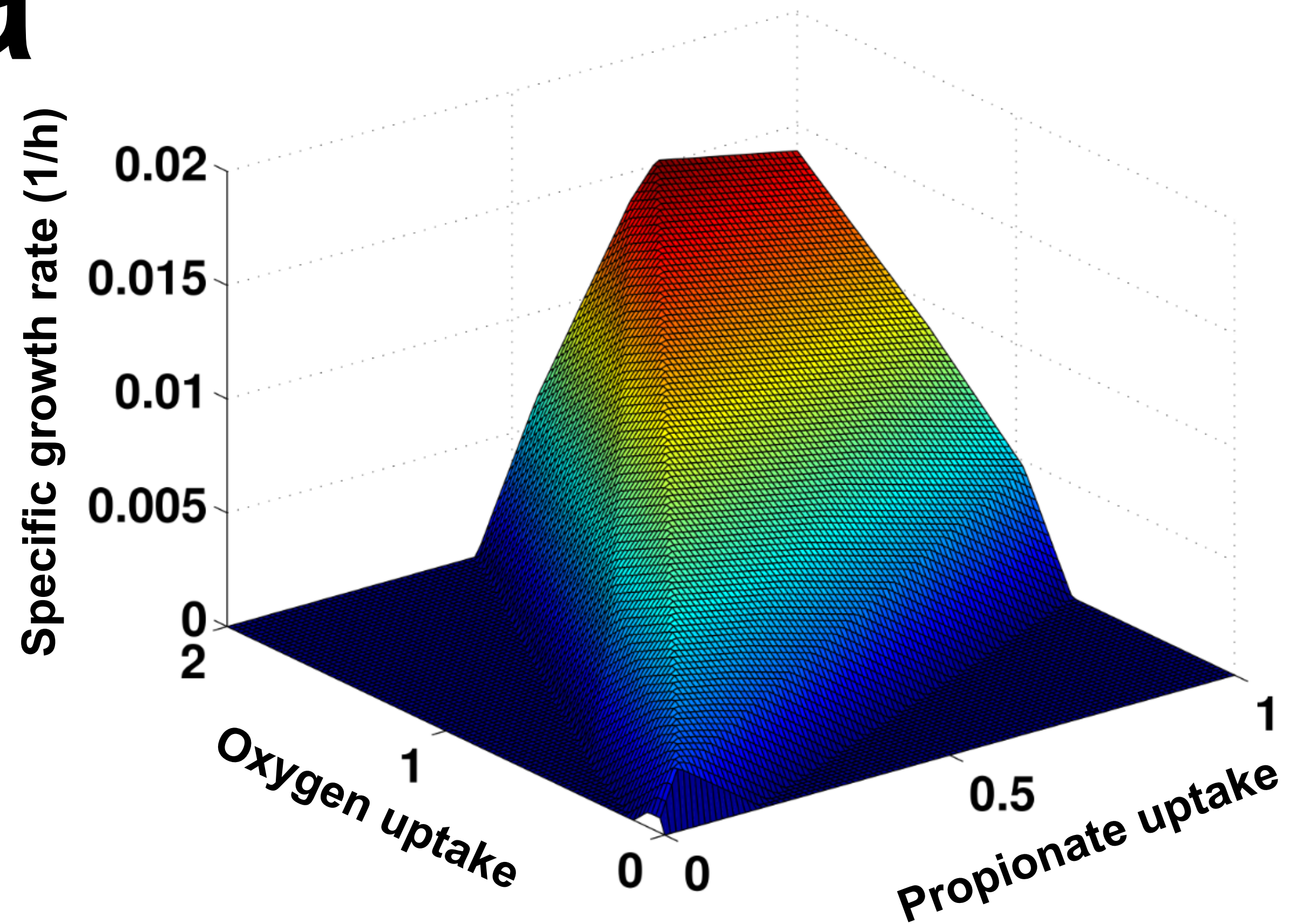**b**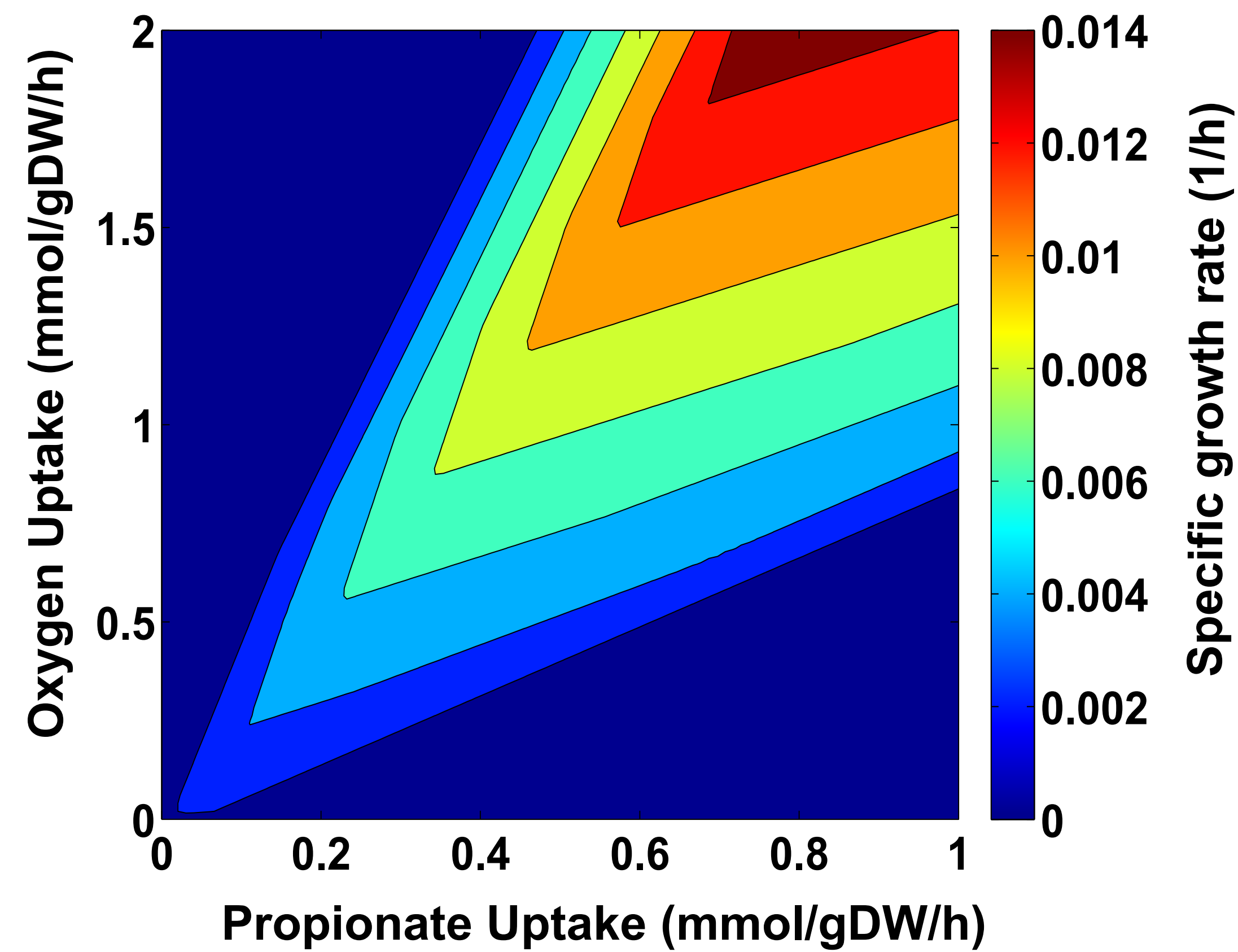

Supplement: Supplementary file 8 — Phenotypic phase planes of the Wild-Type Mtb during shifts of propionate and oxygen. (a) 3D top view of the PhPP. (b) 2D top view of the PhPP highlighting isoclines. (PDF 623 kb) [file 12918_2017_496_MOESM8_ESM.pdf]

Metabolic Flux (mmol/gDW/h)

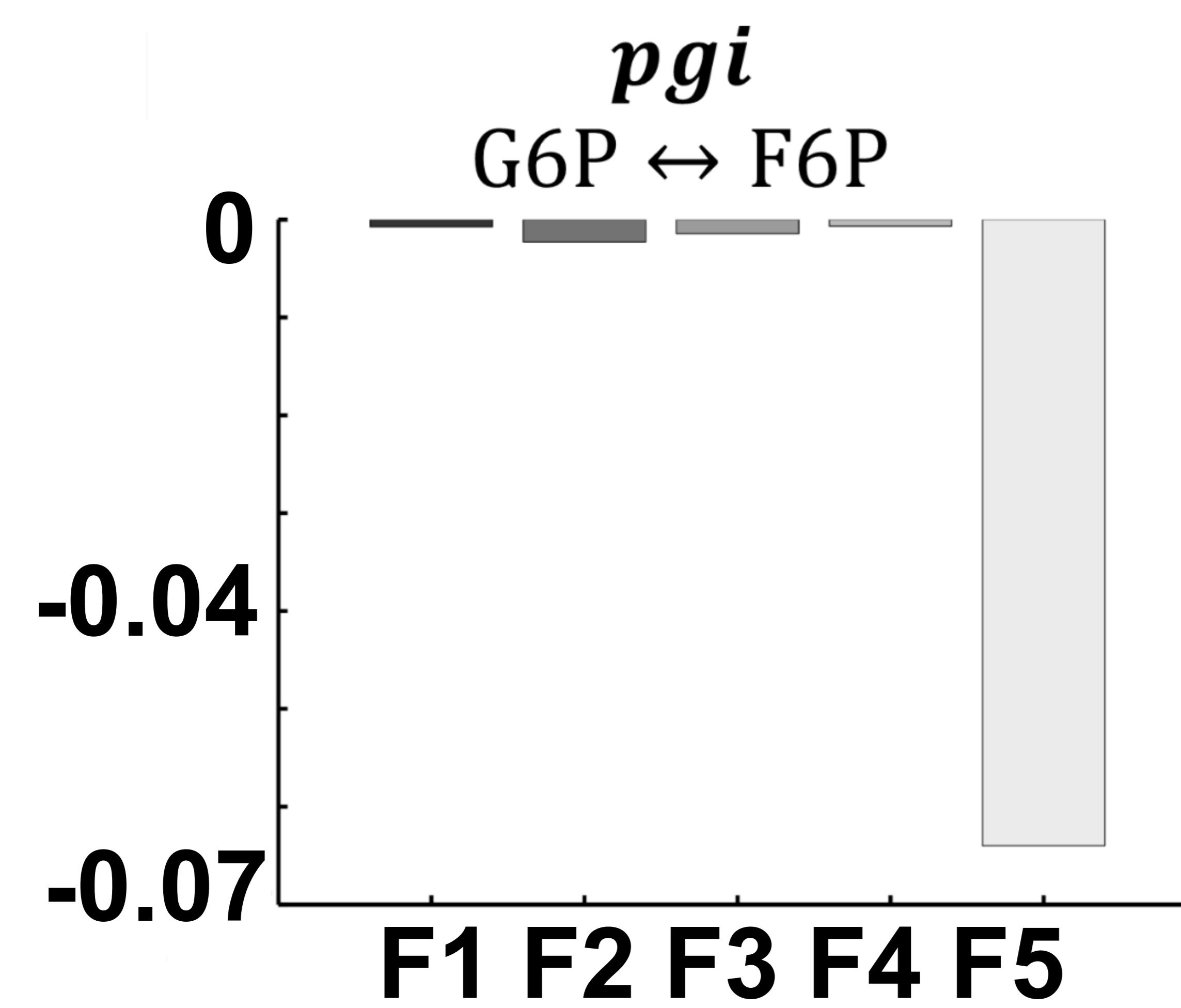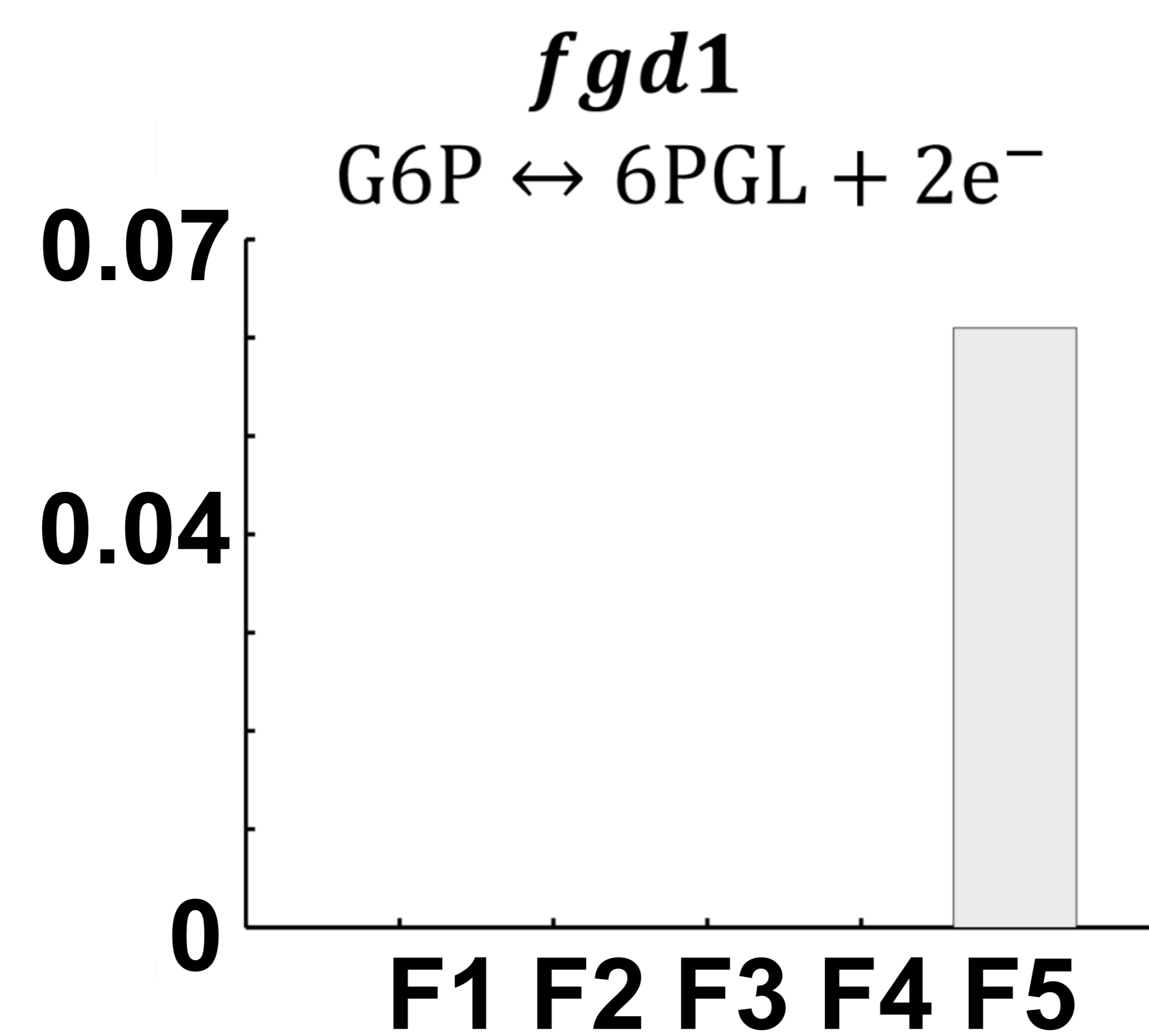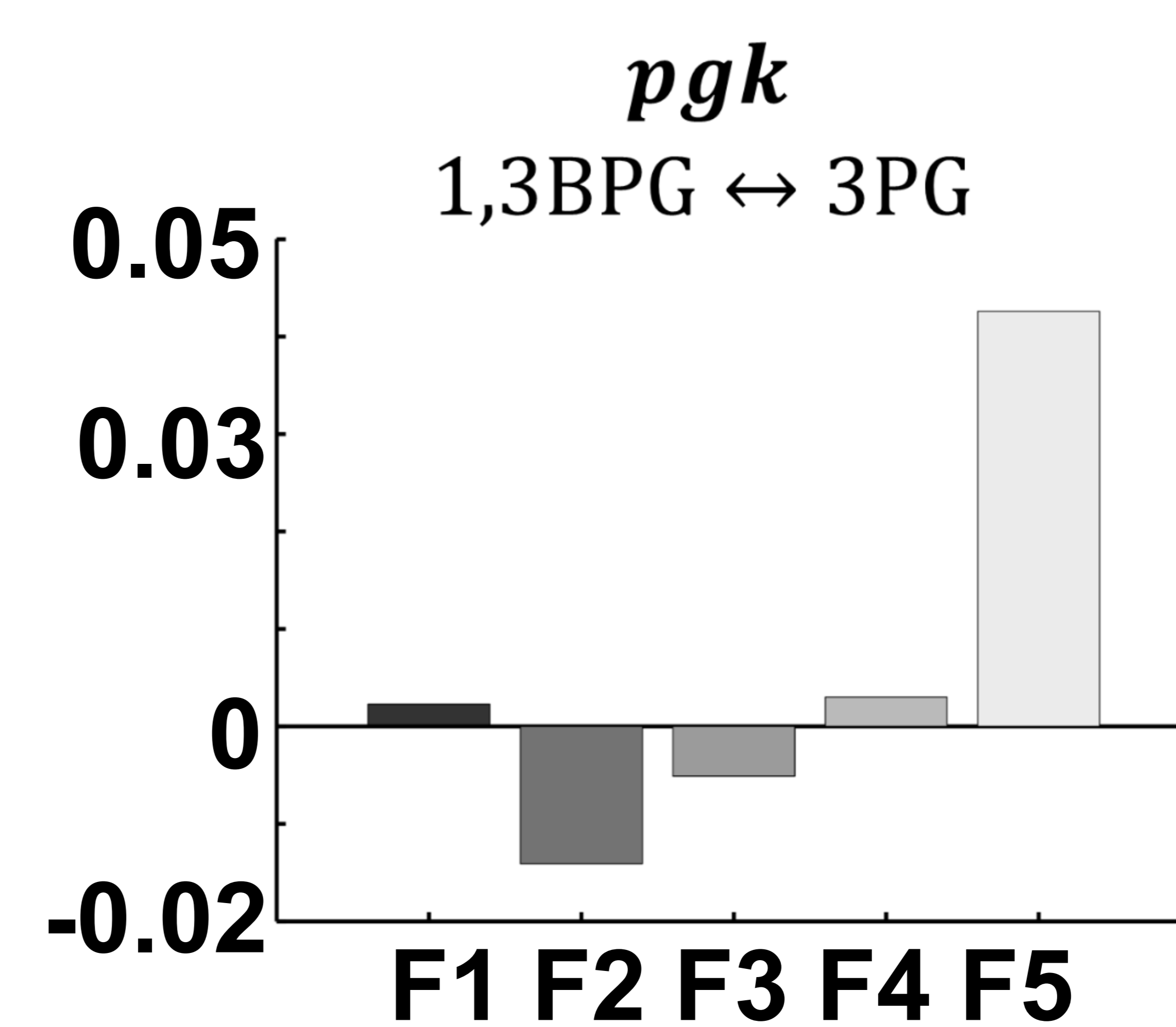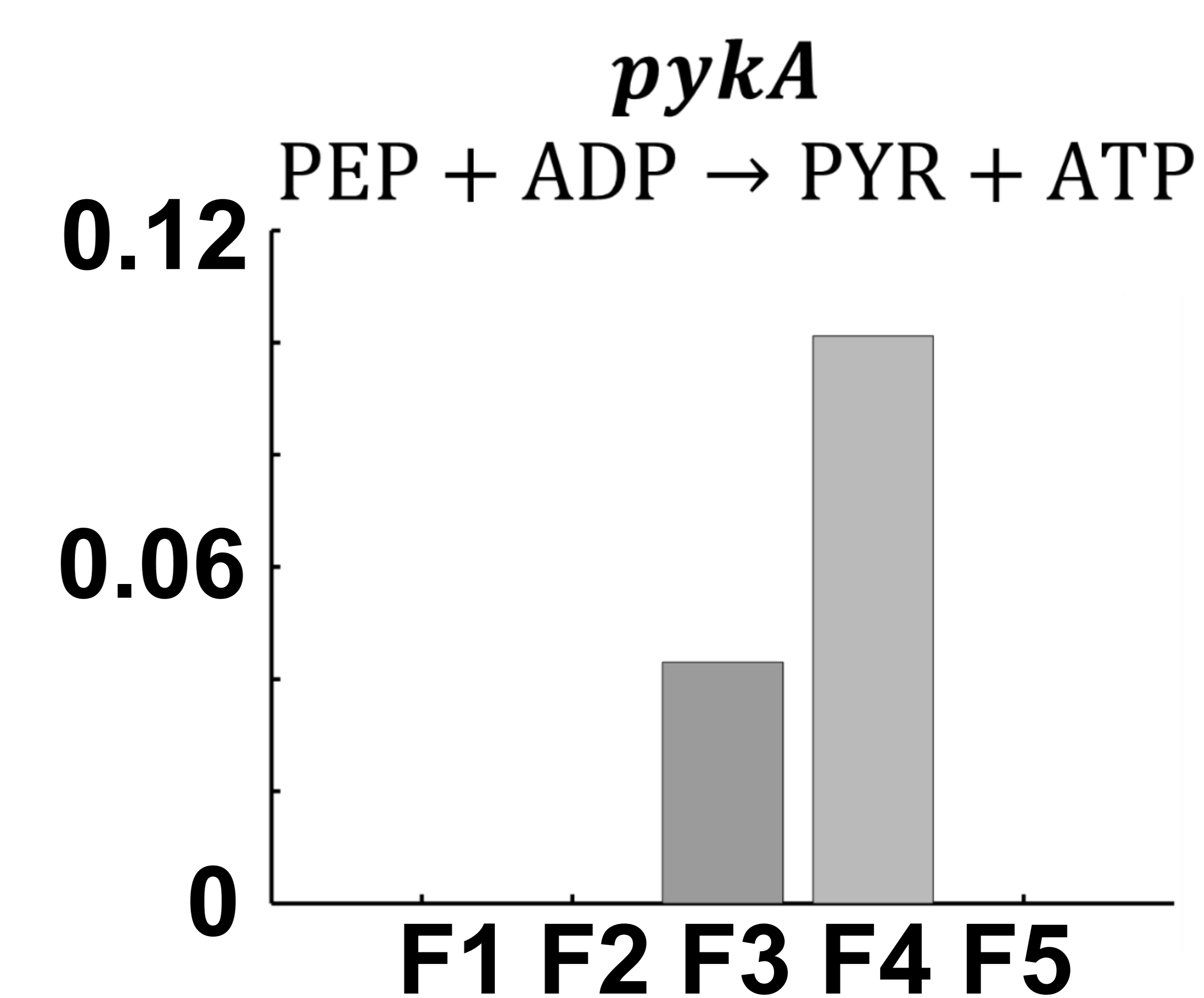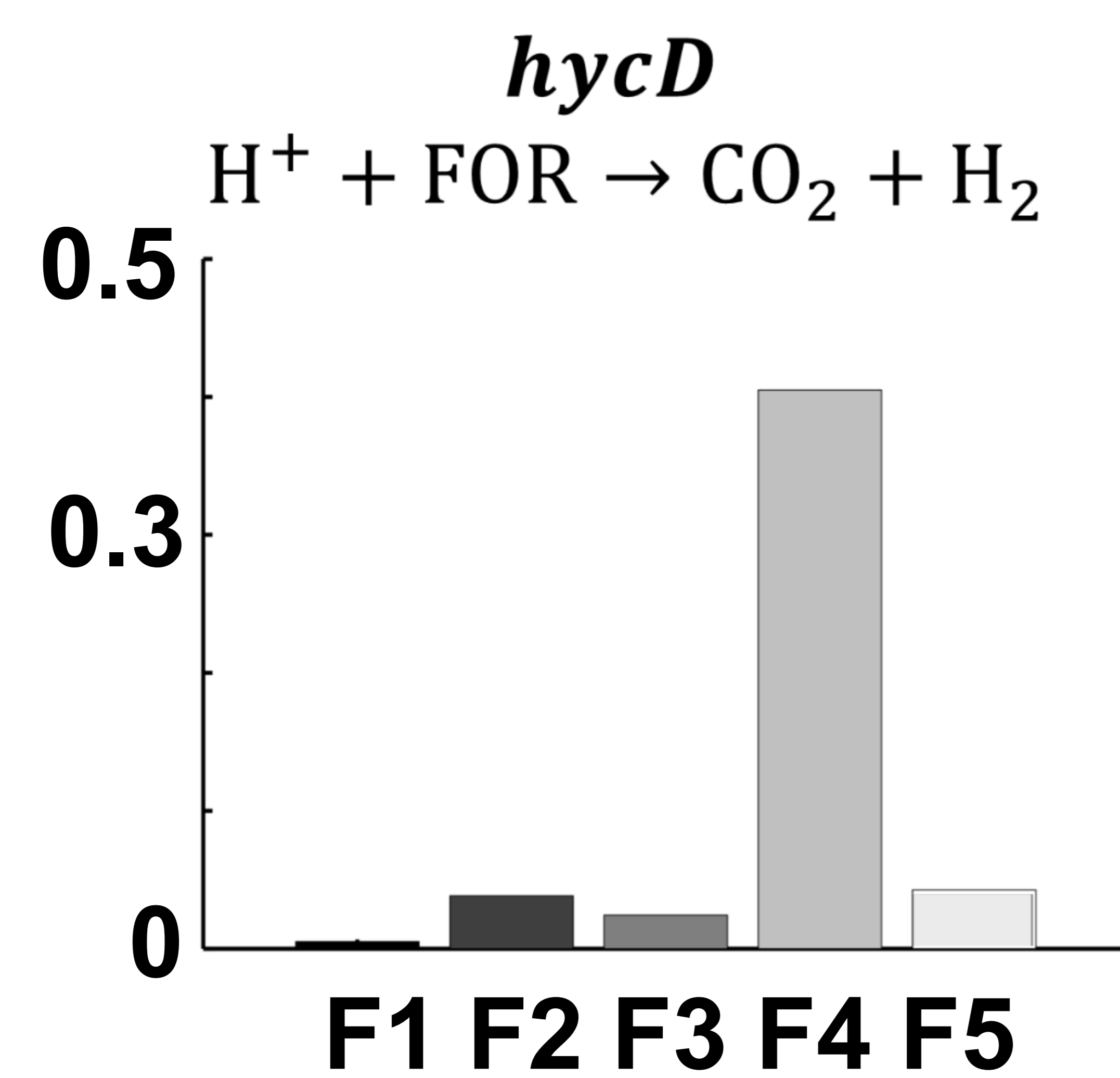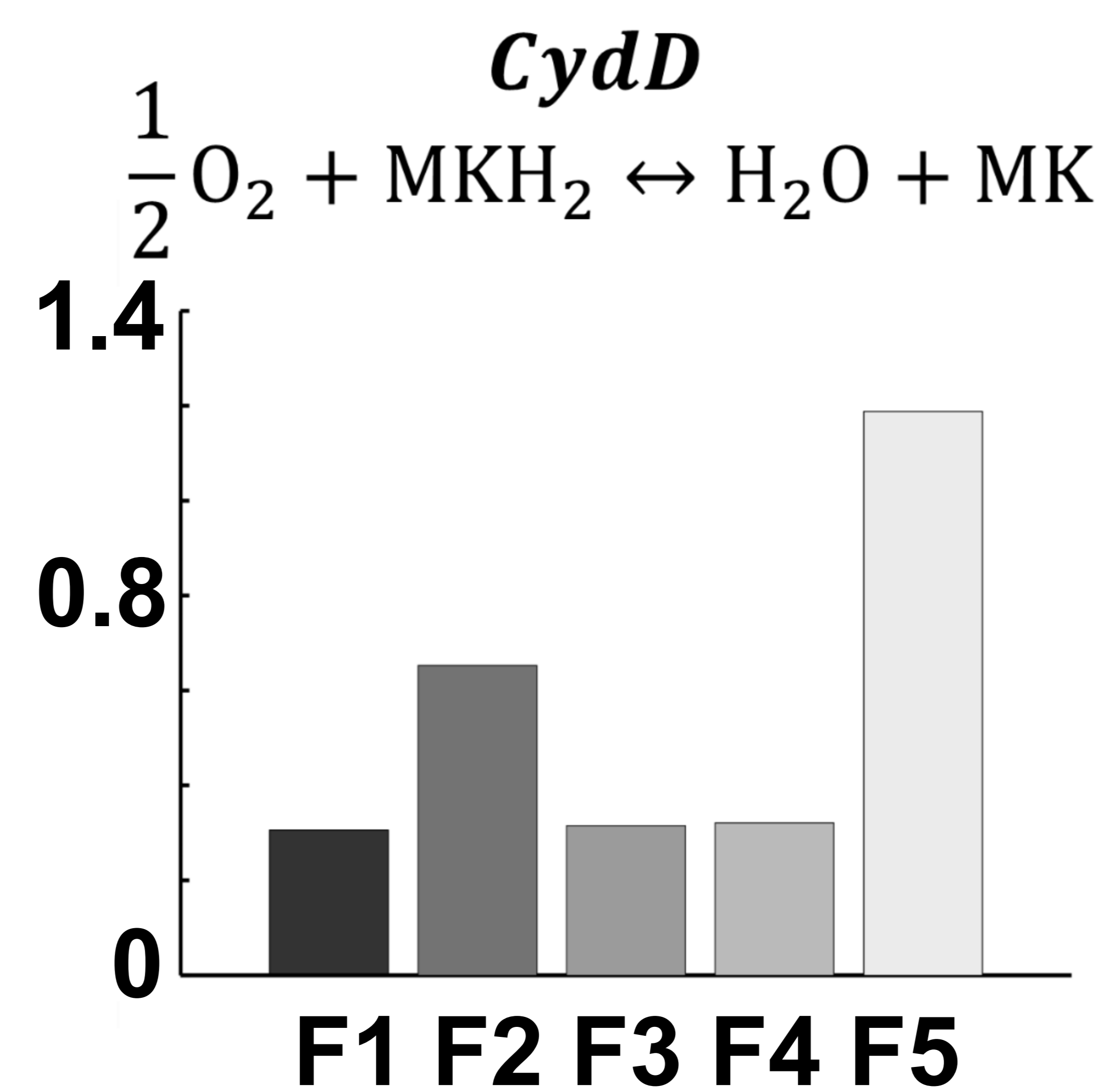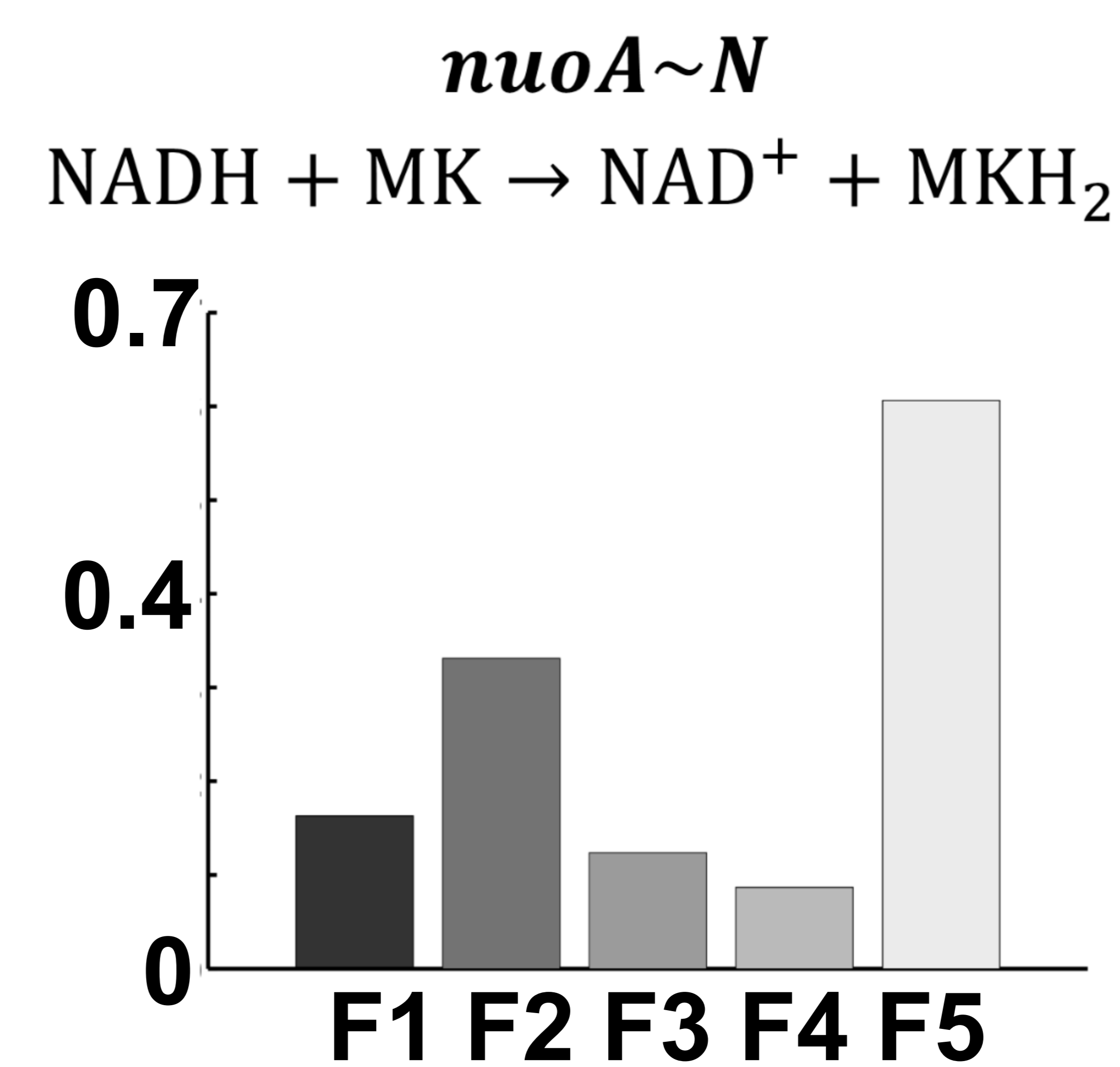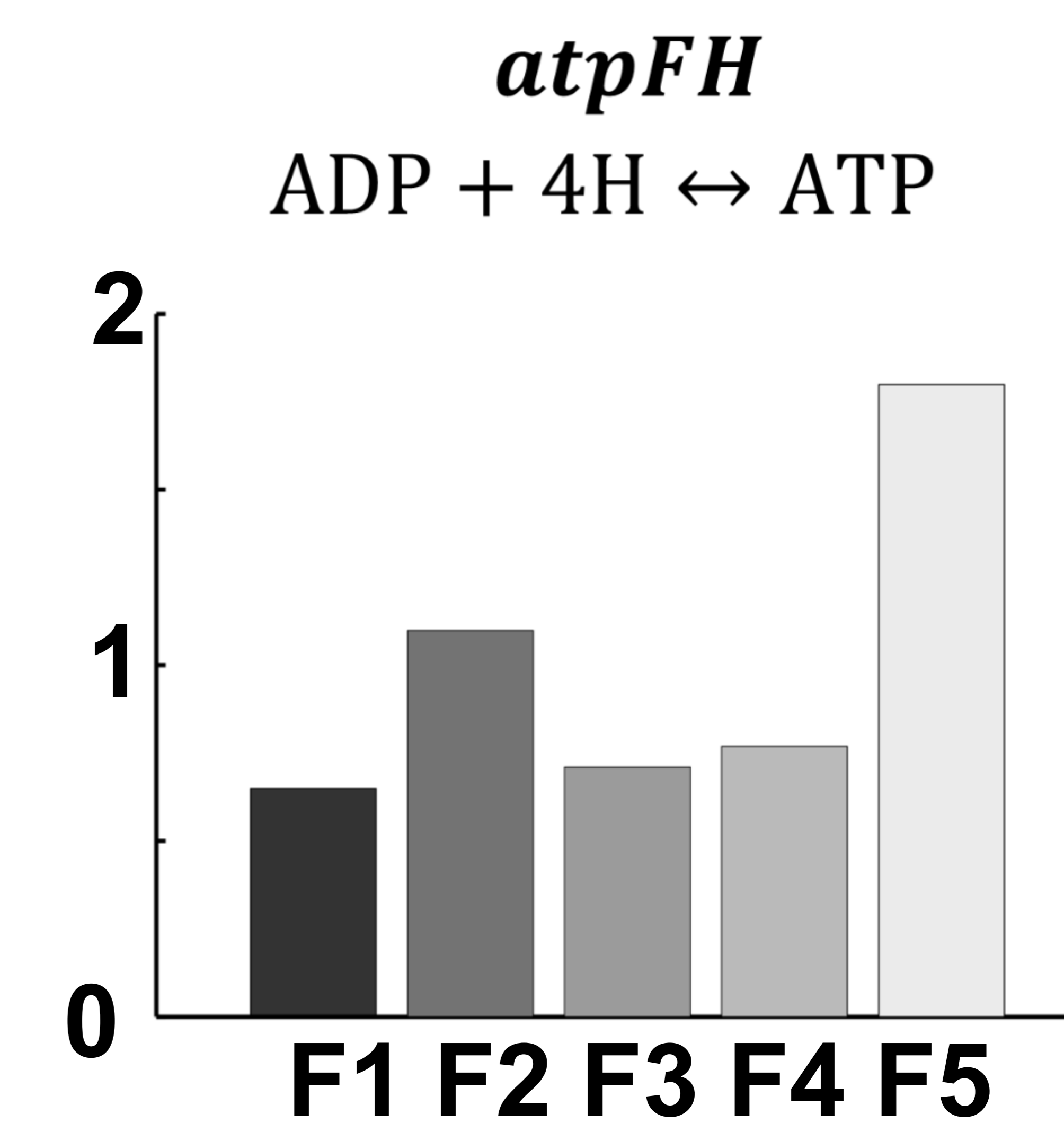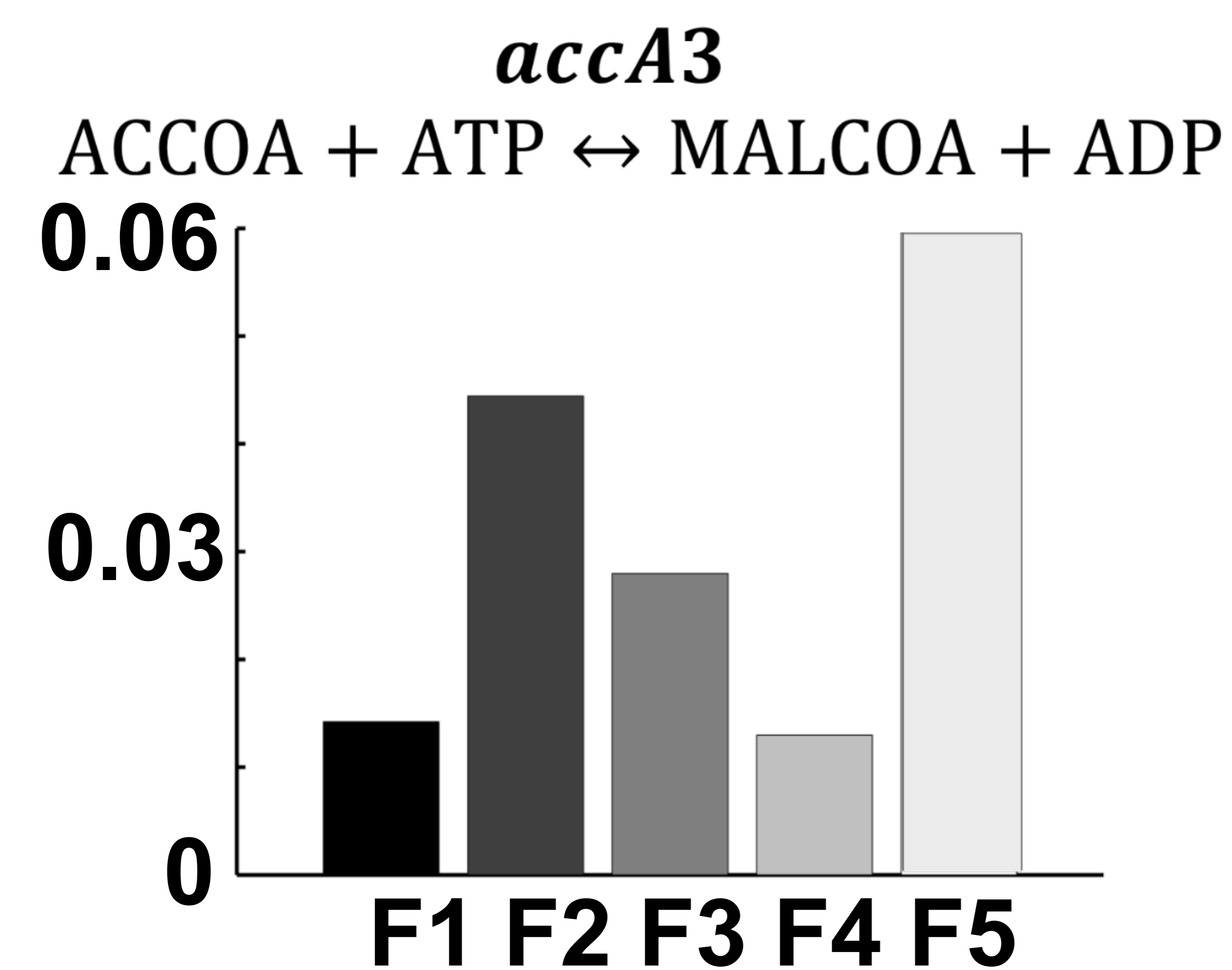

Supplement: Supplementary file 10 — Carbon flux distributions depicting additional enzymes for the icl-mutant in phases F1, F2, F3, F4, and F5. (PDF 200 kb) [file 12918_2017_496_MOESM10_ESM.pdf]

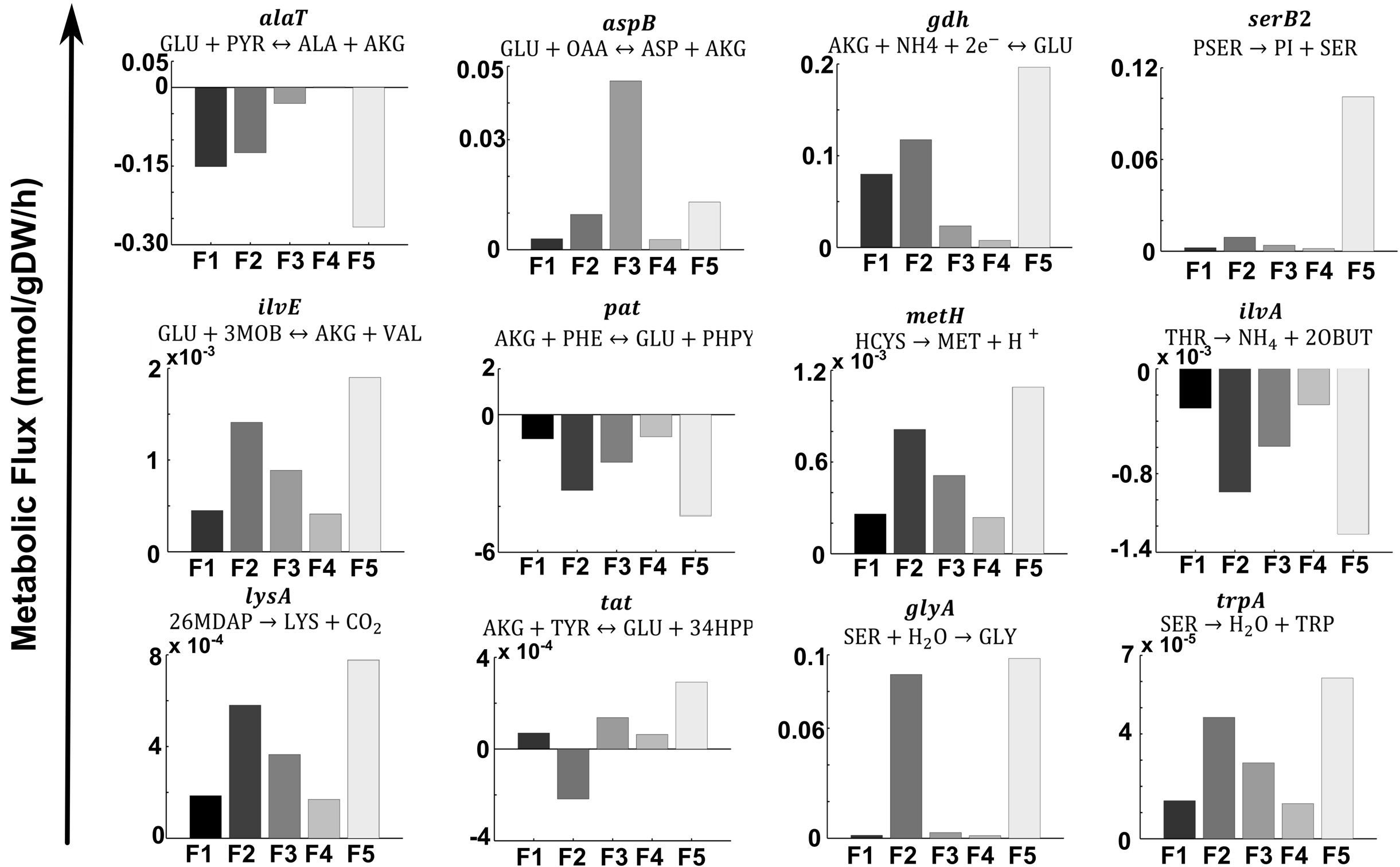

Supplement: Supplementary file 11 — Flux towards amino acid biosynthesis in phases F1, F2, F3, F4 and F5 of the pfkA-mutant. (PDF 265 kb) [file 12918_2017_496_MOESM11_ESM.pdf]

# *pfkA*-mutant - Asparagine Consumption

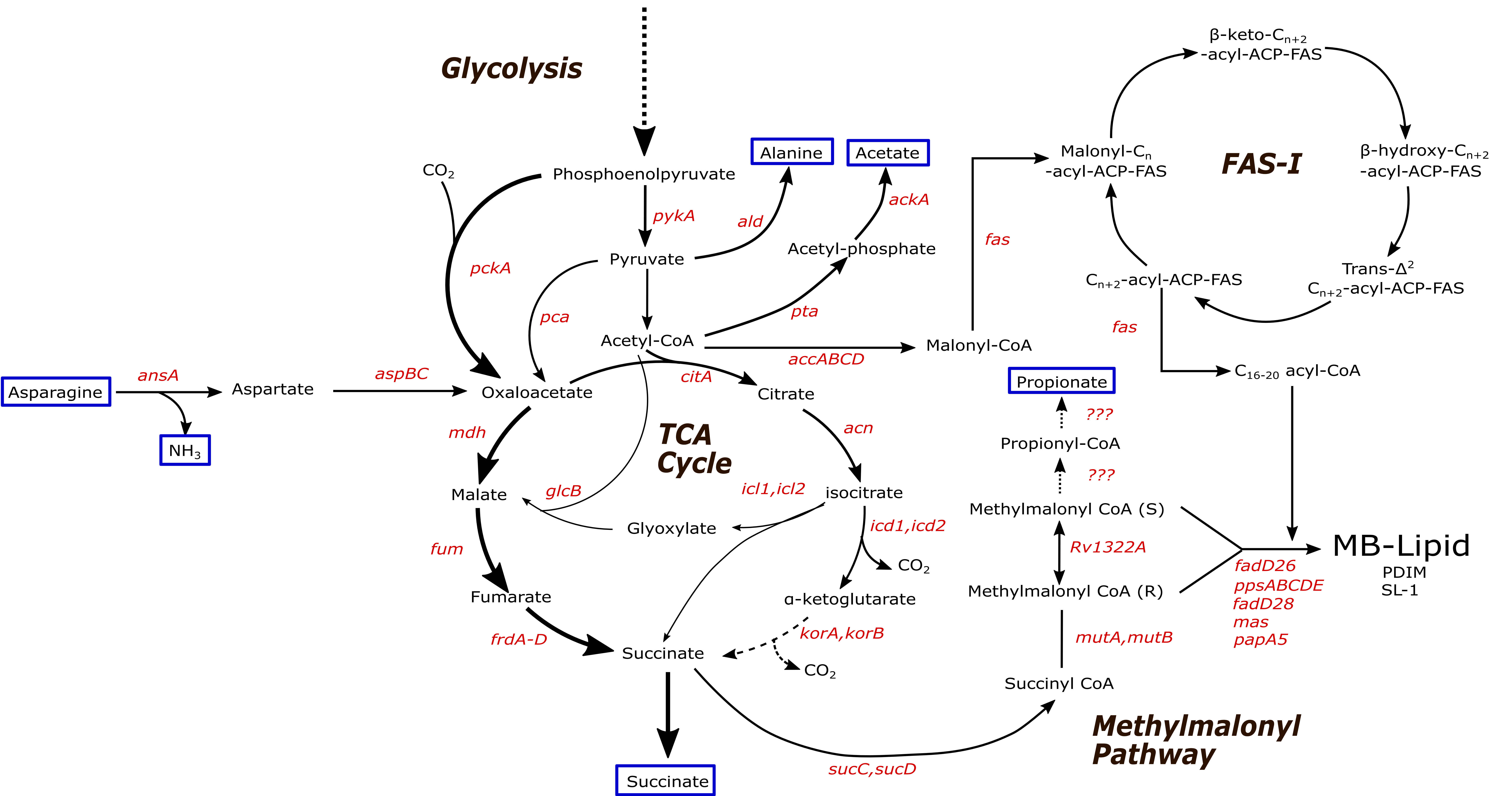

Supplement: Supplementary file 14 — Model scheme of fluxes during the metabolic adaptation the pfkA-mutant in a caseous granuloma (G2) during the consumption of asparagine. Blue boxes represent up taken and produced metabolites. (PDF 52 kb) [file 12918_2017_496_MOESM14_ESM.pdf]

# *icl*-mutant - Asparagine Consumption

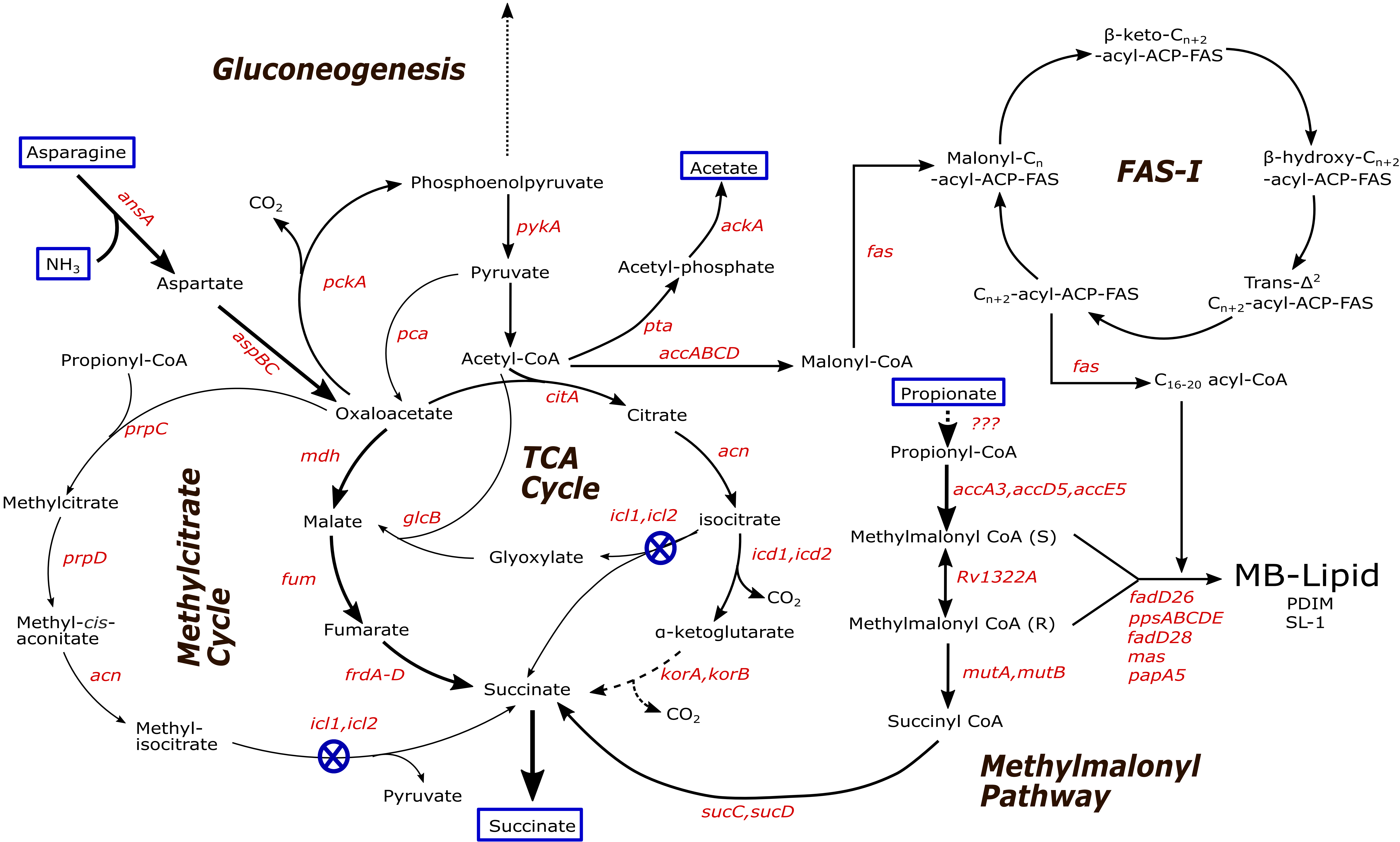

Supplement: Supplementary file 15 — Model scheme of fluxes during the metabolic adaptation of the icl-mutant in a caseous granuloma (F3/F4), during the consumption of asparagine. Blue boxes represent up taken and produced metabolites. (PDF 58 kb) [file 12918_2017_496_MOESM15_ESM.pdf]
